# Data Mining a Medieval Medical Text Reveals Patterns in Ingredient Choice That Reflect Biological Activity against Infectious Agents

**DOI:** 10.1128/mBio.03136-19

**Published:** 2020-02-11

**Authors:** Erin Connelly, Charo I. del Genio, Freya Harrison

**Affiliations:** a Schoenberg Institute for Manuscript Studies, Philadelphia, Pennsylvania, USA; b Centre for Fluid and Complex Systems, School of Computing, Electronics and Mathematics, Coventry University, Coventry, United Kingdom; c School of Life Sciences, University of Warwick, Coventry, United Kingdom; Sequella, Inc.

**Keywords:** antibiotic resistance, antimicrobial agents, antimicrobial combinations

## Abstract

The pharmacopeia used by physicians and laypeople in medieval Europe has largely been dismissed as placebo or superstition. While we now recognize that some of the *materia medica* used by medieval physicians could have had useful biological properties, research in this area is limited by the labor-intensive process of searching and interpreting historical medical texts. Here, we demonstrate the potential power of turning medieval medical texts into contextualized electronic databases amenable to exploration by the use of an algorithm. We used established methodologies from network science to reveal patterns in ingredient selection and usage in a key text, the 15th-century *Lylye of Medicynes*, focusing on remedies to treat symptoms of microbial infection. In providing a worked example of data-driven textual analysis, we demonstrate the potential of this approach to encourage interdisciplinary collaboration and to shine a new light on the ethnopharmacology of historical medical texts.

## Abstract (precis)

We used established methodologies from network science to identify patterns in medicinal ingredient combinations in a key medieval text, the 15th-century *Lylye of Medicynes*, focusing on recipes for topical treatments for symptoms of microbial infection. We conducted experiments screening the antimicrobial activity of selected ingredients. These experiments revealed interesting examples of ingredients that potentiated or interfered with each other’s activity and that would be useful bases for future, more detailed experiments. Our results highlight (i) the potential to use methodologies from network science to analyze medieval data sets and detect patterns of ingredient combination, (ii) the potential of interdisciplinary collaboration to reveal different aspects of the ethnopharmacology of historical medical texts, and (iii) the potential development of novel therapeutics inspired by premodern remedies in a time of increased need for new antibiotics.

## INTRODUCTION

Infection remedies from medieval Europe often use ingredients known to possess some antimicrobial or immunomodulatory effects, at least in *in vitro* assays (1, 2). Further, these texts suggest complex preparations of multiple ingredients and contingencies as treatments for the same symptoms. A recent study found that an early medieval remedy for eye infection used ingredients that, when combined, produced a strong antibacterial cocktail (3). The existence in the medieval pharmacopeia of groups of ingredients that are repeatedly combined to treat the symptoms of infection and that, individually, have useful biological activity gives evidence to the hypothesis that medieval medicine had some experimental basis (4, 5). It also suggests that these remedies may contain combinations of natural materials that could yield clinically useful biologically active molecules.

Network science is a powerful tool to model complex systems and extract meaningful information from data sets representing interactions between elements, such as the co-occurrence of ingredients in recipes. Network analysis has been used to characterize and analyze interactions between people or animals (6–9), computer networks (10), gene regulation networks (11), and combinations of ingredients in culinary recipes (12). The network approach represents a system as a graph, in which the nodes correspond to discrete elements (individual organisms, computers, genes, or ingredients) and the links correspond to the dyadic relations between them (communication, coordinated behavior, or colocalization) (13–16). The network paradigm provides researchers with mathematical tools to define and study the quantitative properties of real-world systems, such as the typical distribution of links among nodes (17, 18), the relation between the number of links of the nodes sharing a link (19, 20), the ability of nodes to synchronize their dynamics (21, 22), and the existence of communities (11, 23, 24). In this context, communities (also known as modules) are groups of nodes whose density of internal connections is higher than that of the links between diﬀerent modules (25).

Community structures have been identified in numerous real-world systems, where the module elements often share a function. Some typical examples are closer friendship groups within sport clubs (26), ensembles of closely collaborating jazz players (27), and sets of chemical substrates that participate in the same metabolic reactions (28, 29). Detecting such modules is one of the main techniques that are routinely employed to create organic descriptions of complex systems; a large number of algorithms have been created to perform this task (23, 24, 30–32). Here, we used community detection to analyze a set of medieval medical remedies and discover communities of ingredients that are commonly combined to treat symptoms of microbial infection.

Our work had three goals. First, we aimed to determine the tractability of turning medical texts into contextualized electronic databases amenable to quantitative analyses of hidden patterns in the use and preparation of historical *materia medica*. Network analyses appear to be eminently suited to interrogating medical texts and have successfully been used to analyze patterns of ingredient co-occurrence in culinary recipes (12). We therefore wished to determine whether community detection procedures could be used with a medieval medical text, given the challenges presented by medieval language (orthography, code switching, ambiguity in terms, and the need for the contextualization of language based on a knowledge of the medical and material culture of the period). Our second goal was to apply community detection algorithms to determine whether an exemplar text contained detectable patterns of ingredient combination and/or assignation of specific ingredients to specific symptoms of disease (in our case, microbial infection). Finally, in order to determine any antimicrobial potential of ingredients selected through the analysis of the network, those ingredients identified as core components of the network were tested for bactericidal activity against two Gram-positive opportunistic soft tissue pathogens and two Gram-negative opportunistic soft tissue pathogens.

The limited availability of medieval medical texts, as well as the constraints of researchers mining these texts by hand, has thus far meant that ethnopharmacological research conducted on medieval European medicine has concentrated on single specific ingredients (e.g., a body of literature on *Plantago* spp. [33–35]) or single recipes from select medieval texts (3, 36). The use of digital technologies to turn these texts into databases amenable to quantitative data mining requires a careful interdisciplinary approach, but it could provide a new perspective on medieval science. It could even suggest combinations of *materia medica* worthy of further study to determine their potential for drug development.

We chose as our exemplar text the 15th-century Middle English medical text known as the *Lylye of Medicynes*. This is a Middle English translation of Bernard of Gordon’s *Lilium medicinae*, originally completed in the early 14th century. The text is extant in one manuscript, University of Oxford, Bodleian Library, MS. Ashmole 1505. Bernard of Gordon was a significant medieval medical doctor and lecturer in Montpellier, France (37, 38). His magnum opus is a lengthy treatise on disease etiology, medical philosophy and history, personal case studies, treatment procedures, and recipes. The first owner of the *Lylye of Medicynes* is thought to have been Robert Broke, a distiller connected to Henry VI; appropriately, the Middle English translation is notable for its pharmaceutical content (39, 40; L. Demaitre, Translations of Bernard of Gordon’s Lilium Medicinae, ‘A booke practike to meke men’?, 2011, unpublished). There are 360 recipes in the *Lylye* specifically notated with “*Rx*.” The recipes in the *Lylye* are presented in a standard format: they begin with an indication of what type of remedy it is, such as an ointment, syrup, or plaster, and the phase of the illness during which it should be applied (beginning, middle, or end). This is followed by a recipe of ingredients, often accompanied by their quantity and preparation instructions, such as to boil, powder, or infuse. In the *Lylye* and other medieval medical texts, it was common practice to record recipes without defined measurements and to include long lists of ingredient substitutions without exact quantities, even when hazardous materials were involved. Often, specific details were not given because it was assumed that the reader was already familiar with the methodology. Furthermore, some diseases or treatments carried so many variables that the selection of ingredients and quantities was left to the judgement of the physician in charge of the patient. There are a few challenges to constructing and processing a medieval data set using modern analytical tools. These challenges include medieval spelling and language variation, multiple synonyms for the same ingredient, the translation of medieval ingredients and diseases into modern equivalents (many of these terms have multiple possible interpretations or ambiguous definitions), and the variation within the modern system of botanical binomial nomenclature.

The *Lylye* is a unique translation of a significant text by a notable medieval physician. Its pharmaceutical content, association with a medieval distiller and Tudor barber-surgeons, large number of ingredients, and chapters on infectious disease are all factors which make this text an attractive starting-off point for an analysis of antimicrobial ingredient combinations.

(This article was submitted to an online preprint archive [41].)

## RESULTS AND DISCUSSION

### Network analysis of remedies for skin, mouth, or eye infections in the *Lylye of Medicynes* reveals hierarchical communities of ingredients.

Transformation of the *Lylye of Medicynes* into an electronic database yielded 3,548 ingredients (747 unique names) used to treat 124 unique disease names, of which 41 may be classified as potential skin, mouth, or eye infections. This data set was used to construct a weighted network with 354 nodes (ingredients appearing in >1 recipe) and 3,073 weighted links (proportional to the number of times that each pair of ingredients appeared together across the recipes). It should be noted that the most frequent occurrence of ingredients (see Data Set S1 in the supplemental material) does not equate to importance from an antimicrobial perspective. For instance, references to water and sugar occur with a high frequency by a simple count. However, the use of these ingredients is to create a solution or make a medication more palatable. Water and sugar, although they occur frequently and may be counted many times in the text, are not interesting antimicrobial ingredients.

10.1128/mBio.03136-19.3 DATA SET S1 Ingredient list for recipes from the *Lylye of Medicynes*. Download Data Set S1, PDF file, 0.4 MB. Copyright © 2020 Connelly et al. 2020 Connelly et al. This content is distributed under the terms of the Creative Commons Attribution 4.0 International license.

**FIG 1:**
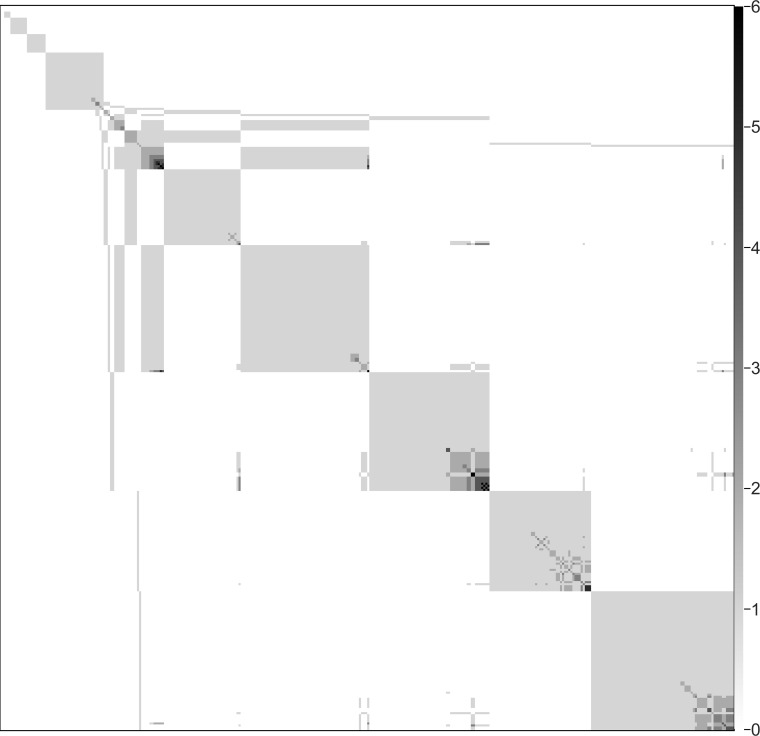
Community co-occurrence of ingredients. Every pixel in the figure corresponds to a pair of ingredients. The saturation of the color is proportional to the number of times that the pair of ingredients is found in the same community, according to the scale on the right-hand border of the figure.

To detect communities of ingredients in the network, we used the algorithm developed by Treviño et al. (32) and counted the number of times that each pair of ingredients was assigned to the same community to produce a matrix of co-occurrences. Figure 1 shows the resulting matrix as a grayscale heat map, in which the saturation of each pixel is proportional to the number of community co-occurrences of the corresponding pair of ingredients. The results clearly show the existence of a hierarchical structure within the recipes. Each larger community is composed of other, smaller subcommunities, all with a common kernel of ingredients. This allows us to identify three core combinations and four core individual ingredients. The combinations are *aloen* (aloes; Aloe vera) plus *sarcocolla nutrite* (gum which exudes from one of several Persian trees, including Penaea mucronata, P. sarcocolla, Astragalus fasiculifolius, and A. sarcocolla; *nutrite* means infused, typically in breast milk), *ceruse lote* (white lead, washed or purified) plus *eris ust* (copper calcined), and *olibanum* (frankincense) plus *sumac* (sumac; Rhus coriaria). The single ingredients are *balaustia* (blossoms of pomegranate), *galle* (bile salts), *hony* (honey), and *vinegre* (vinegar). Of these, we excluded *ceruse* and *eris* from our consideration. Although certain metals, such as copper, have shown antimicrobial properties, medicinal plants and plant-derived products were the main focus of this inquiry. Thus, we focused on the remaining ingredients.

### Identification of recipes of interest extracted from the network.

Based on the materials identified by network analysis as core individual or paired ingredients, we were able to manually select 62 recipes containing these ingredients as candidates for further investigation. These recipes were placed into seven groups in order of potential relevance (the number of core ingredients or core pairs of ingredients that occur in the recipe; Text S1). Remedies containing several alchemical metals or opiates or lengthy, complex lists of ingredients (e.g., one recipe contained 32 ingredients) were excluded. From this initial list of 62 potential recipes, the ingredients of two recipes were selected for preliminary testing of bactericidal activity. We suspect that it is likely that these two recipes are good candidates for further research.

The first recipe occurs in a chapter for the treatment of eyes, including many infectious conditions (Text S1, Group 1, Recipe 1: De Fistula in Lacrimali_unaffiliated2). This particular recipe is for the treatment of *fistula in lacrimali*, which may be a lacrimal fistula, by modern definition; it is described by the medieval text as “a fester in the corner of the eye” (*Lylye of Medicynes*, fol. 99r). The recipe is described as an ointment to “regendre flesch” (i.e., to cause new flesh to grow, to repair, or to heal). This recipe states to make an ointment of *aloen*, *olibanum*, *sarcocolla* [*nutrite*], *sumac*, and *rind of pomegarnettes* (pomegranate). The inclusion of *nutrite* was informed by comparison with two printed editions of the Latin text (42, 43) and by the frequent occurrence of *sarcocolla nutrite* throughout the Middle English *Lylye*. It is possible that *nutrite* was omitted in this recipe by scribal error or an errant (or variant) exemplar.

The second recipe occurs in the chapter *De pascionibus oris* for the treatment of pustules, ulcers, *apostemata* (swelling/inflammation/suppurating), *cancer* (a mouth sore/ulcer, wound, canker sore, tooth abscess, a cancer, or a tumor), fistula, *herpestiomenus* (gangrene, ulcer of the mouth, canker sore, pustule), and *carbunculus* (carbuncle, pustule, suppurating boil) (*Lylye of Medicynes*, fol. 125r) (Text S1, Group 2, Recipe 7: De Pascionibus Oris_unaffiliated2). This recipe is a *gargarisme* (a medicine for washing the affected area of the mouth or throat) to be used if the condition is deemed to be caused by the phlegmatic humor. The mouthwash is made of *sumac*, *galle*, *psidia* (the rind of pomegranate or the bark of the tree), *balaustia*, *mastic* (resin exuded from the mastic tree, Pistacia lentiscus), *olibanum*, *hony*, and *vinegre*.

### Structured review of evidence for efficacy of selected ingredients.

To perform a thorough literature search for evidence of the antimicrobial or immunomodulatory effects of the ingredients identified as core components of the network, we (i) searched the *Cochrane Database of Systematic Reviews* for reviews containing each ingredient’s name and (ii) conducted a wider literature search using Scopus [TITLE-ABS-KEY (ingredient_name) AND TITLE-ABS-KEY (antibacterial OR antifungal OR antibiotic OR antimicrobial)]. For plant species, common names and Latin binomials were searched; further terms were added as necessary to specifically search for active compounds within ingredients (e.g., lactoferrin for breast milk). Searches were conducted during the week of 25 to 29 October 2017 and updated on 1 November 2019, following initial peer review of the manuscript. In empirical literature on the antimicrobial effects of ingredients of component compounds, we assessed the rigor and relevance of the results. This included not only a judgment on whether sufficient quantitative data were presented but also an assessment of the relevance of experiments to an *in vivo* context; i.e., we judged experiments testing the effects of substances on bacteria growing as a biofilm (44) to be more relevant than those using disk diffusion assays on agar plates or inhibition of planktonic growth and judged those using clinical isolates of bacteria to be more relevant than those focusing purely on domesticated lab strains of the same bacteria.

A structured literature review found poor evidence of the useful antimicrobial or healing effects of aloe, frankincense, mastic, and sarcocolla. Aloe extracts are used in a wide variety of medicinal and cosmetic products marketed for skin healing, and numerous patents cover the use of acetylated mannans from Aloe vera due to their supposed immunomodulatory effects (45, 46). However, two recent Cochrane reviews found that published studies on the wound healing and antiseptic properties of this plant were generally of low quality and that the results of human trials that have been published returned variable results, spanning from help and harm to the patients (47, 48). The available literature on the biological composition and activity of mastic was even less clear. This resin has a history of use as a chewing gum to promote oral health and does appear to contain compounds with the ability to inhibit bacterial growth in disk diffusion assays *in vitro* (49). However, we found that the few publications available which specifically addressed the effect of mastic gum on oral bacteria did not present clear quantitative data on the effects of mastic on bacterial cell viability (50–53). Frankincense may be obtained from a number of trees in the genus *Boswellia*, most usually, Boswellia sacra. It may have some immunomodulatory and, thus, healing effects via its effects on the NF-κB and tumor necrosis factor alpha pathways, but little research on its chemistry has been conducted; this is further complicated by the fact that different species have different chemical compositions and crude extracts can have effects on inflammatory pathways different from those of the purified constituent molecules (54, 55). One recent study compared the chemical composition and bactericidal activity of frankincense with those of other plant essential oils and found it to be very poorly antimicrobial (56). Finally, while the medicinal use of sarcocolla is mentioned in historical sources, we were unable to find any modern research into its biological properties.

Slightly better evidence suggesting the antibacterial potential of vinegar, sumac, bile, and pomegranate rind was found. Vinegar is generally acknowledged to act as a mild disinfectant, and pure acetic acid has been shown to exhibit various bactericidal effects at concentrations similar to or lower than those typically found in vinegar. This includes the ability to kill cells of key opportunistic pathogens living as monospecies biofilms (clinical isolates of the Gram-negative species Escherichia coli, Pseudomonas aeruginosa, Acinetobacter baumannii, Klebsiella pneumoniae, Enterobacter cloacae, and Proteus mirabilis, plus the Gram-positive species Staphylococcus aureus [57, 58]). As a weak acid, it can readily cross cell membranes and collapse the cross-membrane proton gradient necessary for ATP synthesis; once inside the cell, acetic acid alters the cytoplasmic pH, and this can cause DNA damage and protein unfolding (58). A clinical trial into the effectiveness of acetic acid for treating burn wound infections is under way in the United Kingdom (59). Vinegar has also been shown to kill the fungus Candida albicans, the causal agent of oral thrush, *in vitro* (60). Like vinegar, an aqueous extract of sumac is acidic (61); this property in itself could provide some antibacterial effect, but even after increasing the pH to neutrality, aqueous extracts of dried sumac fruit show bactericidal effects against Gram-positive and Gram-negative bacteria grown as planktonic cultures, and this has been partly attributed to tannins (61, 62). The bactericidal effects of bile are long established: bile salts disrupt bacterial membranes, and this quality contributes to host-mediated control of bacterial overgrowth in the gut (63, 64). Interestingly, different fractions of aqueous or alcohol extracts of pomegranate peel have different spectra of antibacterial activity, as measured in disk diffusion assays, and different authors present conflicting data on the inhibitory effects of aqueous extract fractions (65–70). We note, however, that antibacterial effects have been observed, even when using dried rather than fresh peel, and that pomegranate peel extracts have also been reported to exhibit antiviral (71) and antifungal (66, 67, 70) effects. We were unable to find any relevant data on the *in vivo* effects of these four ingredients with regard to treating soft tissue wounds or infections, except for one small study which compared the healing of excisional wounds in mice treated topically with either gentamicin ointment or pomegranate extract: in this study, all mice treated with pomegranate extract showed complete wound healing (72).

The most interesting ingredients are undoubtedly honey and breast milk. Here we must add the caveat that honey’s sweetness must explain a large part of its ubiquity in premodern medicine: a mouthwash containing gall, vinegar, and sumac would certainly be unpalatably bitter in the absence of a sweetening agent! However, the antimicrobial and wound-healing properties of honey have been relatively extensively studied, and honey preparations and dressings are used in clinical settings (e.g., see the recommendations of the U.K. National Health Service [73]). Honey’s antimicrobial, immunostimulatory, and healing effects are attributed to its osmotic properties, an enzyme that produces hydrogen peroxide, bee defensin, phenolics, and flavonoids: while the most potent medical-grade honey (manuka honey) also contains highly active compounds from the plant from which it is made, a range of European honeys have been shown to have measurable antimicrobial effects *in vitro* (74–77). Cochrane reviews found moderate to good evidence that honey reduces healing times for burns and surgical wounds (48, 78) and weak evidence that honey helps to prevent mouth ulcers in cancer patients (79), and another Cochrane review suggested that honey may be better than no treatment or placebo in relieving childhood cough (80).

Breast milk is similarly recognized to be antimicrobial. It contains lysozyme, an enzyme which destroys the structural peptidoglycan layer surrounding bacterial cells and which is recognized to be a key component of innate immunity (81). It also contains oligosaccharides which can inhibit bacterial growth and biofilm formation and sensitize them to antibiotics via membrane permeabilization (82–85). Further, it contains the protein lactoferrin, which sequesters vital iron away from pathogenic bacteria. Under acid conditions, lactoferrin is cleaved to produce a peptide called lactoferricin, which has a direct bactericidal effect by binding cell walls and triggering membrane damage (81, 86, 87). Lactoferrin can kill a range of microbes, including antibiotic-resistant K. pneumoniae strains and *Candida* spp.; it can also enhance the sensitivity of these microbes to modern antibiotics (86, 87). Clinical research into the antimicrobial potential of breast milk or its component proteins is, however, lacking (88). It is interesting that donkey milk is suggested to be a substitute (*Lylye of Medicynes*, fol. 16v), as the proteome of donkey milk is very similar to that of human milk, and donkey milk contains appreciable amounts of lactoferrin and lysozyme (89, 90). Raw breast milk also contains a range of commensal bacteria, and if milk was not prepared in such a way that these were killed, it is possible that the milk microbiome could contribute to its anti-infection properties. One study found that colostrum could help alleviate infant conjunctivitis because the commensal bacteria in the milk outcompeted the pathogenic bacteria when applied to the eye (91).

There are several reasons why mixing these ingredients could represent a rational medical decision. First, combining ingredients in a cocktail could increase efficacy against a particular target microbial species by attacking several cellular targets at the same time or allowing for the chemical activation of particular component molecules. For instance, acid pH promotes the proteolysis of lactoferrin into peptides called lactoferricins, which have greater antimicrobial activity than the original protein (92). When milk is directly consumed, this happens in the gut and inside neutrophils, but we note that the sumac in the recipe for the treatment of *fistula in lacrimali* would decrease the pH of the recipe and potentially process lactoferrin into lactoferricins in the ointment itself. A further possibility is that using multiple ingredients allows for contingency. We note that the available evidence suggests, for instance, that Gram-positive bacteria seem to show more sensitivity to sumac than Gram-negative bacteria, whereas the opposite is true for vinegar (58, 60). Another type of contingency which could be covered by combining multiple antimicrobial ingredients would be protection against potential variation in the composition and the quality of particular ingredients; e.g., which *Boswellia* species was the available frankincense sourced from, and did this vary (54)? Finally, we must also consider the physical properties of each ingredient. Mastic, for instance, is not well evidenced as an antimicrobial but could act as a thickening agent to work other, active ingredients into an ointment suitable for topical application to an infection site.

### Pilot testing of core ingredient combinations for antibacterial activity.

Based on the information presented above, we conducted preliminary tests to assess the antibacterial potential of honey, acetic acid, and bovine bile plus a 1:1 mixture of frankincense essential oil and sumac aqueous extract and a 1:1 mixture of breast milk and Aloe vera sap, alone and in pairwise combination. We also tested the effect of the combination of these ingredients present in the remedy for *pascionibus oris*: a 1:1:1:1:1 mix of bile, acetic acid, frankincense, sumac, and honey preparations. Each agent or combination thereof was added in triplicate to planktonic cultures of two Gram-positive opportunistic soft tissue pathogens (Staphylococcus aureus, Enterococcus faecalis) and two Gram-negative opportunistic soft tissue pathogens (Pseudomonas aeruginosa, Escherichia coli) grown in medium designed to mimic fluid in soft tissue wounds. As shown in Table 1, acetic acid and frankincense caused the complete killing of all four exemplar bacteria in planktonic culture, and for acetic acid, this effect was preserved when the acetic acid was diluted 1/5; given this result, together with the ability of honey to kill three of the four exemplar pathogens at a 20% dilution, it is unsurprising that the reconstituted remedy for *pascionibus oris* completely killed all four pathogens. However, when the effects of single versus paired agents were compared in Table 2, some interesting results were observed. The combination of Aloe vera and breast milk could interfere with the killing effects of honey (for P. aeruginosa and E. coli) or those of frankincense plus sumac (for P. aeruginosa, E. coli and S. aureus); however, in the case of E. faecalis, combining bile with honey or combining frankincense-sumac with honey led to the synergistic emergence of bactericidal activity. The strong activity of acetic acid and honey may mask any further synergistic effects in this very simple planktonic killing assay, in which we expect bacteria to be relatively sensitive to antibacterial agents, but we would suggest that combinations of honey, acetic acid, bile, and frankincense and/or sumac may be worth investigation for their ability to potentiate each other’s antibacterial effects in biofilm models of infection, where the combinatorial activity of agents is more likely to enhance the killing of bacteria with enhanced tolerance.

**TABLE 1:** Effects of selected individual agents, two core combinations, and the reconstituted remedy for *pascionibus oris* from the *Lylye of Medicynes* on viability of four bacterial pathogens in planktonic culture

Agent(s)	Dilution	Activity against^*a*^:			
		S. aureus	E. faecalis	P. aeruginosa	E. coli
Honey	As prepared	−	++	−	−
	1/5	−	++	−	−
Acetic acid	As prepared	−	−	−	−
	1/5	−	−	−	−
Bile	As prepared	++	++	++	++
	1/5	++	++	++	++
Aloe vera	As prepared	++	++	++	++
	1/5	++	++	++	++
Breast milk	As prepared	++	++	++	++
	1/5	++	++	++	++
Sumac	As prepared	−	++	++	−
	1/5	++	++	++	++
Frankincense	As prepared	−	−	−	−
	1/5	−	++	++	++
Aloe vera + breast milk	As prepared	++	+	++	++
Frankincense + sumac	As prepared	−	+	−	−
*Pascionibus oris* remedy	As prepared	−	−	−	−

a ++, no reduction in viable bacteria was observed in any of the three replica cultures; +, at least a 1-log reduction was observed in all three replica cultures; −, complete killing was observed in all three replica cultures.

**TABLE 2:** Comparison of effects of individual agents versus effects of pairs of selected agents from the *Lylye of Medicynes* on viability of four bacterial pathogens in planktonic culture

Species and agent(s)	Activity of^*a*^:				
	Honey	Acetic acid	Bile	Aloe vera + breast milk	Frankincense + sumac
S. aureus					
Honey	−	−	−	−	−
Acetic acid		−	−	−	−
Bile			++	++	−
Aloe vera + breast milk				++	++^*b*^
Frankincense + sumac					−
E. faecalis					
Honey	++	−	−^*c*^	++	−^*c*^
Acetic acid		−	−	−	−
Bile			++	++	++
Aloe vera + breast milk				+	++
Frankincense + sumac					+
P. aeruginosa					
Honey	−	−	−	++^*b*^	−
Acetic acid		−	−	−	−
Bile			++	++	NA
Aloe vera + breast milk				++	++^*b*^
Frankincense + sumac					−
E. coli					
Honey	−	−	−	++^*b*^	−
Acetic acid		−	−	−	−
Bile			++	++	++^*b*^
Aloe vera + breast milk				++	++^*b*^
Frankincense + sumac					−

a ++, no reduction in viable bacteria was observed in any of the three replica cultures; +, at least a 1-log reduction was observed in all three replica cultures; −, complete killing was observed in all three replica cultures; NA, variable results were observed in the three replicates.

b The paired agents had potential interference.

c The paired agents had potential synergistic activity.

### Conclusions and future directions.

Turning a historical medical text into a database suitable for network analysis was achieved through detailed interdisciplinary consideration of the text, its context, and its structure. The outcome of this study was dependent on the collaborative efforts and exchange of knowledge between multiple disciplines: medieval studies to translate and contextualize recipes, ingredients, and symptoms; mathematics and computer science to organize and analyze the resulting database; and microbiology/immunology to determine if blind mathematically driven results make sense from an applied perspective.

This work demonstrates the possibility to use algorithms from complex networks to explore a medieval medical data set for underlying patterns in ingredient combinations related to the treatment of infectious disease. The preliminary results from this pilot study of a single medieval medical text speak to larger questions regarding medieval medical rationality in constructing treatment recipes and the application of historical medical knowledge to the current search for novel antimicrobial drugs. Application of this methodology to an expanded data set of ingredients drawn from multiple representative medical and surgical sources may provide a foundation to begin to build evidenced answers to such questions. While we must be careful in interpreting and extrapolating the results of the laboratory assays performed on the ingredients of interest, these preliminary tests are a useful foundation for future, more detailed experiments.

## MATERIALS AND METHODS

### Building a database from the *Lylye of Medicynes*.

As a starting point, the 360 recipes of the *Lylye* were entered into a spreadsheet (see Data Set S1 in the supplemental material). These recipes contain 3,374 individual ingredient names for the treatment of 113 medical conditions. Of those conditions, 30 (containing 86 recipes) may be classified as infections which may be targeted with topical treatments, mainly infections of the skin, mouth, or eyes. The *Lylye of Medicynes* uses language which clearly indicates *infection*, as it might be translated in modern terms. For example, in describing skin swellings, abscesses, ulcers, wounds, carbuncles, erysipelas, and fistula, symptoms from the text include broken skin, purulence, itching, foul smell, heat, moisture, aching, pricking and burning sensations, redness, yellowness, ulceration, and black crusts, all of which are indicators of infection (*Lylye of Medicynes*, Book 1, fols 24r to 28v).

In addition to the 360 recipes identified by the text with “*Rx*,” there are multiple other ingredients that are not prefaced by *Rx* but that are clearly set up in the format of a recipe. To the principal data set, 36 additional recipes containing 174 ingredients for the treatment of 19 medical conditions were added for a total data set of 3,548 ingredients (747 unique names) and 124 unique disease names, of which 41 may be classified as potential infections of the skin, mouth, or eyes. Ingredients which were excluded from the data set were those not in recipe format, such as lists of ingredient substitutions and lists of purgative ingredients.

Spelling variants were normalized by hand. The frequency of occurrence in the text of the *Lylye* and the headword used in the *Middle English Dictionary* (*MED*) (93) were taken into consideration when normalizing the spelling. Additionally, preference was given to English forms over Latinate forms. For instance, *rote* was selected over *radicis* and *radix*. The Middle English characters of thorn and yogh were modernized. The Middle English usage of *u*/*v* and *i*/*j* were also standardized to modern practices. To eliminate redundancy and to aid with the processing of our data set, synonyms were combined under one term, with preference being given to the Middle English term appearing most frequently in the *Lylye* or as the headword in the *MED*. For instance, *vinegre* was chosen as the form for many textual variants, such as *venegre*, *vynegre*, *acetum*, *aceto*, *aceti*, and so forth. Due to variations in active ingredients in different parts of a plant, those designations were included (e.g., root, seeds, juice, flowers, rind). The imperative of this study was to identify significant ingredient combinations. Therefore, preparation instructions were not included with the ingredient names, except in cases where the preparation changed the nature of the base ingredient (e.g., powder, syrup, calcined). The Middle English ingredient names and disease names were not translated into modern English in the spreadsheet or for the analyses. Modern English translations are provided for the terms referenced in this article. As many medieval medical terms are ambiguous and definitions vary between dictionaries, caution was used in translating these terms into modern English and in identifying the scientific names of plants. The definitions were cross-checked using the *MED*, *Oxford English Dictionary* (94), the *Dictionary of Medical Vocabulary in English*, *1375-1550* (95) (of which the *Lylye* served as an example text), the Kew Medicinal Plants database (96), and the Plant List database (97). Normalizing or cleaning a medieval data set for analysis with modern computational tools is not a standardized methodology (98–101; see also http://ucrel.lancs.ac.uk/vard/publications/). In documenting our approach to this data set, we hope to add to other ongoing conversations about structuring medieval data in a modern digital space.

### Network construction and community detection.

**FIG 2:**
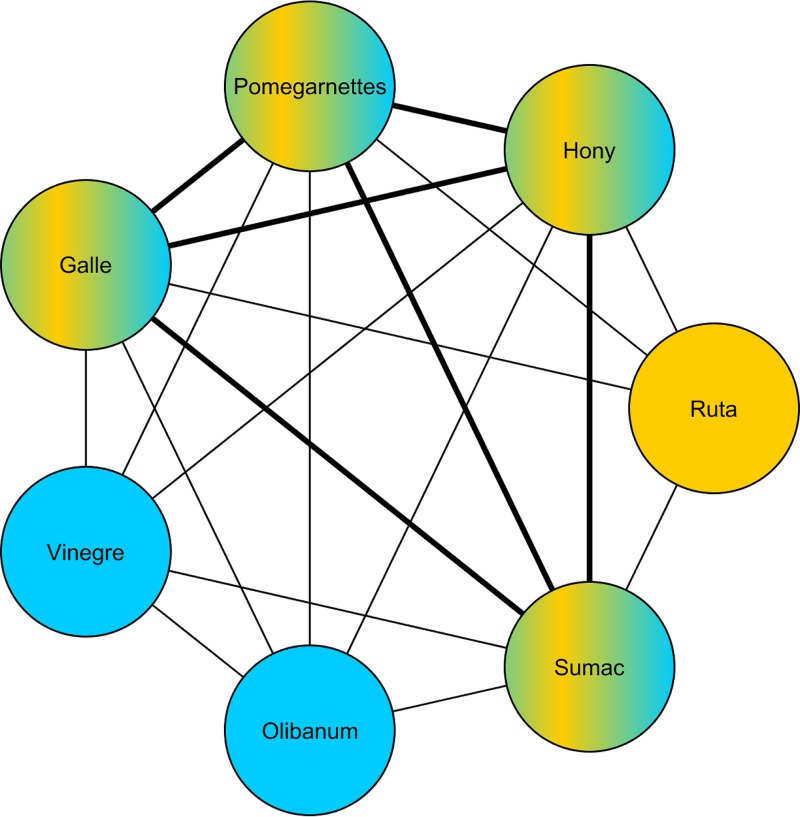
Example of an ingredient network. The nodes in yellow are ingredients of a recipe for the treatment of *fistula in lacrimali*; those in blue are ingredients of a recipe for the treatment of *pascionibus oris*. Ingredients found in both recipes are colored both yellow and blue. Thicker links join pairs of ingredients that appear in both recipes.

To build a network from the recipes, we created a node for each individual ingredient encountered. Every time that two ingredients were found together in the same recipe, a link was placed between them. In cases in which pairs of ingredients were found in more than one recipe, the corresponding links were strengthened by assigning them a weight proportional to the number of times that the pair occurred across recipes. For example, if ingredients *A* and *B* occurred together in 10 different recipes, the weight of the link between the corresponding nodes would be 10. As a visual aid, Fig. 2 shows the network created from one recipe for the treatment of *fistula in lacrimali* (lacrimal fistula) and one for the treatment of *pascionibus oris* (diseases of the mouth) (Text S1, Group 5, Recipe 20: De Fistula in Lacrimali_unaffiliated1 and Group 2, Recipe 7: De Pascionibus Oris_unaffiliated2). The ingredients for the former (shown in yellow) are *galle*, *hony*, *pomegarnettes*, *ruta* (a plant of the genus *Ruta*, especially the common rue, Ruta graveolens), and *sumac*; those for the latter (shown in blue) are *galle*, *hony*, *olibanum*, *sumac*, *vinegre*, and *pomegarnettes*. Note that four ingredients, namely, *hony*, *galle*, *pomegarnettes*, and *sumac*, are found in both recipes. Thus, the corresponding nodes are colored both yellow and blue. Since, in this example, six pairs of ingredients are found in both recipes, the links that represent them are thicker.

To normalize the link weights, we used a linear transformation that did not aﬀect the network properties (102, 103). More specifically, if nodes *i* and *j* were connected, their link weight ($W_{i,j}^{\text{new}}$) transformed as

(1) $$W_{i,j}^{\text{new}}=\frac{\left(1-2\text{ε}\right)W_{i,j}+\left(2\text{ε}-1\right)W_{\text{min}}}{W_{\text{max}}-W_{\text{min}}}+\text{ε}$$

where *W*_min_ and *W*_max_ are the minimum and maximum original weights, respectively, and ε is a small parameter used to avoid cutting links whose original weight is *W*_min_. In our case, we used the common choice of ε equal to 0.01. Its effect is to transform the link weights, maintaining their ratios and making the smallest weight equal to ε and the largest one equal to 1 − *ε*. In principle, one could perform the analyses of ingredient communities on the full weighted network. However, the results could be aﬀected by noise in the data, such as noise that could be originated by pairs of ingredients whose links were too weak to be significant but strong enough to spuriously alter the local structure of the network. To avoid this type of problem, we introduced a variable threshold, *t*. Starting from the full weighted network, for every given value of *t*, we created a thresholded, unweighted network by removing all the links whose weight was less than or equal to *t* and considering all other links as having equal unitary weight. By studying how the communities change with the value of the threshold, we were able to find groups of ingredients that always or most often belonged to the same community, regardless of the assignment of the other nodes around them. This allowed us to identify combinations of ingredients that are relevant and that thus warrant further investigation.

**FIG 3:**
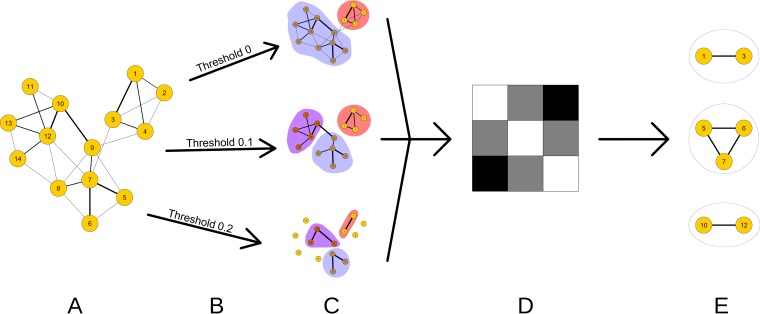
Fictitious example of the process for identifying relevant combinations of ingredients via thresholding and community detection. (A) The starting network. (B, C) Apply the thresholding procedure and choose the partitioned network with maximal modularity (*q*_{_*_c_*_}_, equation 2). (D) Draw a heat map to visualize the strengths of associations between ingredient pairs. Darker pixels indicate a stronger association. For example, in this example, network ingredients 1 and 3 are more strongly connected by their recipe co-occurrences than are ingredients 1 and 2 or ingredients 2 and 3. (E) Identify the most strongly associated group of ingredients and manually search the database for recipes including these combinations.

To illustrate the procedure, consider the fictitious network in Fig. 3. For threshold 0, we identify two communities (red and blue), while for thresholds 0.1 and 0.2, we find three communities (red, blue, and violet). Note that, in going from threshold 0 to 0.1, the blue community splits into two subcommunities (blue and violet). This is an example of a partially hierarchical structure: some communities are themselves composed of other, smaller communities. Comparing the results for the three thresholds, we find the combinations of nodes 1 and 3; nodes 5, 6, and 7; and nodes 10 and 12 to be important. Note that, in the original network, nodes 5 and 6 are connected with the weakest (thinnest) link. Nonetheless, this procedure identifies their co-occurrence, together with ingredient 7, as potentially relevant. Also, node 9 belongs to the same community as nodes 10 and 12 at the highest threshold, but it is excluded from their group in the final result as this community coparticipation does not hold for all thresholds.

We chose 6 thresholds: 0, 0.01, 0.15, 0.29, 0.43, and 0.571. Our choice was based on the fact that the number of edges that are cut increases with *t*, causing more nodes to become isolated and the network to fragment (Fig. S1). Thus, we chose the values at which such changes in the thresholded network happened, stopping at 0.571 because for that threshold the number of nonisolated nodes becomes only 10.

10.1128/mBio.03136-19.1 FIG S1 Fragmentation of the network with a threshold. As the threshold is increased, more nodes lose all their links and become isolated. Download FIG S1, TIF file, 0.1 MB. Copyright © 2020 Connelly et al. 2020 Connelly et al. This content is distributed under the terms of the Creative Commons Attribution 4.0 International license.

To carry out the community detection on our data, we used the algorithm developed by Treviño et al. (32). This is a spectral method that uses a synergy of refining steps, including local and global tuning, as well as agglomeration, to estimate the network partition that maximizes modularity, a widely used objective function. For a network with *N* nodes and *m* links, its partition into a set of communities has modularity (*q*_{_*_c_*_}_)

(2) $$q_{\left\{c\right\}}=\frac{1}{2m}\sum \left(A_{i,j}-\frac{k_{i}k_{j}}{2m}\right){\text{δ}}_{c_{i}c_{j}}$$

In the expression in equation 2, *A* is the adjacency matrix of the network, whose elements *A_i_*_,_*_j_* are 1 if nodes *i* and *j* are linked and 0 otherwise; *k_i_* and *k_j_* are the number of links involving nodes *i* and *j*, respectively; *c_i_* and *c_j_* are the communities to which nodes *i* and *j* belong, respectively; δ is Kronecker’s symbol; and the sum is over all possible pairs of nodes. Thus, this definition promotes partitions in which the number of links within communities is higher than its expectation value for a randomized version of the network. So, a partition of a network that maximizes this quantity corresponds to a set of communities whose constituent nodes are more strongly or more densely connected among themselves than they are with nodes belonging to other communities, thus formalizing the intuitive sense of a modular structure.

For each value of the link threshold, we ran our community detection algorithm 10,000 times and selected the partition with the highest modularity. Having obtained the best community structures, we counted the number of times that every possible pair of ingredients was assigned to the same community, producing a matrix of co-occurrences. To visualize this matrix, we sorted the ingredient list using the Cuthill-McKee algorithm (104) and represented the result as a grayscale heat map, in which the saturation of each pixel of coordinates (*i*, *j*) is proportional to the number of community co-occurrences of ingredients *i* and *j.*

### Testing ingredients for bactericidal activity.

Ingredients identified as core components of the network were tested for activity against two Gram-positive opportunistic soft tissue pathogens (Staphylococcus aureus ATCC 29213, Enterococcus faecalis ATCC 29212) and two Gram-negative opportunistic soft tissue pathogens (Escherichia coli ATCC 25922, Pseudomonas aeruginosa PAO1).

Honey was locally sourced (Tomlow heather honey; Thurlong Farm, Stockton, Warwickshire, United Kingdom); on the day of testing, 9 g honey was aseptically weighed into a sterile Falcon tube and 1 ml sterile water was added to generate a 90% honey solution. This solution was warmed by immersing the tube in warm water until it was pipettable. Dried bovine bile (Sigma-Aldrich) was reconstituted in water to a physiological concentration of 89 mg·ml^−1^ and sterilized with UV in case of contamination during industrial manufacture; fresh bile solution was prepared on the day of testing. Fresh breast milk was supplied on the day of testing by a healthy volunteer and stored at room temperature. Vinegar was represented by a 6% (vol/vol) solution of acetic acid in water, as pilot data suggested that there was no difference in bactericidal activity between 6% acetic acid and two exemplar table vinegars with 6% acidity (Waitrose Essential red or white wine vinegar). Sumac extract was prepared in advance of testing following a published method (61). Al’Fez sumac (5 g; no salt) was added to 95 ml sterile distilled water and gently stirred for 1 h at 20°C, followed by gentle boiling for 2 min. The solution was then cooled, passed through a 0.22-μm-pore-size filter, and stored at −20°C until needed. Aloe vera sap was prepared in advance of testing by crushing the leaves with a pestle and mortar and adding 10 ml sterile water to 15 ml sap to render it pipettable. The solution was stored at −20°C until needed. A 20% frankincense solution was obtained by dissolving Boswellia sacra essential oil (Neal’s Yard organic frankincense essential oil) in ethanol; we found that diluting to 20% was necessary to prevent the essential oil from melting laboratory plasticware. Mixtures (1:1) of Aloe vera sap and breast milk and of frankincense and sumac extract were made, as these were identified to be key combinations in the *Lylye of Medicynes* text. These paired ingredients were henceforth each treated as a single agent. The combination of these ingredients present in one exemplar remedy (for *pascionibus oris*) was also reconstituted and consisted of a 1:1:1:1:1 mix of bile, acetic acid, frankincense, sumac, and honey preparations. Thus, to assess the effect of combined versus single ingredients while controlling for concentration, it was necessary to test each individual ingredient undiluted and after dilution 1/5 in sterile water (as each would be present at a 1/5 dilution when combined into the remedy for *pascionibus oris*).

One hundred microliters of each agent to be tested (neat individual ingredients, 1/5-diluted individual ingredients, and mixtures representing treatments for *pascionibus oris* and *fistula in lacrimali*) was added in 12-fold replication to the wells of a 96-well microplate. Bacterial strains were prepared to concentrations of 1 × 10^6^ CFU per ml by making 0.5 McFarland dilutions in phosphate-buffered saline and diluting these 100-fold in synthetic wound fluid. Synthetic wound fluid comprised a 50% fetal bovine serum (Gibco) source, 50% peptone water (Sigma-Aldrich) (105). One hundred microliters of each bacterial suspension was added to three wells per test agent. A further set of three wells per bacterial strain was prepared and ciprofloxacin (128 μg·ml^−1^) was added to these as a positive control for killing. Ninety-six-well plates were incubated at 37°C for 20 h, and 10-μl aliquots from each well was spotted onto agar plates to assess the activity of each agent by the presence or absence of bacterial colonies following incubation of the agar plates.

### Ethical approval.

Breast milk was obtained from a healthy volunteer following ethical approval from the University of Warwick Biomedical and Scientific Research Ethics Committee (reference number REGO-2018-2329); informed consent was obtained prior to donation.

### Data availability.

Our final electronic database of remedies and ingredients in the *Lylye of Medicynes* is provided as Data Set S1. All codes and algorithms used in network analysis are available via C.I.D.G.’s website (https://charodelgenio.weebly.com).

10.1128/mBio.03136-19.2 TEXT S1 Recipe categories. Recipes from the *Lylye of Medicynes* identified as potentially worth exploration based on network analysis are provided. The criterion is simply the number of significant ingredient combinations that appeared together in a recipe. Within group lists, recipes are sorted alphabetically. Download Text S1, DOCX file, 0.02 MB. Copyright © 2020 Connelly et al. 2020 Connelly et al. This content is distributed under the terms of the Creative Commons Attribution 4.0 International license.
