# Supplementary material for: Data Mining a Medieval Medical Text Reveals Patterns in Ingredient Choice That Reflect Biological Activity against Infectious Agents
Source: mBio. 2020 Feb 11;11(1):e03136-19. doi: 10.1128/mBio.03136-19 (PMC7018648; doi:10.1128/mBio.03136-19)
Supplement: DATA SET S1 [file mBio.03136-19-sd001.pdf]

Middle English Ingredient Name	Chapter/Disease Name
vinegre	De Effimera_1
camomille	De Effimera_1
red rosis	De Effimera_1
sandali	De Effimera_1
water of rosis	De Effimera_1
water	De Effimera_1
sedes of whit papaver	De Effimera_2
oil of rosis	De Effimera_2
water of rosis	De Effimera_2
vinegre	De Effimera_2
juse of lactuca	De Effimera_2
portulaca	De Effimera_2
lentiginis	De Causone_1
water	De Causone_1
portulaca	De Causone_1
ungule caballine	De Causone_1
red rosis	De Causone_1
sandali	De Causone_1
muscatelini	De Causone_1
spodium	De Causone_1
floures of nenifar	De Causone_1
water of rosis	De Causone_1
wyn of pomegarnettes	De Causone_1
sugre	De Causone_1
floures of borage	De Causone_2
floures of violet	De Causone_2
cassiafistula	De Causone_2
tamarinde	De Causone_2
endive	De Terciana_1
scariol	De Terciana_1
lactuca recent	De Terciana_1
whit papaver	De Terciana_1

4 cold sedes major and minor	De Terciana_1
red rosis	De Terciana_1
nenifar	De Terciana_1
floures of violet	De Terciana_1
sandali	De Terciana_1
whit vinegre	De Terciana_1
sugre	De Terciana_1
floures of borage	De Terciana_2
floures of violet	De Terciana_2
prune	De Terciana_2
cassiafistula	De Terciana_2
tamarinde	De Terciana_2
oil of rosis	De Terciana_3
oil of nenifar	De Terciana_3
water of rosis	De Terciana_3
vinegre	De Terciana_3
ei whit	De Terciana_3
womman mylke	De Terciana_3
rote of fenel	De Quartana_1
petrosilie	De Quartana_1
bruscus	De Quartana_1
sparage	De Quartana_1
graminis	De Quartana_1
apium	De Quartana_1
middel rind of ebulus	De Quartana_1
middel rind of sambucus	De Quartana_1
fraxini	De Quartana_1
geneste	De Quartana_1
stronge vinegre	De Quartana_1
germandre	De Quartana_2
sticados	De Quartana_2
calamente	De Quartana_2
eupatorie	De Quartana_2

levys of absinthium	De Quartana_2
cicoree	De Quartana_2
endive	De Quartana_2
scariol	De Quartana_2
scolopendrie	De Quartana_2
4 cold sedes	De Quartana_2
lactuca	De Quartana_2
capparis	De Quartana_2
spicenardi	De Quartana_2
spice celtice	De Quartana_2
mel roset	De Quartana_2
sugre	De Quartana_2
fumiterre	De Quartana_2
borage	De Quartana_2
floures of borage	De Quartana_3
floures of violet	De Quartana_3
floures of sene	De Quartana_3
epithimi	De Quartana_3
liquirice	De Quartana_3
uva passe	De Quartana_3
gotys whay	De Quartana_3
rind of mirabolan indorum	De Quartana_3
rind of mirabolan kebulorum	De Quartana_3
rind of mirabolan emblicorum	De Quartana_3
diasene	De Quartana_4
diaborage	De Quartana_4
yerarufini	De Quartana_4
lapidis armenici nouies abluti	De Quartana_4
fuliginis	De Quartana_5
sal commune	De Quartana_5
urtica	De Quartana_5
vinegre	De Quartana_5
floures of borage	De Quartana_6

floures of violet	De Quartana_6
liquirice	De Quartana_6
epithimi	De Quartana_6
gotys whay	De Quartana_6
rind of mirabolan indorum	De Quartana_6
oxisacre	De Quartana_7
oximel diuretik	De Quartana_7
sirup of fumiterre	De Quartana_7
floures of borage	De Quartana_8
floures of violet	De Quartana_8
polipodi	De Quartana_8
anis	De Quartana_8
liquirice	De Quartana_8
uva passe	De Quartana_8
absinthium	De Quartana_8
agarik	De Quartana_8
lactuca	De Quartana_8
eupatorie	De Quartana_8
gotys whay	De Quartana_8
mirabolan indorum	De Quartana_8
rind of mirabolan	De Quartana_9
agarik	De Quartana_9
turbith	De Quartana_9
yerarufini	De Quartana_9
juse of mente	De Quartana_9
juse of eupatorie	De Quartana_9
juse of absinthium	De Quartana_9
anis	De Quartana_9
lacca	De Quartana_9
spicenardi	De Quartana_9
fenel	De Quartana_9
oximel squillitik	De Quartana_9
whit wyn	De Cotidiana_1

water	De Cotidiana_1
whit vinegre	De Cotidiana_1
rote of apium	De Cotidiana_1
oil of camomille	De Cotidiana_1
floures of borage	De Cotidiana_2
polipodi	De Cotidiana_2
turbith	De Cotidiana_2
agarik	De Cotidiana_2
anis	De Cotidiana_2
fenel	De Cotidiana_2
ameos	De Cotidiana_2
uva passe	De Cotidiana_2
liquirice	De Cotidiana_2
rind of mirabolan kebulorum	De Cotidiana_2
rote of fenel	De Cotidiana_3
petrosilie	De Cotidiana_3
apium	De Cotidiana_3
bruscus	De Cotidiana_3
sparage	De Cotidiana_3
sedes of endive	De Cotidiana_3
sedes of scariol	De Cotidiana_3
lacca	De Cotidiana_3
spicenardi	De Cotidiana_3
squinanti	De Cotidiana_3
mel roset	De Cotidiana_3
sugre	De Cotidiana_3
turbith	De Cotidiana_4
agarik	De Cotidiana_4
aloen	De Cotidiana_4
reubarbe	De Cotidiana_4
rind of mirabolan indorum	De Cotidiana_4
rind of mirabolan kebulorum	De Cotidiana_4
levys of mente	De Cotidiana_4

absinthium	De Cotidiana_4
eupatorie	De Cotidiana_4
zinziberis	De Cotidiana_4
lacca	De Cotidiana_4
anis	De Cotidiana_4
mastic	De Cotidiana_4
piperis	De Cotidiana_4
spicenardi	De Cotidiana_4
uva passe	De Cotidiana_4
liquirice	De Cotidiana_4
oximel squillitik	De Cotidiana_4
esula	De Cotidiana_4
diagridium	De Cotidiana_4
4 cold sedes major and minor	De Ethica_1
sedes of whit papaver	De Ethica_1
sedes of malve	De Ethica_1
pine	De Ethica_1
pistace	De Ethica_1
swete almaundes	De Ethica_1
jujube	De Ethica_1
sebesten	De Ethica_1
carica	De Ethica_1
uva passe	De Ethica_1
juse of liquirice	De Ethica_1
barlich	De Ethica_1
floures of nenifar	De Ethica_1
floures of violet	De Ethica_1
sugre	De Ethica_1
water	De Ethica_1
juse of borage	De Febribus Pestilencialibus_1
juse of buglosse	De Febribus Pestilencialibus_1
juse of melissa	De Febribus Pestilencialibus_1
juse of pomes swete	De Febribus Pestilencialibus_1

juse of acetose	De Febribus Pestilencialibus_1
juse of endive	De Febribus Pestilencialibus_1
juse of scariol	De Febribus Pestilencialibus_1
rosis	De Febribus Pestilencialibus_1
floures of nenifar	De Febribus Pestilencialibus_1
sandali	De Febribus Pestilencialibus_1
muscatelini	De Febribus Pestilencialibus_1
camphor	De Febribus Pestilencialibus_1
whit vinegre	De Febribus Pestilencialibus_1
water of rosis	De Febribus Pestilencialibus_1
sugre	De Febribus Pestilencialibus_1
sugre of rosis	De Febribus Pestilencialibus_2
triasandali	De Febribus Pestilencialibus_2
dragaganti frigidi	De Febribus Pestilencialibus_2
pannorum auri puri	De Febribus Pestilencialibus_2
margarite	De Febribus Pestilencialibus_2
jacincte	De Febribus Pestilencialibus_2
ossis de corde cervi	De Febribus Pestilencialibus_2
farina of faba	De Variolis_1
cicer	De Variolis_1
lentiginis	De Variolis_1
lupine	De Variolis_1
orobus	De Variolis_1
litarge	De Variolis_1
ceruse	De Variolis_1
aloen	De Variolis_1
litarge	De Variolis_2
ceruse lote	De Variolis_2
cathimia lote	De Variolis_2
poudre of canna	De Variolis_2
poudre of conchilia marina	De Variolis_2
oil	De Variolis_2
wex	De Variolis_2

juse of morelle	De Apostematibus Calidis Generatis Per Uiam Adustionis_1
lactuca	De Apostematibus Calidis Generatis Per Uiam Adustionis_1
portulaca	De Apostematibus Calidis Generatis Per Uiam Adustionis_1
virge pastoris	De Apostematibus Calidis Generatis Per Uiam Adustionis_1
lentiginis	De Apostematibus Calidis Generatis Per Uiam Adustionis_1
water of muscilage psillie	De Apostematibus Calidis Generatis Per Uiam Adustionis_1
barlich	De Apostematibus Calidis Generatis Per Uiam Adustionis_2
coriandre	De Apostematibus Calidis Generatis Per Uiam Adustionis_2
oil of violet	De Apostematibus Calidis Generatis Per Uiam Adustionis_2
wex	De Apostematibus Calidis Generatis Per Uiam Adustionis_2
bismalve	De Apostematibus Calidis Generatis Per Uiam Adustionis_2
farina of barlich	De Apostematibus Calidis Generatis Per Uiam Adustionis_2
oil	De Apostematibus Calidis Generatis Per Uiam Adustionis_2
hony	De Apostematibus Calidis Generatis Per Uiam Adustionis_2
juse of apium	De Apostematibus Calidis Generatis Per Uiam Adustionis_2
coriandre	De Apostematibus Calidis Generatis Per Uiam Adustionis_2
ciner of orobus	De Noli Me Tangere_1
litarge	De Noli Me Tangere_1
cathimia lote	De Noli Me Tangere_1
eris ust	De Noli Me Tangere_1
atrament	De Noli Me Tangere_1
tartar	De Noli Me Tangere_1
calx vive	De Noli Me Tangere_1
sal gemme	De Noli Me Tangere_1
rind of pomegarnettes	De Noli Me Tangere_1
ceruse lote	De Noli Me Tangere_1
aloen	De Noli Me Tangere_1
alum	De Noli Me Tangere_1
oil	De Noli Me Tangere_1
wex	De Noli Me Tangere_1
orobus	De Herpite Estiomens Cancro Seu Lupo_1
farina of faba	De Herpite Estiomens Cancro Seu Lupo_1
sal gemme	De Herpite Estiomens Cancro Seu Lupo_1

aristologie	De Herpite Estiomens Cancro Seu Lupo_1
mel roset	De Herpite Estiomens Cancro Seu Lupo_1
sirup of acetose	De Herpite Estiomens Cancro Seu Lupo_1
oil of rosis	De Herpite Estiomens Cancro Seu Lupo_2
whit wex	De Herpite Estiomens Cancro Seu Lupo_2
juse of lupine	De Herpite Estiomens Cancro Seu Lupo_2
ceruse	De Herpite Estiomens Cancro Seu Lupo_2
plumbum ust, lote	De Herpite Estiomens Cancro Seu Lupo_2
pamfiligos	De Herpite Estiomens Cancro Seu Lupo_2
olibanum	De Herpite Estiomens Cancro Seu Lupo_2
wex	De Herpite Estiomens Cancro Seu Lupo_2
oil	De Herpite Estiomens Cancro Seu Lupo_2
rote of aristologie	De Herpite Estiomens Cancro Seu Lupo_3
alum	De Herpite Estiomens Cancro Seu Lupo_3
floris eris	De Herpite Estiomens Cancro Seu Lupo_3
vitriol	De Herpite Estiomens Cancro Seu Lupo_3
galle	De Herpite Estiomens Cancro Seu Lupo_3
eris ust	De Herpite Estiomens Cancro Seu Lupo_3
ceruse lote	De Herpite Estiomens Cancro Seu Lupo_3
aloen	De Herpite Estiomens Cancro Seu Lupo_3
juse of cucumeris asinini	De Herpite Estiomens Cancro Seu Lupo_3
hony	De Herpite Estiomens Cancro Seu Lupo_3
vinegre	De Herpite Estiomens Cancro Seu Lupo_3
antimonie	De Herpite Estiomens Cancro Seu Lupo_4
eris ust	De Herpite Estiomens Cancro Seu Lupo_4
litarge	De Herpite Estiomens Cancro Seu Lupo_4
cathimia	De Herpite Estiomens Cancro Seu Lupo_4
argent vive	De Herpite Estiomens Cancro Seu Lupo_4
marcasite	De Herpite Estiomens Cancro Seu Lupo_4
ceruse lote	De Herpite Estiomens Cancro Seu Lupo_4
balaustia	De Herpite Estiomens Cancro Seu Lupo_4
alum	De Herpite Estiomens Cancro Seu Lupo_4
sarcocolla	De Herpite Estiomens Cancro Seu Lupo_4

rind of olibanum	De Herpite Estiomens Cancro Seu Lupo_4
pamfiligos	De Herpite Estiomens Cancro Seu Lupo_4
olde oil	De Herpite Estiomens Cancro Seu Lupo_4
wex	De Herpite Estiomens Cancro Seu Lupo_4
galle	De Herpite Estiomens Cancro Seu Lupo_5
rind of pomegarnettes	De Herpite Estiomens Cancro Seu Lupo_5
sanguis draconis	De Herpite Estiomens Cancro Seu Lupo_5
ceruse	De Herpite Estiomens Cancro Seu Lupo_5
aloen	De Herpite Estiomens Cancro Seu Lupo_5
sarcocolla	De Herpite Estiomens Cancro Seu Lupo_5
litarge	De Herpite Estiomens Cancro Seu Lupo_5
cathimia lote	De Herpite Estiomens Cancro Seu Lupo_5
alum	De Herpite Estiomens Cancro Seu Lupo_5
eris ust, ablute	De Herpite Estiomens Cancro Seu Lupo_5
mirre	De Herpite Estiomens Cancro Seu Lupo_5
rind of olibanum	De Herpite Estiomens Cancro Seu Lupo_5
oil of rosis	De Herpite Estiomens Cancro Seu Lupo_5
pamfiligos	De Herpite Estiomens Cancro Seu Lupo_5
farina of ris	De Inpetigine et Serpigine_1
milie	De Inpetigine et Serpigine_1
barlich	De Inpetigine et Serpigine_1
faba	De Inpetigine et Serpigine_1
lupine	De Inpetigine et Serpigine_1
cicer	De Inpetigine et Serpigine_1
galle	De Inpetigine et Serpigine_1
mirabolan	De Inpetigine et Serpigine_1
hony	De Inpetigine et Serpigine_1
vinegre	De Inpetigine et Serpigine_1
juse of bleta	De Inpetigine et Serpigine_1
nitrum	De Inpetigine et Serpigine_2
litarge	De Inpetigine et Serpigine_2
piretre	De Inpetigine et Serpigine_2
levys of agnus castus	De Inpetigine et Serpigine_2

eris ust	De Inpetigine et Serpigine_2
vitriol	De Inpetigine et Serpigine_2
sulfur vive	De Inpetigine et Serpigine_2
vinegre	De Inpetigine et Serpigine_2
anacardus	De Inpetigine et Serpigine_3
cost	De Inpetigine et Serpigine_3
ellebore nigrum	De Inpetigine et Serpigine_3
atrament	De Inpetigine et Serpigine_3
aristologie combust	De Inpetigine et Serpigine_3
water	De Inpetigine et Serpigine_3
oil	De Inpetigine et Serpigine_3
wex	De Inpetigine et Serpigine_3
sulfur vive	De Inpetigine et Serpigine_4
auripigment red	De Inpetigine et Serpigine_4
juse of fumiterre	De Inpetigine et Serpigine_4
red wyn	De Inpetigine et Serpigine_4
unguentum citrinum	De Inpetigine et Serpigine_4
hoot water	De Inpetigine et Serpigine_4
bran	De Inpetigine et Serpigine_4
ungentum fuscum	De Inpetigine et Serpigine_5
unguentum citrinum	De Inpetigine et Serpigine_5
litarge	De Inpetigine et Serpigine_5
zinziberis	De Fistula_1
viridis eris	De Fistula_1
aloen	De Fistula_1
vinegre	De Fistula_1
hony	De Fistula_1
sal armoniac	De Fistula_2
vitriol	De Fistula_2
auripigment red	De Fistula_2
auripigment citrine	De Fistula_2
viridis eris	De Fistula_2
levys of ruta	De Fistula_3

celidonie	De Fistula_3
agrimonie	De Fistula_3
whit wyn	De Fistula_3
agrimonie	De Fistula_4
levys of olive	De Fistula_4
ceterac	De Fistula_4
whit wyn	De Fistula_4
rote of ellebore	De Fistula_5
juse of fumiterre	De Lepra_1
juse of borage	De Lepra_1
juse of scabiose	De Lepra_1
juse of lappacii acuti	De Lepra_1
floures of borage	De Lepra_1
floures of violet	De Lepra_1
floures of sene	De Lepra_1
epithimi	De Lepra_1
cuscute	De Lepra_1
polipodi	De Lepra_1
anete	De Lepra_1
liquirice	De Lepra_1
mel roset	De Lepra_1
sugre	De Lepra_1
diambra	De Lepra_2_a
diacitoniten	De Lepra_2_a
julii	De Lepra_2_a
diamargariton	De Lepra_2_a
dianthos	De Lepra_2_b
dianison	De Lepra_2_b
sugre of rosis	De Lepra_2_c
triasandali	De Lepra_2_c
floures of borage	De Lepra_3
floures of violet	De Lepra_3
levys of sene	De Lepra_3

epithimi	De Lepra_3
polipodi	De Lepra_3
anis	De Lepra_3
uva passe	De Lepra_3
liquirice	De Lepra_3
gotys whay	De Lepra_3
rind of mirabolan indorum	De Lepra_3
rind of mirabolan belliricorum	De Lepra_3
rind of mirabolan emblicorum	De Lepra_3
electuari de succo rosarum	De Lepra_4
oxilaxativum	De Lepra_4
yerarufini	De Lepra_4
pulpe coloquintide	De Lepra_4
bdellium	De Lepra_4
yerapigra	De Lepra_5
yerarufini	De Lepra_5
esula	De Lepra_5
mastic	De Lepra_5
diasene	De Lepra_6
diaborage	De Lepra_6
yerarufini	De Lepra_6
pulpe coloquintide	De Lepra_6
bdellium	De Lepra_6
poudre of epithimi	De Lepra_7
gotys whay	De Lepra_7
celidonie	De Lepra_8
tapsia	De Lepra_8
nasturcium	De Lepra_8
agnus castus	De Lepra_8
pulegie regale	De Lepra_8
origane	De Lepra_8
macropiperis	De Lepra_8
nux muscata	De Lepra_8

whit wyn	De Lepra_8
oil of sisamie	De Lepra_8
euforbia	De Lepra_9
piperis	De Lepra_9
castor	De Lepra_9
sinapis	De Lepra_9
oximel squillitik	De Lepra_9
wex	De Lepra_9
fumiterre	De Lepra_10
lappacii acuti	De Lepra_10
anthos	De Lepra_10
floures of sticados	De Lepra_10
camomille	De Lepra_10
mellilote	De Lepra_10
piretre	De Lepra_11_a
stafisagre	De Lepra_11_a
euforbia	De Lepra_11_a
macropiperis	De Lepra_11_a
nux muscata	De Lepra_11_a
sulfur vive	De Lepra_11_a
auripigment red	De Lepra_11_a
sinapis	De Lepra_11_a
aloen	De Lepra_11_a
hony	De Lepra_11_a
vinegre	De Lepra_11_a
hoot water	De Lepra_11_b
triacle	De Lepra_11_c
fumiterre	De Lepra_11_c
wyn	De Lepra_11_c
rote of lilie	De Lepra_12
millefolii	De Lepra_12
draguncia	De Lepra_12
farina of faba	De Lepra_12

water of rosis	De Lepra_12
gariofilum	De Lepra_12
nux muscata	De Lepra_12
unguentum citrinum	De Lepra_13
unguentum ad scabiem	De Lepra_13
ungentum fuscum	De Lepra_13
litarge	De Lepra_13
auripigment red	De Morphea_1
juse of fumiterre	De Morphea_1
sirup of fumiterre	De Morphea_1
floures of borage	De Scabie_1
floures of violet	De Scabie_1
floures of sene	De Scabie_1
epithimi	De Scabie_1
liquirice	De Scabie_1
uva passe	De Scabie_1
agarik	De Scabie_1
turbith	De Scabie_1
zinziberis	De Scabie_1
gotys whay	De Scabie_1
rind of mirabolan kebulorum	De Scabie_1
rind of mirabolan indorum	De Scabie_1
aloen	De Scabie_2
agarik	De Scabie_2
turbith	De Scabie_2
pulpe coloquintide	De Scabie_2
levys of mente	De Scabie_2
levys of absinthium	De Scabie_2
zinziberis	De Scabie_2
mastic	De Scabie_2
bdellium	De Scabie_2
oximel squillitik	De Scabie_2
yerapigra	De Scabie_3

yerarufini	De Scabie_3
esula	De Scabie_3
mastic	De Scabie_3
diaborage	De Scabie_4
diasene	De Scabie_4
yerarufini	De Scabie_4
lapidis armenici nouies abluti	De Scabie_4
floures of borage	De Scabie_5
floures of violet	De Scabie_5
nenifar	De Scabie_5
epithimi	De Scabie_5
camomille	De Scabie_5
mellilote	De Scabie_5
fumiterre	De Scabie_5
unguentum citrinum	De Scabie_6
litarge	De Scabie_6
sulfur vive	De Scabie_6
sirup of fumiterre	De Scabie_6
unguentum citrinum	De Scabie_7
ungentum fuscum	De Scabie_7
unguentum ad scabiem	De Scabie_7
calx extinct	De Scabie_7
sulfur	De Scabie_7
litarge	De Scabie_7
sirup of fumiterre	De Scabie_7
rote of enule campane	De Scabie_8
swynes grece recent	De Scabie_8
oil of rosis	De Scabie_9
fuliginis	De Scabie_9
oil of notys	De Scabie_9
vinegre	De Scabie_9
wex	De Scabie_9
antimonie	De Scabie_10

eris ust	De Scabie_10
litarge	De Scabie_10
cathimia argent	De Scabie_10
marcasite	De Scabie_10
ceruse	De Scabie_10
balaustia	De Scabie_10
alum	De Scabie_10
sarcocolla	De Scabie_10
rind of olibanum	De Scabie_10
pamfiligos	De Scabie_10
olde oil	De Scabie_10
wex	De Scabie_10
rote of whit ellebore recent	De Scabie_11
swynes grece recent	De Scabie_11
juse of apium	De Scabie Sicca cum Ulceribus_1
juse of levys of lilie	De Scabie Sicca cum Ulceribus_1
juse of whit papaver	De Scabie Sicca cum Ulceribus_1
water of rosis	De Scabie Sicca cum Ulceribus_1
whit vinegre	De Scabie Sicca cum Ulceribus_1
aloen	De Scabie Sicca cum Ulceribus_1
oil of sisamie	De Scabie Sicca cum Ulceribus_1
wex	De Scabie Sicca cum Ulceribus_1
dragaganti	De Scabie Sicca cum Ulceribus_2
gummi arabic	De Scabie Sicca cum Ulceribus_2
sedes of malve	De Scabie Sicca cum Ulceribus_2
bismalve	De Scabie Sicca cum Ulceribus_2
aloen	De Scabie Sicca cum Ulceribus_2
sarcocolla	De Scabie Sicca cum Ulceribus_2
swynes grece recent	De Scabie Sicca cum Ulceribus_2
capoun grece	De Scabie Sicca cum Ulceribus_2
oil of swete almaundes	De Scabie Sicca cum Ulceribus_2
wex	De Scabie Sicca cum Ulceribus_2
rote of fenel	De Malo Mortuo_1

petrosilie	De Malo Mortuo_1
bruscus	De Malo Mortuo_1
sparage	De Malo Mortuo_1
graminis	De Malo Mortuo_1
rind of capparis	De Malo Mortuo_1
fraxini	De Malo Mortuo_1
tamarisci	De Malo Mortuo_1
whit vinegre	De Malo Mortuo_1
borage	De Malo Mortuo_2
scabiose	De Malo Mortuo_2
fumiterre	De Malo Mortuo_2
lappacii acuti	De Malo Mortuo_2
buglosse	De Malo Mortuo_2
melissa	De Malo Mortuo_2
sticados	De Malo Mortuo_2
levys of sene	De Malo Mortuo_2
epithimi	De Malo Mortuo_2
polipodi	De Malo Mortuo_2
anthos	De Malo Mortuo_2
floures of borage	De Malo Mortuo_2
floures of violet	De Malo Mortuo_2
anis	De Malo Mortuo_2
mel roset	De Malo Mortuo_2
sugre	De Malo Mortuo_2
juse of raphane	De Malo Mortuo_3
spatule fetide	De Malo Mortuo_3
aristologie	De Malo Mortuo_3
cucumeris asinini	De Malo Mortuo_3
borage	De Malo Mortuo_3
fumiterre	De Malo Mortuo_3
scabiose	De Malo Mortuo_3
lappacii acuti	De Malo Mortuo_3
ellebore nigrum	De Malo Mortuo_3

ellebore whit	De Malo Mortuo_3
calx vive	De Malo Mortuo_3
sulfur	De Malo Mortuo_3
argent vive extinct	De Malo Mortuo_3
oil of notys	De Malo Mortuo_3
wex	De Malo Mortuo_3
ungentum fuscum	De Pustulis_1
unguentum ad scabiem	De Pustulis_1
litarge	De Pustulis_1
tartar	De Pustulis_1
ellebore whit	De Pustulis_1
ellebore nigrum	De Pustulis_1
argent vive extinct	De Pustulis_1
oil	De Pustulis_1
vinegre	De Pustulis_1
juse of fumiterre	De Pustulis_2
celidonie	De Pustulis_2
scabiose	De Pustulis_2
lappacii acuti	De Pustulis_2
litarge	De Pustulis_2
ceruse lote	De Pustulis_2
eris ust	De Pustulis_2
vinegre	De Pustulis_2
oil	De Pustulis_2
olde grece	De Pustulis_2
litarge	De Vulneribus_1
ceruse lote	De Vulneribus_1
cathimia lote	De Vulneribus_1
eris ust, ablute	De Vulneribus_1
aloen	De Vulneribus_1
sarcocolla	De Vulneribus_1
rote of aristologie	De Vulneribus_1
tartar	De Vulneribus_1

wyn	De Vulneribus_1
hony	De Vulneribus_1
wex	De Vulneribus_1
oil	De Vulneribus_1
aloen	De Vulneribus_2
olibanum	De Vulneribus_2
pilorum leporis	De Vulneribus_2
sanguis draconis	De Vulneribus_2
bole armoniac	De Vulneribus_2
ei whit	De Vulneribus_2
agrimonie	De Vulneribus_3
pimpinelle	De Vulneribus_3
betonice	De Vulneribus_3
verbene	De Vulneribus_3
centorie major	De Vulneribus_3
sticados	De Vulneribus_3
salvie	De Vulneribus_3
pilocelle	De Vulneribus_3
consolida major	De Vulneribus_3
consolida media	De Vulneribus_3
consolida minor	De Vulneribus_3
plantago	De Vulneribus_3
sanamunde	De Vulneribus_3
rubee majoris	De Vulneribus_3
red caul	De Vulneribus_3
stercus columbinum	De Vulneribus_3
galle	De Vulneribus_3
absinthium	De Vulneribus_3
tansie	De Vulneribus_3
lingue avis	De Vulneribus_3
edereterestis	De Vulneribus_3
fragarie	De Vulneribus_3
buglosse	De Vulneribus_3

genciane	De Vulneribus_3
whit wyn	De Vulneribus_3
hony	De Vulneribus_3
picis liquide	De Vulneribus_4
wex	De Vulneribus_4
terebintine	De Vulneribus_4
vinegre	De Vulneribus_4
kyne mylke	De Vulneribus_4
womman mylke	De Vulneribus_4
juse of betonice	De Vulneribus_4
juse of centorie major	De Vulneribus_4
terebintine	De Vulneribus_5
whit wex	De Vulneribus_5
vinegre	De Vulneribus_5
juse of betonice	De Vulneribus_5
juse of verbene	De Vulneribus_5
millefolii	De Vulneribus_5
kyne mylke	De Vulneribus_5
womman mylke	De Vulneribus_5
juse of pomegarnettes	De Iter Agentibus_1
juse of acetose	De Iter Agentibus_1
whit papaver	De Iter Agentibus_1
juse of portulaca	De Iter Agentibus_1
sugre	De Iter Agentibus_1
cold water	De Iter Agentibus_1
dragaganti frigidi	De Iter Agentibus_2
diapapaver	De Iter Agentibus_2
sugre of rosis	De Iter Agentibus_2
violet	De Iter Agentibus_2
triasandali	De Iter Agentibus_2
4 cold sedes	De Iter Agentibus_3
sedes of whit papaver	De Iter Agentibus_3
dragaganti frigidi	De Iter Agentibus_3

juse of liquirice	De Iter Agentibus_3
sedes of portulaca	De Iter Agentibus_3
sirup of nenifar	De Iter Agentibus_3
diatrion pipereon	De Iter Agentibus_4
diamente	De Iter Agentibus_4
dianthos cum musco	De Iter Agentibus_4
crocus cum musco	De Iter Agentibus_4
ambra grisie	De Iter Agentibus_4
zinziberis	De Iter Agentibus_5
cinamome	De Iter Agentibus_5
gariofilum	De Iter Agentibus_5
nux muscata	De Iter Agentibus_5
spicenardi	De Iter Agentibus_5
sugre	De Iter Agentibus_5
levys of sene	De Iter Agentibus_5
epithimi	De Iter Agentibus_5
polipodi	De Iter Agentibus_5
aloen	De Canicie_1
agarik	De Canicie_1
turbith	De Canicie_1
pulpe coloquintide	De Canicie_1
mastic	De Canicie_1
bdellium	De Canicie_1
zinziberis	De Canicie_1
anis	De Canicie_1
cinamome	De Canicie_1
levys of absinthium	De Canicie_1
levys of mente	De Canicie_1
oximel squillitik	De Canicie_1
floures of borage	De Dolore Capitis _1
floures of violet	De Dolore Capitis _1
uva passe	De Dolore Capitis _1
gotys whay	De Dolore Capitis _1

rind of mirabolan indorum	De Dolore Capitis _1
cassiafistula	De Dolore Capitis _1
mastic	De Dolore Capitis _1
diagridium	De Dolore Capitis _1
floures of borage	De Dolore Capitis _2
floures of violet	De Dolore Capitis _2
levys of sene	De Dolore Capitis _2
polipodi	De Dolore Capitis _2
epithimi	De Dolore Capitis _2
anis	De Dolore Capitis _2
uva passe	De Dolore Capitis _2
liquirice	De Dolore Capitis _2
gotys whay	De Dolore Capitis _2
rind of mirabolan indorum gummosum	De Dolore Capitis _2
rind of mirabolan belliricorum gummosum	De Dolore Capitis _2
rind of mirabolan emblicorum gummosum	De Dolore Capitis _2
aloen	De Dolore Capitis _3
agarik	De Dolore Capitis _3
turbith	De Dolore Capitis _3
pulpe coloquintide	De Dolore Capitis _3
levys of absinthium	De Dolore Capitis _3
levys of mente	De Dolore Capitis _3
mastic	De Dolore Capitis _3
anis	De Dolore Capitis _3
cinamome	De Dolore Capitis _3
bdellium	De Dolore Capitis _3
oximel squillitik	De Dolore Capitis _3
floures of borage	De Dolore Capitis _4
floures of violet	De Dolore Capitis _4
turbith	De Dolore Capitis _4
anis	De Dolore Capitis _4
polipodi	De Dolore Capitis _4
uva passe	De Dolore Capitis _4

liquirice	De Dolore Capitis _4
salvie	De Litargia Vera_1
mente	De Litargia Vera_1
ruta	De Litargia Vera_1
calamente	De Litargia Vera_1
betonice	De Litargia Vera_1
anis	De Litargia Vera_1
fenel	De Litargia Vera_1
comin	De Litargia Vera_1
whit vinegre	De Litargia Vera_1
sugre	De Litargia Vera_1
hony	De Litargia Vera_1
capilli veneris	De Litargia Vera_1
bruscus	De Litargia Vera_1
sparage	De Litargia Vera_1
yerapigra	De Litargia Vera_1
water	De Litargia Vera_1
yerapigra	De Litargia Vera_2
pulpe coloquintide	De Litargia Vera_2
bdellium	De Litargia Vera_2
aloen	De Litargia Vera_3
agarik	De Litargia Vera_3
turbith	De Litargia Vera_3
pulpe coloquintide	De Litargia Vera_3
bdellium	De Litargia Vera_3
anis	De Litargia Vera_3
cinamome	De Litargia Vera_3
mastic	De Litargia Vera_3
zinziberis	De Litargia Vera_3
levys of mente	De Litargia Vera_3
levys of absinthium	De Litargia Vera_3
oximel squillitik	De Litargia Vera_3
acorus	De Corupcione Memorie_1

cipresse	De Corupcione Memorie_1
yreos	De Corupcione Memorie_1
aristologie	De Corupcione Memorie_1
calamente	De Corupcione Memorie_1
nasturcium	De Corupcione Memorie_1
salvie	De Corupcione Memorie_1
centorie	De Corupcione Memorie_1
fenel	De Corupcione Memorie_1
ameos	De Corupcione Memorie_1
siseleos	De Corupcione Memorie_1
hony	De Corupcione Memorie_1
whit vinegre	De Corupcione Memorie_1
yerapigra	De Corupcione Memorie_2
pulpe coloquintide	De Corupcione Memorie_2
bdellium	De Corupcione Memorie_2
aurea alexanadrina	De Corupcione Memorie_2
tyriaca	De Corupcione Memorie_2
diatrion pipereon	De Corupcione Memorie_3
acorus	De Corupcione Memorie_3
ciperus	De Corupcione Memorie_3
mirre	De Corupcione Memorie_3
crocus	De Corupcione Memorie_3
sugre	De Corupcione Memorie_3
yeralogodion	De Corupcione Memorie_4
theodoricon	De Corupcione Memorie_4
anacardus	De Corupcione Memorie_4
acorus	De Corupcione Memorie_5
rote of ciperus	De Corupcione Memorie_5
spicenardi	De Corupcione Memorie_5
piperis	De Corupcione Memorie_5
piperis long	De Corupcione Memorie_5
anis	De Corupcione Memorie_5
comin	De Corupcione Memorie_5

cinamome	De Corupcione Memorie_5
nux muscata	De Corupcione Memorie_5
gariofilum	De Corupcione Memorie_5
cardamome	De Corupcione Memorie_5
brothe of cicer	De Corupcione Memorie_5
yeralogodion	De Corupcione Memorie_6
theodoricon	De Corupcione Memorie_6
anacardus	De Corupcione Memorie_6
piperis	De Corupcione Memorie_7
macropiperis	De Corupcione Memorie_7
mirabolan kebulorum	De Corupcione Memorie_7
mirabolan emblicorum	De Corupcione Memorie_7
mirabolan belliricorum	De Corupcione Memorie_7
mirabolan indorum	De Corupcione Memorie_7
cost	De Corupcione Memorie_7
anacardus	De Corupcione Memorie_7
zinziberis	De Corupcione Memorie_7
butere	De Corupcione Memorie_7
laurel	De Corupcione Memorie_7
ciperus	De Corupcione Memorie_7
hony	De Corupcione Memorie_7
water of fenel	De Corupcione Memorie_7
water of apium	De Corupcione Memorie_7
dianthos cum musco	De Sompno Innaturali_1
potio muscata	De Sompno Innaturali_1
diacodion	De Vigiliis_1
diapapaver	De Vigiliis_1
endive	De Mania et Malencolia_1
scariol	De Mania et Malencolia_1
lactuca	De Mania et Malencolia_1
juse of borage	De Mania et Malencolia_1
juse of fumiterre	De Mania et Malencolia_1
juse of buglosse	De Mania et Malencolia_1

juse of pomes swete	De Mania et Malencolia_1
vinegre squillitik	De Mania et Malencolia_1
mirabolan indorum	De Mania et Malencolia_1
thime	De Mania et Malencolia_1
levys of sene	De Mania et Malencolia_1
water of chese	De Mania et Malencolia_1
sugre	De Mania et Malencolia_1
rote of fenel	De Mania et Malencolia_2_a
petrosilie	De Mania et Malencolia_2_a
bruscus	De Mania et Malencolia_2_a
sparage	De Mania et Malencolia_2_a
graminis	De Mania et Malencolia_2_a
juse of borage	De Mania et Malencolia_2_a
juse of scabiose	De Mania et Malencolia_2_a
juse of lappacii acuti	De Mania et Malencolia_2_a
juse of fumiterre	De Mania et Malencolia_2_a
juse of buglosse	De Mania et Malencolia_2_a
juse of melissa	De Mania et Malencolia_2_a
whit vinegre	De Mania et Malencolia_2_a
levys of sene	De Mania et Malencolia_2_a
epithimi	De Mania et Malencolia_2_a
mel roset	De Mania et Malencolia_2_a
mirabolan indorum	De Mania et Malencolia_2_b
mirabolan belliricorum	De Mania et Malencolia_2_b
mirabolan emblicorum	De Mania et Malencolia_2_b
levys of sene	De Mania et Malencolia_2_b
lapidis armenici	De Mania et Malencolia_2_b
lapidis lazuli	De Mania et Malencolia_2_b
rote of apium	De Mania et Malencolia_3_a
bruscus	De Mania et Malencolia_3_a
sparage	De Mania et Malencolia_3_a
graminis	De Mania et Malencolia_3_a
scolopendrie	De Mania et Malencolia_3_a

anis	De Mania et Malencolia_3_a
fenel	De Mania et Malencolia_3_a
spicenardi	De Mania et Malencolia_3_a
squinanti	De Mania et Malencolia_3_a
hony	De Mania et Malencolia_3_a
mirabolan kebulorum	De Mania et Malencolia_3_b
mirabolan indorum	De Mania et Malencolia_3_b
polipodi	De Mania et Malencolia_3_b
agarik	De Mania et Malencolia_3_b
diacameron	De Mania et Malencolia_4
diarodon abbatis	De Mania et Malencolia_4
julii	De Mania et Malencolia_4
diamargariton	De Mania et Malencolia_4
diambra	De Mania et Malencolia_4
leticia galen	De Mania et Malencolia_4
pliris cum musco	De Mania et Malencolia_4
dianthos	De Mania et Malencolia_4
juse of lactuca	De Frenesi_1
juse of cucurbite	De Frenesi_1
juse of portulaca	De Frenesi_1
lentiginis	De Frenesi_1
water	De Frenesi_1
4 cold sedes	De Frenesi_1
sedes of whit papaver	De Frenesi_1
wyn of pomegarnettes	De Frenesi_1
sandali	De Frenesi_1
sugre	De Frenesi_1
floures of borage	De Frenesi_2
floures of violet	De Frenesi_2
prune	De Frenesi_2
cassiafistula	De Frenesi_2
tamarinde	De Frenesi_2
diadragagantum	De Frenesi_3

water of rosis	De Frenesi_3
muscilage of psillie	De Frenesi_3
wyn of pomegarnettes	De Frenesi_3
farina of whit papaver	De Frenesi_3
oil of rosis	De Frenesi_3
vinegre	De Frenesi_3
wex	De Frenesi_3
endive	De Epilencia_1
scariol	De Epilencia_1
lactuca	De Epilencia_1
coriandre recent	De Epilencia_1
ruta	De Epilencia_1
pionie	De Epilencia_1
ysope	De Epilencia_1
poli montani	De Epilencia_1
absinthium	De Epilencia_1
juse of fumiterre	De Epilencia_1
sedes of ruta	De Epilencia_1
sedes of pionie	De Epilencia_1
siseleos	De Epilencia_1
anis	De Epilencia_1
fenel	De Epilencia_1
cold sedes	De Epilencia_1
hony	De Epilencia_1
sugre	De Epilencia_1
agarik	De Epilencia_2
pulpe coloquintide	De Epilencia_2
diagridium	De Epilencia_2
reubarbe	De Epilencia_2
mastic	De Epilencia_2
bdellium	De Epilencia_2
sedes of ruta	De Epilencia_2
siseleos	De Epilencia_2

gariofilum	De Epilencia_2
levys of absinthium	De Epilencia_2
levys of mente	De Epilencia_2
yerarufini	De Epilencia_2
oximel squillitik	De Epilencia_2
tyriaca	De Epilencia_3
rubee trociscata	De Epilencia_3
absinthium	De Epilencia_3
wyn	De Epilencia_3
rote of yreos	De Epilencia_4
aristologie	De Epilencia_4
rosis	De Epilencia_4
squille	De Epilencia_4
ysope	De Epilencia_4
pionie romane	De Epilencia_4
visci quercini	De Epilencia_4
poli montani	De Epilencia_4
origane	De Epilencia_4
calamente	De Epilencia_4
salvie	De Epilencia_4
ruta	De Epilencia_4
sedes of ruta	De Epilencia_4
siseleos	De Epilencia_4
sedes of pionie romane	De Epilencia_4
anis	De Epilencia_4
fenel	De Epilencia_4
floures of sticados arabici	De Epilencia_4
juse of absinthium	De Epilencia_4
fumiterre	De Epilencia_4
hony	De Epilencia_4
agarik	De Epilencia_5_a
turbith	De Epilencia_5_a
aloen	De Epilencia_5_a

euforbia	De Epilencia_5_a
pulpe coloquintide	De Epilencia_5_a
esula	De Epilencia_5_a
yeralogodion	De Epilencia_5_a
mastic	De Epilencia_5_a
bdellium	De Epilencia_5_a
siseleos	De Epilencia_5_a
sedes of ruta	De Epilencia_5_a
zinziberis	De Epilencia_5_a
levys of mente	De Epilencia_5_a
sedes of absinthium	De Epilencia_5_a
gariofilum	De Epilencia_5_a
spicenardi	De Epilencia_5_a
oximel squillitik	De Epilencia_5_a
tyriaca	De Epilencia_5_b
mithridatum	De Epilencia_5_b
rote of fenel	De Epilencia_6
petrosilie	De Epilencia_6
bruscus	De Epilencia_6
sparage	De Epilencia_6
graminis	De Epilencia_6
aristologie	De Epilencia_6
ysope	De Epilencia_6
pionie romane	De Epilencia_6
ruta	De Epilencia_6
poli montani	De Epilencia_6
juse of borage	De Epilencia_6
juse of scabiose	De Epilencia_6
juse of fumiterre	De Epilencia_6
sedes of pionie	De Epilencia_6
sedes of ruta	De Epilencia_6
visci quercini	De Epilencia_6
epithimi	De Epilencia_6

levys of sene	De Epilencia_6
polipodi	De Epilencia_6
anis	De Epilencia_6
floures of sticados arabici	De Epilencia_6
mel roset	De Epilencia_6
agarik	De Epilencia_7_a
pulpe coloquintide	De Epilencia_7_a
lapidis armenici nouies abluti	De Epilencia_7_a
yerarufini	De Epilencia_7_a
bdellium	De Epilencia_7_a
mastic	De Epilencia_7_a
sedes of ruta	De Epilencia_7_a
siseleos	De Epilencia_7_a
cinamome	De Epilencia_7_a
gariofilum	De Epilencia_7_a
levys of mente	De Epilencia_7_a
levys of absinthium	De Epilencia_7_a
oximel squillitik	De Epilencia_7_a
aurea alexanadrina	De Epilencia_7_b
triacle	De Epilencia_7_b
diambra	De Epilencia_8
diacameron	De Epilencia_8
diamargariton	De Epilencia_8
diamente	De Epilencia_9
diatrion pipereon	De Epilencia_9
diacitoniten	De Epilencia_9
triasandali	De Epilencia_10
sugre of rosis	De Epilencia_10
diarodon abbatis	De Epilencia_10
diatrion pipereon	De Epilencia_11
ysope	De Epilencia_11
mente drie	De Epilencia_11
gariofilum	De Epilencia_11

cinamome	De Epilencia_11
cubebe	De Epilencia_11
nux muscata	De Epilencia_11
cardamome	De Epilencia_11
ciperus	De Epilencia_11
zedoaria	De Epilencia_11
sedes of ruta	De Epilencia_11
sedes of pionie	De Epilencia_11
siseleos	De Epilencia_11
anis	De Epilencia_11
fenel	De Epilencia_11
comin	De Epilencia_11
ambra grisie	De Epilencia_12
storace calamite	De Epilencia_12
lapdanum	De Epilencia_12
mastic	De Epilencia_12
olibanum	De Epilencia_12
gariofilum	De Epilencia_12
lignum aloes	De Epilencia_12
water of rosis	De Epilencia_12
musco	De Epilencia_12
ambra grisie	De Epilencia_13
sedes of pionie	De Epilencia_13
ysope	De Epilencia_13
blatta bizancie	De Epilencia_13
sandali	De Epilencia_13
muscatelini	De Epilencia_13
coriandre	De Epilencia_13
water of rosis	De Epilencia_13
vinegre	De Epilencia_13
lapdanum	De Epilencia_13
ellebore whit	De Apoplexia_1
piperis	De Apoplexia_1

castor	De Apoplexia_1
calamente	De Apoplexia_2
mente	De Apoplexia_2
ruta	De Apoplexia_2
wyn	De Apoplexia_2
euforbia	De Apoplexia_3
piretre	De Apoplexia_3
castor	De Apoplexia_3
galbanum	De Apoplexia_3
serapinum	De Apoplexia_3
opoponac	De Apoplexia_3
diatrion pipereon	De Apoplexia_3
sulfur vive	De Apoplexia_3
sinapis	De Apoplexia_3
leonis grece	De Apoplexia_3
malve	De Paralisi_1
mercurial	De Paralisi_1
abrotane	De Paralisi_1
centorie	De Paralisi_1
sticados	De Paralisi_1
ruta	De Paralisi_1
juse of bleta	De Paralisi_1
oil of comin	De Paralisi_1
hony	De Paralisi_1
sal	De Paralisi_1
furfur	De Paralisi_1
yerapigra	De Paralisi_1
yeralogodion	De Paralisi_1
rote of fenel	De Paralisi_2
petrosilie	De Paralisi_2
apium	De Paralisi_2
genciane	De Paralisi_2
aristologie	De Paralisi_2

rosis	De Paralisi_2
capparis	De Paralisi_2
squille	De Paralisi_2
acorus	De Paralisi_2
piretre	De Paralisi_2
raphane	De Paralisi_2
lilie	De Paralisi_2
whit wyn	De Paralisi_2
narcissus	De Paralisi_3
salvie	De Paralisi_3
ruta	De Paralisi_3
calamente	De Paralisi_3
centorie minor	De Paralisi_3
cost	De Paralisi_3
ysope	De Paralisi_3
anis	De Paralisi_3
fenel	De Paralisi_3
carvi agreste	De Paralisi_3
sedes of ruta	De Paralisi_3
spicenardi	De Paralisi_3
squinanti	De Paralisi_3
hony	De Paralisi_3
agarik	De Paralisi_4
turbith	De Paralisi_4
euforbia	De Paralisi_4
pulpe coloquintide	De Paralisi_4
elacterides	De Paralisi_4
mastic	De Paralisi_4
zinziberis	De Paralisi_4
bdellium	De Paralisi_4
sal gemme	De Paralisi_4
serapinum	De Paralisi_4
castor	De Paralisi_4

carvi agreste	De Paralisi_4
sedes of ruta	De Paralisi_4
yerarufini	De Paralisi_4
yeralogodion	De Paralisi_4
yerapigra	De Paralisi_4
oximel squillitik	De Paralisi_4
gummi ruta agreste	De Paralisi_5
euforbia	De Paralisi_5
opoponac	De Paralisi_5
serapinum	De Paralisi_5
galbanum	De Paralisi_5
castor	De Paralisi_5
mirre	De Paralisi_5
asa fetida	De Paralisi_5
bdellium	De Paralisi_5
piretre	De Paralisi_5
nux muscata	De Paralisi_5
nux romane	De Paralisi_5
diatrion pipereon	De Paralisi_5
anacardus	De Paralisi_5
tapsia	De Paralisi_5
nigella	De Paralisi_5
sinapis	De Paralisi_5
carvi agreste	De Paralisi_5
sedes of ruta	De Paralisi_5
oximel squillitik	De Paralisi_5
asa fetida	De Spasmo_1_a
opoponac	De Spasmo_1_a
castor	De Spasmo_1_a
oximel squillitik	De Spasmo_1_b
yerarufini	De Spasmo_1_b
yeralogodion	De Spasmo_1_b
euforbia	De Spasmo_1_b

pulpe coloquintide	De Spasmo_1_b
sticados	De Tremore Membrorum_1
caprifolium	De Tremore Membrorum_1
eupatorie	De Tremore Membrorum_1
pilocelle	De Tortura_1
ciclamin	De Tortura_1
nux indica	De Tortura_1
rote of cucumeris asinini	De Tortura_1
nux muscata	De Tortura_1
acorus	De Tortura_1
piretre	De Tortura_1
wyn	De Tortura_1
vitriol	De Ungue in Conjunctiva_1
viridis eris ust	De Ungue in Conjunctiva_1
sal armoniac	De Ungue in Conjunctiva_1
bole galle	De Ungue in Conjunctiva_1
aloen	De Ungue in Conjunctiva_1
cinamome	De Ungue in Conjunctiva_1
zinziberis	De Ungue in Conjunctiva_1
diatrion pipereon	De Ungue in Conjunctiva_1
fragmentorum scutelle terre viridis	De Ungue in Conjunctiva_1
juse of fenel	De Ungue in Conjunctiva_1
juse of majorane	De Ungue in Conjunctiva_1
hony	De Ungue in Conjunctiva_1
sal armoniac	De Macula Rubea Super Conjunctivam_1
vitriol	De Macula Rubea Super Conjunctivam_1
sarcocolla	De Macula Rubea Super Conjunctivam_1
aloen	De Macula Rubea Super Conjunctivam_1
piperis	De Macula Rubea Super Conjunctivam_1
olibanum	De Macula Rubea Super Conjunctivam_1
eris ust	De Macula Rubea Super Conjunctivam_1
coral	De Macula Rubea Super Conjunctivam_1
margarite	De Macula Rubea Super Conjunctivam_1

dragaganti	De Macula Rubea Super Conjunctivam_1
gummi arabic	De Macula Rubea Super Conjunctivam_1
ceruse	De Macula Rubea Super Conjunctivam_1
amidum	De Macula Rubea Super Conjunctivam_1
sanguis draconis	De Macula Rubea Super Conjunctivam_1
culver blode	De Macula Rubea Super Conjunctivam_1
womman mylke	De Macula Rubea Super Conjunctivam_1
acacia	De Panniculo in Conjunctiva_1
gummi arabic	De Panniculo in Conjunctiva_1
eris ust	De Panniculo in Conjunctiva_1
water	De Panniculo in Conjunctiva_1
eris ust	De Tumore Conjunctiva_1
margarite	De Tumore Conjunctiva_1
coral	De Tumore Conjunctiva_1
spicenardi	De Tumore Conjunctiva_1
musco	De Tumore Conjunctiva_1
sanguis draconis	De Tumore Conjunctiva_1
water of rosis	De Tumore Conjunctiva_1
cathimia aure	De Ulcere et Sanie in Conjunctiva_1
eris ust	De Ulcere et Sanie in Conjunctiva_1
ceruse ablute	De Ulcere et Sanie in Conjunctiva_1
plumbum ust	De Ulcere et Sanie in Conjunctiva_1
dragaganti	De Ulcere et Sanie in Conjunctiva_1
gummi arabic	De Ulcere et Sanie in Conjunctiva_1
aloen	De Ulcere et Sanie in Conjunctiva_1
mirre	De Ulcere et Sanie in Conjunctiva_1
musco	De Ulcere et Sanie in Conjunctiva_1
olibanum	De Ulcere et Sanie in Conjunctiva_1
sarcocolla nutrite	De Ulcere et Sanie in Conjunctiva_1
opium	De Ulcere et Sanie in Conjunctiva_1
water of rosis	De Ulcere et Sanie in Conjunctiva_1
aloen	De Excrescencia et Diminucione Carnis in Conjunctiva_1
olibanum	De Excrescencia et Diminucione Carnis in Conjunctiva_1

sanguis draconis	De Excrescencia et Diminucione Carnis in Conjunctiva_1
sumac	De Excrescencia et Diminucione Carnis in Conjunctiva_1
water of rosis	De Excrescencia et Diminucione Carnis in Conjunctiva_1
gummi arabic	De Excrescencia et Diminucione Carnis in Conjunctiva_1
viridis eris	De Fistula in Lacrimali_1
atrament	De Fistula in Lacrimali_1
sal armoniac	De Fistula in Lacrimali_1
vitriol	De Fistula in Lacrimali_1
calx vive	De Fistula in Lacrimali_1
auripigment	De Fistula in Lacrimali_1
cantaride	De Fistula in Lacrimali_1
alum	De Fistula in Lacrimali_1
urine of a child	De Fistula in Lacrimali_1
oil	De Fistula in Lacrimali_1
levys of agrimonie	De Fistula in Lacrimali_2
levys of olive	De Fistula in Lacrimali_2
whit wyn	De Fistula in Lacrimali_2
aloen	De Lacrimis_1
sarcocolla nutrite	De Lacrimis_1
sumac	De Lacrimis_1
olibanum	De Lacrimis_1
rosis	De Lacrimis_2
margarite	De Lacrimis_2
pulpe mirabolan	De Lacrimis_2
coral red, whit	De Lacrimis_2
camphor	De Lacrimis_2
acacia	De Lacrimis_2
sanguis draconis	De Lacrimis_2
sumac	De Lacrimis_2
rind of pomegarnettes	De Lacrimis_2
opium	De Lacrimis_2
olibanum	De Lacrimis_2
water of dragaganti	De Lacrimis_2

gummi arabic	De Lacrimis_2
tutie preparate	De Lacrimis_3
sarcocolla nutrite	De Lacrimis_3
mirre	De Lacrimis_3
olibanum	De Lacrimis_3
aloen	De Lacrimis_3
mastic	De Lacrimis_3
storace calamite	De Lacrimis_3
ambra grisie	De Lacrimis_3
rosis	De Lacrimis_3
sumac	De Lacrimis_3
sanguis draconis	De Lacrimis_3
muscilage of dragaganti	De Lacrimis_3
gummi arabic	De Lacrimis_3
ceruse ablute	De Ulceribus et Plagis Cornee_1
dragaganti	De Ulceribus et Plagis Cornee_1
gummi arabic	De Ulceribus et Plagis Cornee_1
opium	De Ulceribus et Plagis Cornee_1
ei whit	De Ulceribus et Plagis Cornee_1
womman mylke	De Ulceribus et Plagis Cornee_1
tutie preparate	De Vesica Cornee_1
cathimia lote	De Vesica Cornee_1
antimonie ablute	De Vesica Cornee_1
cicoree	De Vesica Cornee_1
eris ablute	De Vesica Cornee_1
opium	De Vesica Cornee_1
muscilage of fenigrec	De Vesica Cornee_1
antimonie ust, ablute	De Ruptura Cornee_1
ceruse ust, ablute	De Ruptura Cornee_1
climie ust, ablute	De Ruptura Cornee_1
acacia	De Ruptura Cornee_1
spicenardi	De Ruptura Cornee_1
aloen	De Ruptura Cornee_1

tutie nutrite	De Ruptura Cornee_1
sarcocolla nutrite	De Ruptura Cornee_1
muscilage of dragaganti	De Ruptura Cornee_1
ceruse ablute	De Cancro in Cornea_1
amidum	De Cancro in Cornea_1
opium	De Cancro in Cornea_1
ei whit	De Cancro in Cornea_1
muscilage of dragaganti	De Cancro in Cornea_1
water of rosis	De Cancro in Cornea_1
tutie preparate	De Cancro in Cornea_2
amidum	De Cancro in Cornea_2
memithe	De Cancro in Cornea_2
coral red, whit	De Cancro in Cornea_2
terra sigillata	De Cancro in Cornea_2
margarite	De Cancro in Cornea_2
muscilage of dragaganti	De Cancro in Cornea_2
gummi arabic	De Cancro in Cornea_2
galle	De Mutacione Coloris Cornee_1
mirabolan	De Mutacione Coloris Cornee_1
oil of sisamie	De Mutacione Coloris Cornee_1
oil of notys	De Mutacione Coloris Cornee_1
juse of red papaver	De Albugine et Macula et Cicatrice in Oculis_1
juse of centorie minor	De Albugine et Macula et Cicatrice in Oculis_1
hony	De Albugine et Macula et Cicatrice in Oculis_1
juse of lingua passerys	De Albugine et Macula et Cicatrice in Oculis_1_a
ossis cepie	De Albugine et Macula et Cicatrice in Oculis_2
zinziberis	De Albugine et Macula et Cicatrice in Oculis_2
piperis	De Albugine et Macula et Cicatrice in Oculis_2
tutie	De Albugine et Macula et Cicatrice in Oculis_3_a
calamine	De Albugine et Macula et Cicatrice in Oculis_3_a
lignum aloes	De Albugine et Macula et Cicatrice in Oculis_3_a
cuperose	De Albugine et Macula et Cicatrice in Oculis_3_a
caprifolium	De Albugine et Macula et Cicatrice in Oculis_3_a

urine of a mayde child	De Albugine et Macula et Cicatrice in Oculis_3_a
water of rosis	De Albugine et Macula et Cicatrice in Oculis_3_a
juse of majorane	De Albugine et Macula et Cicatrice in Oculis_3_a
water of fenel	De Albugine et Macula et Cicatrice in Oculis_3_b
celidonie	De Albugine et Macula et Cicatrice in Oculis_3_b
swynes grece recent	De Albugine et Macula et Cicatrice in Oculis_3_c
sarcocolla nutrite	De Albugine et Macula et Cicatrice in Oculis_4_a
tutie preparate	De Albugine et Macula et Cicatrice in Oculis_4_a
aloen	De Albugine et Macula et Cicatrice in Oculis_4_a
piperis long	De Albugine et Macula et Cicatrice in Oculis_4_a
corticis testarum ovorum calcinatarum	De Albugine et Macula et Cicatrice in Oculis_4_a
fragmentorum scutelle terre viridis	De Albugine et Macula et Cicatrice in Oculis_4_a
vitriol	De Albugine et Macula et Cicatrice in Oculis_4_a
eris ust	De Albugine et Macula et Cicatrice in Oculis_4_a
ceruse lote	De Albugine et Macula et Cicatrice in Oculis_4_a
stercoris lacerte viridis	De Albugine et Macula et Cicatrice in Oculis_4_a
cathimia aure	De Albugine et Macula et Cicatrice in Oculis_4_a
margarite	De Albugine et Macula et Cicatrice in Oculis_4_a
coral	De Albugine et Macula et Cicatrice in Oculis_4_a
viridis eris	De Albugine et Macula et Cicatrice in Oculis_4_a
castor	De Albugine et Macula et Cicatrice in Oculis_4_a
mirre	De Albugine et Macula et Cicatrice in Oculis_4_a
caprifolium	De Albugine et Macula et Cicatrice in Oculis_4_a
musco	De Albugine et Macula et Cicatrice in Oculis_4_a
dragaganti	De Albugine et Macula et Cicatrice in Oculis_4_a
serapinum	De Albugine et Macula et Cicatrice in Oculis_4_a
galbanum	De Albugine et Macula et Cicatrice in Oculis_4_a
spume maris	De Albugine et Macula et Cicatrice in Oculis_4_a
opium	De Albugine et Macula et Cicatrice in Oculis_4_a
wulturis galle	De Albugine et Macula et Cicatrice in Oculis_4_a
balsamum	De Albugine et Macula et Cicatrice in Oculis_4_a
ambra grisie	De Albugine et Macula et Cicatrice in Oculis_4_a
limature auri purissimi	De Albugine et Macula et Cicatrice in Oculis_4_a

juse of eufrasie	De Albugine et Macula et Cicatrice in Oculis_4_a
womman mylke	De Albugine et Macula et Cicatrice in Oculis_4_a
juse of fenel	De Albugine et Macula et Cicatrice in Oculis_4_b
juse of celidonie	De Albugine et Macula et Cicatrice in Oculis_4_b
swynes grece	De Albugine et Macula et Cicatrice in Oculis_4_c
farina of barlich	De Pascionibus Uvee Sicut Est Delatacione Pupille_1
faba	De Pascionibus Uvee Sicut Est Delatacione Pupille_1
ei yolk	De Pascionibus Uvee Sicut Est Delatacione Pupille_1
oil of rosis	De Pascionibus Uvee Sicut Est Delatacione Pupille_1
juse of endive	De Pascionibus Uvee Sicut Est Delatacione Pupille_1
womman mylke	De Pascionibus Uvee Sicut Est Delatacione Pupille_1
culver blode	De Pascionibus Uvee Sicut Est Delatacione Pupille_1
crocus	De Constriccione Pupille_1
musco	De Constriccione Pupille_1
aloen	De Constriccione Pupille_1
stercoris lacerte viridis	De Constriccione Pupille_1
water of fenel	De Constriccione Pupille_1
ancipiter galle	De Aquis et Cataractis_1_a
balsamum	De Aquis et Cataractis_1_a
ellebore whit	De Aquis et Cataractis_1_a
piperis	De Aquis et Cataractis_1_a
water of raphane	De Aquis et Cataractis_1_a
majorane	De Aquis et Cataractis_1_b
majorane	Curacio Particularis Debilitatis Visus et Potissime cum Oculi Videntur Sani et Visus Debilitatur_1
salvie	Curacio Particularis Debilitatis Visus et Potissime cum Oculi Videntur Sani et Visus Debilitatur_1
sileris montani	Curacio Particularis Debilitatis Visus et Potissime cum Oculi Videntur Sani et Visus Debilitatur_1
ruta	Curacio Particularis Debilitatis Visus et Potissime cum Oculi Videntur Sani et Visus Debilitatur_1
eufrasie vere montane	Curacio Particularis Debilitatis Visus et Potissime cum Oculi Videntur Sani et Visus Debilitatur_1

saxifragie	Curacio Particularis Debilitatis Visus et Potissime cum Oculi Videntur Sani et Visus Debilitatur_1
fragarie	Curacio Particularis Debilitatis Visus et Potissime cum Oculi Videntur Sani et Visus Debilitatur_1
anis	Curacio Particularis Debilitatis Visus et Potissime cum Oculi Videntur Sani et Visus Debilitatur_1
fenel	Curacio Particularis Debilitatis Visus et Potissime cum Oculi Videntur Sani et Visus Debilitatur_1
dauci	Curacio Particularis Debilitatis Visus et Potissime cum Oculi Videntur Sani et Visus Debilitatur_1
carvi	Curacio Particularis Debilitatis Visus et Potissime cum Oculi Videntur Sani et Visus Debilitatur_1
hony	Curacio Particularis Debilitatis Visus et Potissime cum Oculi Videntur Sani et Visus Debilitatur_1
yerapigra	Curacio Particularis Debilitatis Visus et Potissime cum Oculi Videntur Sani et Visus Debilitatur_2
yeralogodion	Curacio Particularis Debilitatis Visus et Potissime cum Oculi Videntur Sani et Visus Debilitatur_2
euforbia	Curacio Particularis Debilitatis Visus et Potissime cum Oculi Videntur Sani et Visus Debilitatur_2
pulpe coloquintide	Curacio Particularis Debilitatis Visus et Potissime cum Oculi Videntur Sani et Visus Debilitatur_2
mastic	Curacio Particularis Debilitatis Visus et Potissime cum Oculi Videntur Sani et Visus Debilitatur_2
anis	Curacio Particularis Debilitatis Visus et Potissime cum Oculi Videntur Sani et Visus Debilitatur_2
bdellium	Curacio Particularis Debilitatis Visus et Potissime cum Oculi Videntur Sani et Visus Debilitatur_2
gariofilum	Curacio Particularis Debilitatis Visus et Potissime cum Oculi Videntur Sani et Visus Debilitatur_2
juse of absinthium	Curacio Particularis Debilitatis Visus et Potissime cum Oculi Videntur Sani et Visus Debilitatur_2

diasene	Curacio Particularis Debilitatis Visus et Potissime cum Oculi Videntur Sani et Visus Debilitatur_3
diaborage	Curacio Particularis Debilitatis Visus et Potissime cum Oculi Videntur Sani et Visus Debilitatur_3
yerarufini	Curacio Particularis Debilitatis Visus et Potissime cum Oculi Videntur Sani et Visus Debilitatur_3
lapidis armenici nouies abluti	Curacio Particularis Debilitatis Visus et Potissime cum Oculi Videntur Sani et Visus Debilitatur_3
celidonie	Curacio Particularis Debilitatis Visus et Potissime cum Oculi Videntur Sani et Visus Debilitatur_4
fenel	Curacio Particularis Debilitatis Visus et Potissime cum Oculi Videntur Sani et Visus Debilitatur_4
ruta	Curacio Particularis Debilitatis Visus et Potissime cum Oculi Videntur Sani et Visus Debilitatur_4
sileris montani	Curacio Particularis Debilitatis Visus et Potissime cum Oculi Videntur Sani et Visus Debilitatur_4
eufrasie	Curacio Particularis Debilitatis Visus et Potissime cum Oculi Videntur Sani et Visus Debilitatur_4
verbene	Curacio Particularis Debilitatis Visus et Potissime cum Oculi Videntur Sani et Visus Debilitatur_4
red rosis	Curacio Particularis Debilitatis Visus et Potissime cum Oculi Videntur Sani et Visus Debilitatur_4
gariofilum	Curacio Particularis Debilitatis Visus et Potissime cum Oculi Videntur Sani et Visus Debilitatur_4
piperis long	Curacio Particularis Debilitatis Visus et Potissime cum Oculi Videntur Sani et Visus Debilitatur_4
eufrasie	Curacio Particularis Debilitatis Visus et Potissime cum Oculi Videntur Sani et Visus Debilitatur_5
salvie	Curacio Particularis Debilitatis Visus et Potissime cum Oculi Videntur Sani et Visus Debilitatur_5
mente	Curacio Particularis Debilitatis Visus et Potissime cum Oculi Videntur Sani et Visus Debilitatur_5

sileris montani	Curacio Particularis Debilitatis Visus et Potissime cum Oculi Videntur Sani et Visus Debilitatur_5
anis	Curacio Particularis Debilitatis Visus et Potissime cum Oculi Videntur Sani et Visus Debilitatur_5
fenel	Curacio Particularis Debilitatis Visus et Potissime cum Oculi Videntur Sani et Visus Debilitatur_5
zinziberis	Curacio Particularis Debilitatis Visus et Potissime cum Oculi Videntur Sani et Visus Debilitatur_5
gariofilum	Curacio Particularis Debilitatis Visus et Potissime cum Oculi Videntur Sani et Visus Debilitatur_5
piperis long	Curacio Particularis Debilitatis Visus et Potissime cum Oculi Videntur Sani et Visus Debilitatur_5
floures of borage	Curacio Particularis Debilitatis Visus et Potissime cum Oculi Videntur Sani et Visus Debilitatur_5
floures of sene	Curacio Particularis Debilitatis Visus et Potissime cum Oculi Videntur Sani et Visus Debilitatur_5
epithimi	Curacio Particularis Debilitatis Visus et Potissime cum Oculi Videntur Sani et Visus Debilitatur_5
liquirice	Curacio Particularis Debilitatis Visus et Potissime cum Oculi Videntur Sani et Visus Debilitatur_5
uva passe	Curacio Particularis Debilitatis Visus et Potissime cum Oculi Videntur Sani et Visus Debilitatur_5
rind of mirabolan indorum	Curacio Particularis Debilitatis Visus et Potissime cum Oculi Videntur Sani et Visus Debilitatur_5
sugre	Curacio Particularis Debilitatis Visus et Potissime cum Oculi Videntur Sani et Visus Debilitatur_5
sileris montani	Curacio Particularis Debilitatis Visus et Potissime cum Oculi Videntur Sani et Visus Debilitatur_6
majorane	Curacio Particularis Debilitatis Visus et Potissime cum Oculi Videntur Sani et Visus Debilitatur_6
eufrasie	Curacio Particularis Debilitatis Visus et Potissime cum Oculi Videntur Sani et Visus Debilitatur_6

ruta	Curacio Particularis Debilitatis Visus et Potissime cum Oculi Videntur Sani et Visus Debilitatur_6
celidonie	Curacio Particularis Debilitatis Visus et Potissime cum Oculi Videntur Sani et Visus Debilitatur_6
fenel	Curacio Particularis Debilitatis Visus et Potissime cum Oculi Videntur Sani et Visus Debilitatur_6
zinziberis	Curacio Particularis Debilitatis Visus et Potissime cum Oculi Videntur Sani et Visus Debilitatur_6
spicenardi	Curacio Particularis Debilitatis Visus et Potissime cum Oculi Videntur Sani et Visus Debilitatur_6
piperis long	Curacio Particularis Debilitatis Visus et Potissime cum Oculi Videntur Sani et Visus Debilitatur_6
gariofilum	Curacio Particularis Debilitatis Visus et Potissime cum Oculi Videntur Sani et Visus Debilitatur_6
tutie extinct	Curacio Particularis Debilitatis Visus et Potissime cum Oculi Videntur Sani et Visus Debilitatur_6
sarcocolla nutrite	Curacio Particularis Debilitatis Visus et Potissime cum Oculi Videntur Sani et Visus Debilitatur_6
lignum aloes	Curacio Particularis Debilitatis Visus et Potissime cum Oculi Videntur Sani et Visus Debilitatur_6
ancipiter galle	Curacio Particularis Debilitatis Visus et Potissime cum Oculi Videntur Sani et Visus Debilitatur_6
aquile galle	Curacio Particularis Debilitatis Visus et Potissime cum Oculi Videntur Sani et Visus Debilitatur_6
hirci galle	Curacio Particularis Debilitatis Visus et Potissime cum Oculi Videntur Sani et Visus Debilitatur_6
balsamum	Curacio Particularis Debilitatis Visus et Potissime cum Oculi Videntur Sani et Visus Debilitatur_6
mel roset	Curacio Particularis Debilitatis Visus et Potissime cum Oculi Videntur Sani et Visus Debilitatur_6
eris ust	De Scabie Pruritu, Asperitate et Rubore_1
cathimia lote	De Scabie Pruritu, Asperitate et Rubore_1
ceruse lote	De Scabie Pruritu, Asperitate et Rubore_1

aloen	De Scabie Pruritu, Asperitate et Rubore_1
mirre	De Scabie Pruritu, Asperitate et Rubore_1
sal armoniac	De Scabie Pruritu, Asperitate et Rubore_1
floris eris	De Scabie Pruritu, Asperitate et Rubore_1
hony	De Scabie Pruritu, Asperitate et Rubore_1
armoniac	De Nodis seu Lippa et Petrificacione in Palpebrarum_1
opoponac	De Nodis seu Lippa et Petrificacione in Palpebrarum_1
galbanum	De Nodis seu Lippa et Petrificacione in Palpebrarum_1
stronge vinegre	De Nodis seu Lippa et Petrificacione in Palpebrarum_1
ferment	De Ordeolo_1
musco	De Ordeolo_1
lapdanum	De Ordeolo_1
mirre	De Ordeolo_1
olibanum	De Ordeolo_1
nitrum	De Ordeolo_1
oil of lilie	De Ordeolo_1
aloen	De Carnositate in Palpebra Exteriori_1_a
memithe	De Carnositate in Palpebra Exteriori_1_a
sarcocolla nutrite	De Carnositate in Palpebra Exteriori_1_a
litarge	De Carnositate in Palpebra Exteriori_1_a
juse of celidonie	De Carnositate in Palpebra Exteriori_1_a
sal	De Carnositate in Palpebra Exteriori_1_b
comin	De Carnositate in Palpebra Exteriori_1_b
ei whit	De Carnositate in Palpebra Exteriori_1_b
sumac	De Pruritu Palpebrarum_1_a
sedes of portulaca	De Pruritu Palpebrarum_1_a
lentiginis	De Pruritu Palpebrarum_1_a
oil of rosis	De Pruritu Palpebrarum_1_a
ei whit	De Pruritu Palpebrarum_1_a
sarcocolla nutrite	De Pruritu Palpebrarum_1_b
juse of celidonie	De Pruritu Palpebrarum_1_b
spicenardi	De Casu Pilorum Palpebris_1
mouse tordys	De Casu Pilorum Palpebris_1

lapdanum	De Casu Pilorum Palpebris_1
bonis of datis ybrend	De Casu Pilorum Palpebris_1
swalewys tordys	De Casu Pilorum Palpebris_1
hony	De Casu Pilorum Palpebris_1
sal armoniac	De Pediculis Palpebrarum_1
aloen cicotrini	De Pediculis Palpebrarum_1
alum	De Pediculis Palpebrarum_1
stafisagre	De Pediculis Palpebrarum_1
vinegre squillitik	De Pediculis Palpebrarum_1
farina of volatilis molendini	De Dolore Auris et De Apostemate Auris_1
granorum pini	De Dolore Auris et De Apostemate Auris_1
muscilage of fenigrec	De Dolore Auris et De Apostemate Auris_1
sedes of linsed	De Dolore Auris et De Apostemate Auris_1
resin of olibanum	De Dolore Auris et De Apostemate Auris_1
litarge	De Dolore Auris et De Apostemate Auris_1
ceruse	De Dolore Auris et De Apostemate Auris_1
whit vinegre	De Dolore Auris et De Apostemate Auris_1
oil of sisamie	De Dolore Auris et De Apostemate Auris_1
swynes grece recent	De Dolore Auris et De Apostemate Auris_1
cantaride	De Dolore Auris et De Apostemate Auris_2
oil of scorpiouns	De Dolore Auris et De Apostemate Auris_2
bole galle	De Dolore Auris et De Apostemate Auris_2
majorane	De Dolore Auris et De Apostemate Auris_3
spicenardi	De Dolore Auris et De Apostemate Auris_3
oil	De Dolore Auris et De Apostemate Auris_3
mirre	De Dolore Auris et De Apostemate Auris_4
olibanum	De Dolore Auris et De Apostemate Auris_4
caprifolium	De Dolore Auris et De Apostemate Auris_4
galbanum	De Dolore Auris et De Apostemate Auris_4
nitrum	De Dolore Auris et De Apostemate Auris_4
crocus	De Dolore Auris et De Apostemate Auris_4
almaundes	De Dolore Auris et De Apostemate Auris_4
vinegre	De Dolore Auris et De Apostemate Auris_4

oil of rosis	De Dolore Auris et De Apostemate Auris_4
conchilia marina alba	De Dolore Auris et De Apostemate Auris_5
oximel	De Dolore Auris et De Apostemate Auris_5
carica	De Dolore Auris et De Apostemate Auris_6
ley	De Dolore Auris et De Apostemate Auris_6
juse of leke	De Sanguine Fluente ab Auribus_1_a
whit wyn	De Sanguine Fluente ab Auribus_1_a
juse of plantago	De Sanguine Fluente ab Auribus_1_a
juse of bursa pastoris	De Sanguine Fluente ab Auribus_1_a
acacia	De Sanguine Fluente ab Auribus_1_b
psidia	De Sanguine Fluente ab Auribus_1_b
balaustia	De Sanguine Fluente ab Auribus_1_b
galle	De Sanguine Fluente ab Auribus_1_b
sumac	De Sanguine Fluente ab Auribus_1_b
mastic	De Sanguine Fluente ab Auribus_1_b
olibanum	De Sanguine Fluente ab Auribus_1_b
vinegre	De Sanguine Fluente ab Auribus_1_b
litarge	De Pascionibus Narium_1
ceruse	De Pascionibus Narium_1
cathimia	De Pascionibus Narium_1
galle	De Pascionibus Narium_1
nitrum	De Pascionibus Narium_1
alum	De Pascionibus Narium_1
oil of camomille	De Pascionibus Narium_1
wex	De Pascionibus Narium_1
gummi arabic	De Pascionibus Narium_2
medulla vitulli	De Pascionibus Narium_2
dragaganti	De Pascionibus Narium_2
oil of sisamie	De Pascionibus Narium_2
whit wex	De Pascionibus Narium_2
sedes of majorane	De Pascionibus Narium_3
basilicon	De Pascionibus Narium_3
gariofilum	De Pascionibus Narium_3

nux muscata	De Pascionibus Narium_3
cinamome	De Pascionibus Narium_3
lignum aloes	De Pascionibus Narium_3
storace calamite	De Pascionibus Narium_3
ambra grisie	De Pascionibus Narium_3
musco	De Pascionibus Narium_3
water of rosis	De Pascionibus Narium_3
papaver	De Reumate et Coriza_1
rosis	De Reumate et Coriza_1
lactuca	De Reumate et Coriza_1
candi penidiarum	De Reumate et Coriza_1
sandali	De Reumate et Coriza_1
barlich	De Reumate et Coriza_1
hony	De Reumate et Coriza_1
nenifar	De Reumate et Coriza_1
sugre	De Reumate et Coriza_1
water of barlich	De Reumate et Coriza_1
diapapaver	De Reumate et Coriza_2
diadragagantum	De Reumate et Coriza_2
sugre of rosis	De Reumate et Coriza_2
oil of rosis	De Reumate et Coriza_2
oil of violet	De Reumate et Coriza_2
rote of yreos	De Reumate et Coriza_3
ysope	De Reumate et Coriza_3
liquirice	De Reumate et Coriza_3
nucis of cipresse	De Reumate et Coriza_3
levys of cipresse	De Reumate et Coriza_3
mastic	De Reumate et Coriza_3
olibanum	De Reumate et Coriza_3
mel roset	De Reumate et Coriza_3
stercus columbinum	De Reumate et Coriza_4
gummi ruta agreste	De Reumate et Coriza_4
picis navalis	De Reumate et Coriza_4

sedes of ruta	De Reumate et Coriza_4
sinapis	De Reumate et Coriza_4
oil of laurel	De Reumate et Coriza_4
vinegre squillitik	De Reumate et Coriza_4
sedes of whit papaver	De Reumate et Coriza_5
sedes of portulaca	De Reumate et Coriza_5
sedes of lactuca	De Reumate et Coriza_5
candi penidiarum	De Reumate et Coriza_5
dragaganti	De Reumate et Coriza_5
opium	De Reumate et Coriza_5
crocus	De Reumate et Coriza_5
sirup of mirtille	De Reumate et Coriza_5
sirup of papaver	De Reumate et Coriza_5
storace calamite	De Reumate et Coriza_6
nucis of cipresse	De Reumate et Coriza_6
mastic	De Reumate et Coriza_6
olibanum	De Reumate et Coriza_6
lapdanum	De Reumate et Coriza_6
ambra grisie	De Reumate et Coriza_6
liquirice	De Reumate et Coriza_6
uva passe	De Reumate et Coriza_6
ydromel	De Reumate et Coriza_6
ceruse lote	De Fissura Labioram_1
amidum	De Fissura Labioram_1
cathimia lote	De Fissura Labioram_1
litarge	De Fissura Labioram_1
muscilage of dragaganti	De Fissura Labioram_1
uva passe	De Pascionibus Lingue_1
jujube	De Pascionibus Lingue_1
sebesten	De Pascionibus Lingue_1
fenigrec	De Pascionibus Lingue_1
carica	De Pascionibus Lingue_1
water	De Pascionibus Lingue_1

cassiafistula	De Pascionibus Lingue_1
wex	De Pascionibus Lingue_1
sirup of nenifar	De Pascionibus Lingue_1
pomes acetosite	De Pascionibus Lingue_2
juse of acetose	De Pascionibus Lingue_2
vinegre	De Pascionibus Lingue_2
water of rosis	De Pascionibus Lingue_2
wyn of pomegarnettes	De Pascionibus Lingue_2
sedes of citonie	De Pascionibus Lingue_2
psillie	De Pascionibus Lingue_2
dragaganti	De Pascionibus Lingue_2
sugre	De Pascionibus Lingue_2
rote of cucumeris asinini	De Perforacione, Putredione et Vermibus Dencium_1
rote of coloquintide	De Perforacione, Putredione et Vermibus Dencium_1
rote of celsi	De Perforacione, Putredione et Vermibus Dencium_1
scharpe vinegre	De Perforacione, Putredione et Vermibus Dencium_1
mylke of titimalle	De Perforacione, Putredione et Vermibus Dencium_1
wex	De Perforacione, Putredione et Vermibus Dencium_1
auripigment	De Perforacione, Putredione et Vermibus Dencium_2
rote of capparis	De Perforacione, Putredione et Vermibus Dencium_2
rote of coloquintide	De Perforacione, Putredione et Vermibus Dencium_2
wyn	De Perforacione, Putredione et Vermibus Dencium_2
sal assi	De Perforacione, Putredione et Vermibus Dencium_3
comin assi	De Perforacione, Putredione et Vermibus Dencium_3
laurel	De Perforacione, Putredione et Vermibus Dencium_3
aristologie long	De Perforacione, Putredione et Vermibus Dencium_3
cinamome	De Perforacione, Putredione et Vermibus Dencium_3
piperis	De Perforacione, Putredione et Vermibus Dencium_3
nucis of cipresse	De Perforacione, Putredione et Vermibus Dencium_3
alum assi	De Perforacione, Putredione et Vermibus Dencium_3
ossis cepie	De Perforacione, Putredione et Vermibus Dencium_4
conchilia marina alba	De Perforacione, Putredione et Vermibus Dencium_4
porcellane	De Perforacione, Putredione et Vermibus Dencium_4

spume maris	De Perforacione, Putredione et Vermibus Dencium_4
nitrum	De Perforacione, Putredione et Vermibus Dencium_4
alum	De Perforacione, Putredione et Vermibus Dencium_4
aristologie	De Perforacione, Putredione et Vermibus Dencium_4
rosis	De Perforacione, Putredione et Vermibus Dencium_4
sal gemme	De Perforacione, Putredione et Vermibus Dencium_4
rote of canna assi	De Perforacione, Putredione et Vermibus Dencium_4
barlich ust	De Perforacione, Putredione et Vermibus Dencium_4
sulfur combust	De Perforacione, Putredione et Vermibus Dencium_4
cornu cervi combust	De Perforacione, Putredione et Vermibus Dencium_4
cinamome	De Perforacione, Putredione et Vermibus Dencium_4
piperis long	De Perforacione, Putredione et Vermibus Dencium_4
alum	De Pascionibus Gingivarum_1_a
sal commune assi	De Pascionibus Gingivarum_1_a
psidia	De Pascionibus Gingivarum_1_a
balaustia	De Pascionibus Gingivarum_1_a
galle	De Pascionibus Gingivarum_1_a
cupula glandium	De Pascionibus Gingivarum_1_a
rosis	De Pascionibus Gingivarum_1_a
rote of aristologie	De Pascionibus Gingivarum_1_a
piperis long	De Pascionibus Gingivarum_1_a
cinamome	De Pascionibus Gingivarum_1_a
spicenardi	De Pascionibus Gingivarum_1_a
liquirice	De Pascionibus Gingivarum_1_a
gummi arabic	De Pascionibus Gingivarum_1_a
dragaganti	De Pascionibus Gingivarum_1_a
vinegre	De Pascionibus Gingivarum_1_a
hony	De Pascionibus Gingivarum_1_a
levys of olive	De Pascionibus Gingivarum_1_b
wyn	De Pascionibus Gingivarum_1_b
oximel squillitik	De Pascionibus Gingivarum_1_b
ellebore	De Pascionibus Gingivarum_2
aristologie	De Pascionibus Gingivarum_2

tartar	De Pascionibus Gingivarum_2
filtri combusti	De Pascionibus Gingivarum_2
alum assi	De Pascionibus Gingivarum_2
stercus columbinum	De Pascionibus Gingivarum_2
fecis ferri combusti	De Pascionibus Gingivarum_2
galle	De Pascionibus Gingivarum_2
balaustia	De Pascionibus Gingivarum_2
juse of olive levys	De Pascionibus Gingivarum_2
hony	De Pascionibus Gingivarum_2
calx vive	De Pascionibus Gingivarum_3
arsenic	De Pascionibus Gingivarum_3
vitriol	De Pascionibus Gingivarum_3
viridis eris	De Pascionibus Gingivarum_3
galle	De Pascionibus Gingivarum_3
psidia	De Pascionibus Gingivarum_3
balaustia	De Pascionibus Gingivarum_3
oximel squillitik	De Pascionibus Gingivarum_3
swete wyn	De Pascionibus Gingivarum_3
sedes of rosis	De Squinancia_1_a
sumac	De Squinancia_1_a
dragaganti frigidi	De Squinancia_1_a
sirup of nenifar	De Squinancia_1_a
oximel	De Squinancia_1_b
water	De Squinancia_1_b
vinegre	De Squinancia_1_b
mastic	De Squinancia_2
olibanum	De Squinancia_2
uva passe	De Squinancia_2
storace calamite	De Squinancia_2
ydromel	De Squinancia_2
sinapis	De Squinancia_3
nitrum	De Squinancia_3
sulfur	De Squinancia_3

oil	De Squinancia_3
castor	De Squinancia_3
oil of violet	De Squinancia_4
butere	De Squinancia_4
carica	De Squinancia_4
ferment	De Squinancia_4
dialtee	De Squinancia_4
psillie	De Pascionibus Uvule_1
dragaganti	De Pascionibus Uvule_1
gummi arabic	De Pascionibus Uvule_1
fenigrec	De Pascionibus Uvule_1
sedes of linsed	De Pascionibus Uvule_1
sedes of malve	De Pascionibus Uvule_1
barlich	De Pascionibus Uvule_1
hoot water	De Pascionibus Uvule_1
asa fetida	De Pascionibus Uvule_2
arsenic	De Pascionibus Uvule_2
vitriol	De Pascionibus Uvule_2
sal armoniac	De Pascionibus Uvule_2
atrament	De Pascionibus Uvule_2
alum	De Pascionibus Uvule_2
vinegre squillitik	De Pascionibus Uvule_2
ysope	De Pascionibus Uvule_3
yreos	De Pascionibus Uvule_3
liquirice	De Pascionibus Uvule_3
capilli veneris	De Pascionibus Uvule_3
rosis	De Pascionibus Uvule_3
barlich	De Pascionibus Uvule_3
ydromel	De Pascionibus Uvule_3
oximel diuretik	De Pascionibus Uvule_3
storace calamite	De Rausidine Vocis_1
ambra grisie	De Rausidine Vocis_1
mastic	De Rausidine Vocis_1

olibanum	De Rausidine Vocis_1
liquirice	De Rausidine Vocis_1
uva passe	De Rausidine Vocis_1
carica	De Rausidine Vocis_1
dragaganti	De Rausidine Vocis_1
gummi arabic	De Rausidine Vocis_1
ydromel	De Rausidine Vocis_1
zinziberis	De Rausidine Vocis_2
piperis long	De Rausidine Vocis_2
cubebe	De Rausidine Vocis_2
poudre of calamente	De Rausidine Vocis_2
carica	De Rausidine Vocis_2
uva passe	De Rausidine Vocis_2
pine	De Rausidine Vocis_2
pistace	De Rausidine Vocis_2
hony	De Rausidine Vocis_2
rote of yreos	De Rausidine Vocis_3
lilie	De Rausidine Vocis_3
squille	De Rausidine Vocis_3
ysope	De Rausidine Vocis_3
origane	De Rausidine Vocis_3
calamente	De Rausidine Vocis_3
siseleos	De Rausidine Vocis_3
fenigrec	De Rausidine Vocis_3
carica	De Rausidine Vocis_3
uva passe	De Rausidine Vocis_3
pistace	De Rausidine Vocis_3
pine	De Rausidine Vocis_3
date	De Rausidine Vocis_3
almaundes	De Rausidine Vocis_3
jujube	De Rausidine Vocis_3
sebesten	De Rausidine Vocis_3
hony	De Rausidine Vocis_3

yerapigra	De Rausidine Vocis_4
yeralogodion	De Rausidine Vocis_4
bdellium	De Rausidine Vocis_4
pulpe coloquintide	De Rausidine Vocis_4
diatrion pipereon	De Rausidine Vocis_5
cubebe	De Rausidine Vocis_5
cinamome	De Rausidine Vocis_5
sedes of linsed assi	De Rausidine Vocis_5
fenigrec	De Rausidine Vocis_5
fenel	De Rausidine Vocis_5
pine	De Rausidine Vocis_5
swete almaundes	De Rausidine Vocis_5
liquirice	De Rausidine Vocis_5
carica	De Rausidine Vocis_5
uva passe	De Rausidine Vocis_5
jujube	De Rausidine Vocis_5
sebesten	De Rausidine Vocis_5
hony	De Rausidine Vocis_5
ambra grisie	De Rausidine Vocis_6
storace calamite	De Rausidine Vocis_6
mastic	De Rausidine Vocis_6
olibanum	De Rausidine Vocis_6
dragaganti	De Rausidine Vocis_6
gummi arabic	De Rausidine Vocis_6
ydromel	De Rausidine Vocis_6
mirre	De Rausidine Vocis_6
liquirice	De Rausidine Vocis_6
uva passe	De Rausidine Vocis_6
carica	De Rausidine Vocis_6
mirre	De Rausidine Vocis_7
olibanum	De Rausidine Vocis_7
carica	De Rausidine Vocis_7
juse of an olde henne	De Rausidine Vocis_7

polipodi	De Rausidine Vocis_7
anis	De Rausidine Vocis_7
ysope	De Rausidine Vocis_7
uva passe	De Rausidine Vocis_7
piperis	De Rausidine Vocis_8
cubebe	De Rausidine Vocis_8
mirre	De Rausidine Vocis_8
cost	De Rausidine Vocis_8
aristologie	De Rausidine Vocis_8
cassialignea	De Rausidine Vocis_8
siseleos	De Rausidine Vocis_8
fenel	De Rausidine Vocis_8
arsenic	De Rausidine Vocis_8
sulfur	De Rausidine Vocis_8
cicer	De Rausidine Vocis_8
asa fetida	De Rausidine Vocis_8
gotys talou	De Rausidine Vocis_8
ei yolk	De Rausidine Vocis_8
psillie	De Rausidine Vocis_9
sedes of citonie	De Rausidine Vocis_9
draganti	De Rausidine Vocis_9
water	De Rausidine Vocis_9
4 cold sedes	De Rausidine Vocis_9
sedes of lactuca	De Rausidine Vocis_9
portulaca	De Rausidine Vocis_9
liquirice	De Rausidine Vocis_9
whit papaver	De Rausidine Vocis_9
candi penidiarum	De Rausidine Vocis_9
uva passe	De Rausidine Vocis_9
dragaganti	De Tussi_1
julep	De Tussi_1
cubebe	De Tussi_1
uva passe	De Tussi_1

amidum	De Tussi_1
juse of liquirice	De Tussi_1
sedes of whit papaver	De Tussi_1
sirup of rosis	De Tussi_1
capilli veneris	De Tussi_2
lactuca	De Tussi_2
4 cold sedes	De Tussi_2
sedes of sebesten	De Tussi_2
jujube	De Tussi_2
carica	De Tussi_2
pine	De Tussi_2
liquirice	De Tussi_2
sedes of papaver	De Tussi_2
hony	De Tussi_2
sugre	De Tussi_2
floures of borage	De Tussi_3
floures of violet	De Tussi_3
jujube	De Tussi_3
liquirice	De Tussi_3
prune	De Tussi_3
mastic	De Tussi_3
diagridium	De Tussi_3
cassiafistula	De Tussi_3
tamarinde	De Tussi_3
diapapaver	De Tussi_4
dragaganti frigidi	De Tussi_4
diapenidion sine cinamome	De Tussi_4
sugre	De Tussi_4
hony	De Tussi_4
diayreos	De Tussi_4
4 cold sedes	De Tussi_5
dragaganti	De Tussi_5
gummi arabic	De Tussi_5

candi penidiarum	De Tussi_5
sedes of malve	De Tussi_5
medulla cervi	De Tussi_5
medulla vitulli	De Tussi_5
butere sine sal	De Tussi_5
swynes grece recent	De Tussi_5
oil of violet	De Tussi_5
whit wex	De Tussi_5
mastic	De Tussi_6
olibanum	De Tussi_6
lapdanum	De Tussi_6
camomille	De Tussi_6
mellilote	De Tussi_6
liquirice	De Tussi_6
water	De Tussi_6
rote of yreos	De Tussi_7
ysope	De Tussi_7
sanamunde	De Tussi_7
origane	De Tussi_7
calamente	De Tussi_7
capilli veneris recent	De Tussi_7
serpel	De Tussi_7
carica	De Tussi_7
liquirice	De Tussi_7
jujube	De Tussi_7
pine	De Tussi_7
fenigrec	De Tussi_7
hony	De Tussi_7
sugre	De Tussi_7
yerapigra	De Tussi_8_a
pulpe coloquintide	De Tussi_8_a
mastic	De Tussi_8_a
bdellium	De Tussi_8_a

liquirice	De Tussi_8_a
ydromel	De Tussi_8_a
agarik	De Tussi_8_b
turbith	De Tussi_8_b
diayreos	De Tussi_9
diaysope	De Tussi_9
diacalamente	De Tussi_9
diaprassium	De Tussi_9
red wex	De Tussi_9
oil	De Tussi_9
hony	De Tussi_9
water	De Tussi_9
sulfur vive	De Tussi_10
mirre	De Tussi_10
castor	De Tussi_10
piperis nigrum	De Tussi_10
piperis long	De Tussi_10
storace calamite	De Tussi_10
spicenardi	De Tussi_10
cost	De Tussi_10
galbanum	De Tussi_10
poudre of ysope	De Tussi_10
liquirice	De Tussi_10
opium	De Tussi_10
hony dispumati	De Tussi_10
stercus columbinum	De Tussi_11
mirre	De Tussi_11
storace calamite	De Tussi_11
castor	De Tussi_11
wex	De Tussi_11
carica	De Tussi_11
oil of mastic	De Tussi_11
diatrion pipereon	De Tussi_12

diaprassium	De Tussi_12
rote of yreos	De Ptisi_1
ysope	De Ptisi_1
capilli veneris recent	De Ptisi_1
4 cold sedes	De Ptisi_1
sedes of bombac	De Ptisi_1
sedes of whit papaver	De Ptisi_1
fenigrec	De Ptisi_1
sedes of endive	De Ptisi_1
sedes of scariol	De Ptisi_1
sedes of portulaca	De Ptisi_1
sedes of lactuca	De Ptisi_1
sedes of coriandre	De Ptisi_1
orobus	De Ptisi_1
liquirice	De Ptisi_1
uva passe	De Ptisi_1
carica	De Ptisi_1
jujube	De Ptisi_1
sebesten	De Ptisi_1
pistace	De Ptisi_1
pine	De Ptisi_1
almaundes	De Ptisi_1
barlich	De Ptisi_1
water	De Ptisi_1
mel roset	De Ptisi_1
sugre	De Ptisi_1
croppes of fenel	De Ptisi_2
croppes of anete	De Ptisi_2
croppes of camomille	De Ptisi_2
fenigrec	De Ptisi_2
orobus	De Ptisi_2
sedes of linsed	De Ptisi_2
barlich	De Ptisi_2

date	De Ptisi_2
carica	De Ptisi_2
aristologie rotunde	De Ptisi_2
oil of violet	De Ptisi_2
rote of yreos	De Ptisi_3
fenigrec	De Ptisi_3
ysope	De Ptisi_3
aristologie	De Ptisi_3
malve	De Ptisi_3
bismalve	De Ptisi_3
capilli veneris	De Ptisi_3
barlich	De Ptisi_3
anete	De Ptisi_3
fenigrec	De Ptisi_3
liquirice	De Ptisi_3
jujube	De Ptisi_3
carica	De Ptisi_3
4 cold sedes	De Ptisi_3
sedes of bruscus	De Ptisi_3
sedes of sparage	De Ptisi_3
hony	De Ptisi_3
water	De Ptisi_3
tyriaca	De Ptisi_4
rubee trociscata	De Ptisi_4
apium	De Ptisi_4
crocus	De Ptisi_4
bole armoniac	De Ptisi_5
terra sigillata	De Ptisi_5
candi penidiarum	De Ptisi_5
draganganti	De Ptisi_5
sedes of coriandre	De Ptisi_5
liquirice	De Ptisi_5
uva passe	De Ptisi_5

jujube	De Ptisi_5
ydromel	De Ptisi_5
mylke	De Ptisi_5
mirre	De Ptisi_6
storace liquide	De Ptisi_6
galbanum	De Ptisi_6
arsenic	De Ptisi_6
butere	De Ptisi_6
ypoquistados	De Ptisi_7
acacia	De Ptisi_7
sandali	De Ptisi_7
spodium	De Ptisi_7
cacabre	De Ptisi_7
dragaganti	De Ptisi_7
gummi arabic assi	De Ptisi_7
mirtille	De Ptisi_7
sumac	De Ptisi_7
liquirice	De Ptisi_7
carica	De Ptisi_7
uva passe	De Ptisi_7
pine	De Ptisi_7
pistace	De Ptisi_7
sirup of mirtille	De Ptisi_7
mylke	De Ptisi_7
olibanum	De Spitto Sanguinis_1
sedes of portulaca	De Spitto Sanguinis_1
sedes of malve	De Spitto Sanguinis_1
sanguis draconis	De Spitto Sanguinis_1
opium	De Spitto Sanguinis_1
crocus	De Spitto Sanguinis_1
muscilage of dragaganti	De Spitto Sanguinis_1
juse of caul	De Difficultate Anelitus_1
crocus	De Difficultate Anelitus_1

sugre	De Difficultate Anelitus_1
hony	De Difficultate Anelitus_1
rote of bruscus	De Pleuresi_1
sparage	De Pleuresi_1
graminis	De Pleuresi_1
endive	De Pleuresi_1
scariol	De Pleuresi_1
capilli veneris recent	De Pleuresi_1
barlich	De Pleuresi_1
4 cold sedes	De Pleuresi_1
sedes of whit papaver	De Pleuresi_1
liquirice	De Pleuresi_1
cubebe	De Pleuresi_1
hony	De Pleuresi_1
sugre	De Pleuresi_1
rote of malve	De Pleuresi_2
orobus	De Pleuresi_2
fenigrec	De Pleuresi_2
sedes of linsed	De Pleuresi_2
nasturcium	De Pleuresi_2
sedes of fenel	De Pleuresi_2
swete almaundes	De Pleuresi_2
dragaganti	De Pleuresi_2
gummi arabic	De Pleuresi_2
cubebe	De Pleuresi_2
liquirice	De Pleuresi_2
oil of sisamie	De Pleuresi_2
water	De Pleuresi_2
rote of yreos	De Pleuresi_3
ysope	De Pleuresi_3
lilie	De Pleuresi_3
carica	De Pleuresi_3
date	De Pleuresi_3

uva passe	De Pleuresi_3
jujube	De Pleuresi_3
liquirice	De Pleuresi_3
dragaganti	De Pleuresi_3
gummi arabic	De Pleuresi_3
4 cold sedes	De Pleuresi_3
capilli veneris recent	De Pleuresi_3
hony	De Pleuresi_3
sugre	De Pleuresi_3
orobus	De Pleuresi_3
fenigrec	De Pleuresi_3
linsed	De Pleuresi_3
culver dunke	De Pleuresi_3
opoponac	De Pleuresi_3
ris	De Apostemate Mamillarum_1
apium	De Apostemate Mamillarum_1
wyn	De Apostemate Mamillarum_1
lilie	De Apostemate Mamillarum_2
apium	De Apostemate Mamillarum_2
fenigrec	De Apostemate Mamillarum_2
sedes of linsed	De Apostemate Mamillarum_2
farina of amidum	De Apostemate Mamillarum_2
mirre	De Apostemate Mamillarum_2
crocus	De Apostemate Mamillarum_2
hony	De Apostemate Mamillarum_2
4 cold sedes major and minor	De Difficultate Transgluciendi_1
sandali	De Difficultate Transgluciendi_1
liquirice	De Difficultate Transgluciendi_1
muscilage of psillie	De Difficultate Transgluciendi_1
water of rosis	De Difficultate Transgluciendi_1
4 cold sedes	De Difficultate Transgluciendi_2
sedes of malve	De Difficultate Transgluciendi_2
sedes of portulaca	De Difficultate Transgluciendi_2

sedes of linsed	De Difficultate Transgluciendi_2
fenigrec	De Difficultate Transgluciendi_2
muscilage of psillie	De Difficultate Transgluciendi_2
galbanum	De Difficultate Transgluciendi_3_a
armoniac	De Difficultate Transgluciendi_3_a
bdellium	De Difficultate Transgluciendi_3_a
oil of laurel	De Difficultate Transgluciendi_3_a
wex	De Difficultate Transgluciendi_3_a
piretre	De Difficultate Transgluciendi_3_b
rote of aristologie	De Difficultate Transgluciendi_3_b
yreos	De Difficultate Transgluciendi_3_b
yreos	De Difficultate Transgluciendi_4
lilie	De Difficultate Transgluciendi_4
ysope	De Difficultate Transgluciendi_4
carica	De Difficultate Transgluciendi_4
uva passe	De Difficultate Transgluciendi_4
fenel	De Difficultate Transgluciendi_4
hony	De Difficultate Transgluciendi_4
ysope	De Difficultate Transgluciendi_5
absinthium	De Difficultate Transgluciendi_5
liquirice	De Difficultate Transgluciendi_5
uva passe	De Difficultate Transgluciendi_5
jujube	De Difficultate Transgluciendi_5
dragaganti	De Difficultate Transgluciendi_5
gummi arabic	De Difficultate Transgluciendi_5
mirtille	De Difficultate Transgluciendi_5
sedes of citonie	De Difficultate Transgluciendi_5
hony	De Difficultate Transgluciendi_5
sarcocolla	De Difficultate Transgluciendi_6
spodium	De Difficultate Transgluciendi_6
cacabre	De Difficultate Transgluciendi_6
dragaganti	De Difficultate Transgluciendi_6
gummi arabic	De Difficultate Transgluciendi_6

amidum	De Difficultate Transgluciendi_6
hony	De Difficultate Transgluciendi_6
potio muscata	De Debelitate Appetitus_1
diamente	De Debelitate Appetitus_1
diambra	De Debelitate Appetitus_1
diatrion pipereon	De Debelitate Appetitus_2
diacitoniten	De Debelitate Appetitus_2
diagalanga	De Debelitate Appetitus_2
diamente	De Debelitate Appetitus_2
diambra	De Debelitate Appetitus_2
absinthium	De Debelitate Appetitus_2
mente	De Debelitate Appetitus_2
nux muscata	De Debelitate Appetitus_2
nucis of cipresse	De Debelitate Appetitus_2
oil of laurel	De Debelitate Appetitus_2
triasandali	De Debelitate Appetitus_3
sugre of rosis	De Debelitate Appetitus_3
carnium citoniorum conditorum cum sugre	De Debelitate Appetitus_3
dragaganti frigidi	De Debelitate Appetitus_3
rosis	De Debelitate Appetitus_3
sandali	De Debelitate Appetitus_3
citonie	De Debelitate Appetitus_3
acetositas citri	De Debelitate Appetitus_3
oil of rosis	De Debelitate Appetitus_3
diacitoniten	De Debelitate Appetitus_4
potio muscata	De Debelitate Appetitus_4
conserve citrangulorum	De Debelitate Appetitus_4
absinthium	De Indigestione Stomachi vel Dolore et Debelitate et Mala Complexione et Similibus_1_a
endive	De Indigestione Stomachi vel Dolore et Debelitate et Mala Complexione et Similibus_1_a
scariol	De Indigestione Stomachi vel Dolore et Debelitate et Mala Complexione et Similibus_1_a

lactuca	De Indigestione Stomachi vel Dolore et Debelitate et Mala Complexione et Similibus_1_a
4 cold sedes	De Indigestione Stomachi vel Dolore et Debelitate et Mala Complexione et Similibus_1_a
dragaganti	De Indigestione Stomachi vel Dolore et Debelitate et Mala Complexione et Similibus_1_a
liquirice	De Indigestione Stomachi vel Dolore et Debelitate et Mala Complexione et Similibus_1_a
barlich	De Indigestione Stomachi vel Dolore et Debelitate et Mala Complexione et Similibus_1_a
prune	De Indigestione Stomachi vel Dolore et Debelitate et Mala Complexione et Similibus_1_a
sugre	De Indigestione Stomachi vel Dolore et Debelitate et Mala Complexione et Similibus_1_a
water of barlich	De Indigestione Stomachi vel Dolore et Debelitate et Mala Complexione et Similibus_1_a
cassiafistula	De Indigestione Stomachi vel Dolore et Debelitate et Mala Complexione et Similibus_1_a
absinthium	De Indigestione Stomachi vel Dolore et Debelitate et Mala Complexione et Similibus_1_a
endive	De Indigestione Stomachi vel Dolore et Debelitate et Mala Complexione et Similibus_1_a
scariol	De Indigestione Stomachi vel Dolore et Debelitate et Mala Complexione et Similibus_1_a
malve	De Indigestione Stomachi vel Dolore et Debelitate et Mala Complexione et Similibus_1_a
rosis	De Indigestione Stomachi vel Dolore et Debelitate et Mala Complexione et Similibus_1_a
mastic	De Indigestione Stomachi vel Dolore et Debelitate et Mala Complexione et Similibus_1_a
farina of barlich	De Indigestione Stomachi vel Dolore et Debelitate et Mala Complexione et Similibus_1_a

juse of apium	De Indigestione Stomachi vel Dolore et Debelitate et Mala Complexione et Similibus_2_b
henne grece	De Indigestione Stomachi vel Dolore et Debelitate et Mala Complexione et Similibus_2_b
dokys grece	De Indigestione Stomachi vel Dolore et Debelitate et Mala Complexione et Similibus_2_b
spicenardi	De Indigestione Stomachi vel Dolore et Debelitate et Mala Complexione et Similibus_2_c
bdellium	De Indigestione Stomachi vel Dolore et Debelitate et Mala Complexione et Similibus_2_c
date	De Indigestione Stomachi vel Dolore et Debelitate et Mala Complexione et Similibus_2_c
absinthium	De Indigestione Stomachi vel Dolore et Debelitate et Mala Complexione et Similibus_3
croppes of fenel	De Indigestione Stomachi vel Dolore et Debelitate et Mala Complexione et Similibus_3
ysope	De Indigestione Stomachi vel Dolore et Debelitate et Mala Complexione et Similibus_3
capilli veneris	De Indigestione Stomachi vel Dolore et Debelitate et Mala Complexione et Similibus_3
mastic	De Indigestione Stomachi vel Dolore et Debelitate et Mala Complexione et Similibus_3
olibanum	De Indigestione Stomachi vel Dolore et Debelitate et Mala Complexione et Similibus_3
spicenardi	De Indigestione Stomachi vel Dolore et Debelitate et Mala Complexione et Similibus_3
rosis	De Indigestione Stomachi vel Dolore et Debelitate et Mala Complexione et Similibus_3
mel roset	De Indigestione Stomachi vel Dolore et Debelitate et Mala Complexione et Similibus_3
sugre	De Indigestione Stomachi vel Dolore et Debelitate et Mala Complexione et Similibus_3

yerapigra	De Indigestione Stomachi vel Dolore et Debelitate et Mala Complexione et Similibus_3
oxisacre	De Indigestione Stomachi vel Dolore et Debelitate et Mala Complexione et Similibus_4
ydromel	De Indigestione Stomachi vel Dolore et Debelitate et Mala Complexione et Similibus_4
citonie	De Indigestione Stomachi vel Dolore et Debelitate et Mala Complexione et Similibus_4
absinthium	De Indigestione Stomachi vel Dolore et Debelitate et Mala Complexione et Similibus_4
water of barlich	De Indigestione Stomachi vel Dolore et Debelitate et Mala Complexione et Similibus_4
gummi arabic	De Indigestione Stomachi vel Dolore et Debelitate et Mala Complexione et Similibus_5
dragaganti	De Indigestione Stomachi vel Dolore et Debelitate et Mala Complexione et Similibus_5
mastic	De Indigestione Stomachi vel Dolore et Debelitate et Mala Complexione et Similibus_5
olibanum	De Indigestione Stomachi vel Dolore et Debelitate et Mala Complexione et Similibus_5
cacabre	De Indigestione Stomachi vel Dolore et Debelitate et Mala Complexione et Similibus_5
juse of absinthium	De Indigestione Stomachi vel Dolore et Debelitate et Mala Complexione et Similibus_5
floures of borage	De Indigestione Stomachi vel Dolore et Debelitate et Mala Complexione et Similibus_6
floures of violet	De Indigestione Stomachi vel Dolore et Debelitate et Mala Complexione et Similibus_6
uva passe	De Indigestione Stomachi vel Dolore et Debelitate et Mala Complexione et Similibus_6
liquirice	De Indigestione Stomachi vel Dolore et Debelitate et Mala Complexione et Similibus_6

absinthium	De Indigestione Stomachi vel Dolore et Debelitate et Mala Complexione et Similibus_6
rind of mirabolan indorum	De Indigestione Stomachi vel Dolore et Debelitate et Mala Complexione et Similibus_6
aloen	De Indigestione Stomachi vel Dolore et Debelitate et Mala Complexione et Similibus_6
floures of borage	De Indigestione Stomachi vel Dolore et Debelitate et Mala Complexione et Similibus_7
sticados	De Indigestione Stomachi vel Dolore et Debelitate et Mala Complexione et Similibus_7
levys of absinthium	De Indigestione Stomachi vel Dolore et Debelitate et Mala Complexione et Similibus_7
liquirice	De Indigestione Stomachi vel Dolore et Debelitate et Mala Complexione et Similibus_7
uva passe	De Indigestione Stomachi vel Dolore et Debelitate et Mala Complexione et Similibus_7
mirabolan indorum	De Indigestione Stomachi vel Dolore et Debelitate et Mala Complexione et Similibus_7
lapidis armenici nouies abluti	De Indigestione Stomachi vel Dolore et Debelitate et Mala Complexione et Similibus_7
floures of sticados	De Indigestione Stomachi vel Dolore et Debelitate et Mala Complexione et Similibus_8
polipodi	De Indigestione Stomachi vel Dolore et Debelitate et Mala Complexione et Similibus_8
anis	De Indigestione Stomachi vel Dolore et Debelitate et Mala Complexione et Similibus_8
fenel	De Indigestione Stomachi vel Dolore et Debelitate et Mala Complexione et Similibus_8
liquirice	De Indigestione Stomachi vel Dolore et Debelitate et Mala Complexione et Similibus_8
uva passe	De Indigestione Stomachi vel Dolore et Debelitate et Mala Complexione et Similibus_8

levys of absinthium	De Indigestione Stomachi vel Dolore et Debelitate et Mala Complexione et Similibus_8
rind of mirabolan kebulorum	De Indigestione Stomachi vel Dolore et Debelitate et Mala Complexione et Similibus_8
turbith	De Indigestione Stomachi vel Dolore et Debelitate et Mala Complexione et Similibus_8
juse of mente	De Indigestione Stomachi vel Dolore et Debelitate et Mala Complexione et Similibus_9
juse of absinthium	De Indigestione Stomachi vel Dolore et Debelitate et Mala Complexione et Similibus_9
ei yolk	De Indigestione Stomachi vel Dolore et Debelitate et Mala Complexione et Similibus_9
coriandre	De Indigestione Stomachi vel Dolore et Debelitate et Mala Complexione et Similibus_10
levys of mente	De Indigestione Stomachi vel Dolore et Debelitate et Mala Complexione et Similibus_10
levys of absinthium	De Indigestione Stomachi vel Dolore et Debelitate et Mala Complexione et Similibus_10
gariofilum	De Indigestione Stomachi vel Dolore et Debelitate et Mala Complexione et Similibus_10
lignum aloes	De Indigestione Stomachi vel Dolore et Debelitate et Mala Complexione et Similibus_10
hony	De Indigestione Stomachi vel Dolore et Debelitate et Mala Complexione et Similibus_10
mente	De Indigestione Stomachi vel Dolore et Debelitate et Mala Complexione et Similibus_11
absinthium	De Indigestione Stomachi vel Dolore et Debelitate et Mala Complexione et Similibus_11
cost	De Indigestione Stomachi vel Dolore et Debelitate et Mala Complexione et Similibus_11
fenel	De Indigestione Stomachi vel Dolore et Debelitate et Mala Complexione et Similibus_11

spicenardi	De Indigestione Stomachi vel Dolore et Debelitate et Mala Complexione et Similibus_11
squinanti	De Indigestione Stomachi vel Dolore et Debelitate et Mala Complexione et Similibus_11
spice celtice	De Indigestione Stomachi vel Dolore et Debelitate et Mala Complexione et Similibus_11
calami aromatici	De Indigestione Stomachi vel Dolore et Debelitate et Mala Complexione et Similibus_11
lignum aloes	De Indigestione Stomachi vel Dolore et Debelitate et Mala Complexione et Similibus_11
oil of laurel	De Indigestione Stomachi vel Dolore et Debelitate et Mala Complexione et Similibus_11
new wex	De Indigestione Stomachi vel Dolore et Debelitate et Mala Complexione et Similibus_11
mastic	De Indigestione Stomachi vel Dolore et Debelitate et Mala Complexione et Similibus_12
spicenardi	De Indigestione Stomachi vel Dolore et Debelitate et Mala Complexione et Similibus_12
oil	De Indigestione Stomachi vel Dolore et Debelitate et Mala Complexione et Similibus_12
pitch	De Indigestione Stomachi vel Dolore et Debelitate et Mala Complexione et Similibus_12
absinthium	De Indigestione Stomachi vel Dolore et Debelitate et Mala Complexione et Similibus_13
ysope	De Indigestione Stomachi vel Dolore et Debelitate et Mala Complexione et Similibus_13
calamente	De Indigestione Stomachi vel Dolore et Debelitate et Mala Complexione et Similibus_13
anis	De Indigestione Stomachi vel Dolore et Debelitate et Mala Complexione et Similibus_13
fenel	De Indigestione Stomachi vel Dolore et Debelitate et Mala Complexione et Similibus_13

ameos	De Indigestione Stomachi vel Dolore et Debelitate et Mala Complexione et Similibus_13
siseleos	De Indigestione Stomachi vel Dolore et Debelitate et Mala Complexione et Similibus_13
spicenardi	De Indigestione Stomachi vel Dolore et Debelitate et Mala Complexione et Similibus_13
squinanti	De Indigestione Stomachi vel Dolore et Debelitate et Mala Complexione et Similibus_13
calami aromatici	De Indigestione Stomachi vel Dolore et Debelitate et Mala Complexione et Similibus_13
mel roset	De Indigestione Stomachi vel Dolore et Debelitate et Mala Complexione et Similibus_13
balsamum	De Indigestione Stomachi vel Dolore et Debelitate et Mala Complexione et Similibus_13
dianison	De Indigestione Stomachi vel Dolore et Debelitate et Mala Complexione et Similibus_14
diaciminum	De Indigestione Stomachi vel Dolore et Debelitate et Mala Complexione et Similibus_14
sugre of rosis	De Indigestione Stomachi vel Dolore et Debelitate et Mala Complexione et Similibus_14
rote of fenel	De Indigestione Stomachi vel Dolore et Debelitate et Mala Complexione et Similibus_15
petrosilie	De Indigestione Stomachi vel Dolore et Debelitate et Mala Complexione et Similibus_15
apium	De Indigestione Stomachi vel Dolore et Debelitate et Mala Complexione et Similibus_15
bruscus	De Indigestione Stomachi vel Dolore et Debelitate et Mala Complexione et Similibus_15
sparage	De Indigestione Stomachi vel Dolore et Debelitate et Mala Complexione et Similibus_15
graminis	De Indigestione Stomachi vel Dolore et Debelitate et Mala Complexione et Similibus_15

stronge vinegre	De Indigestione Stomachi vel Dolore et Debelitate et Mala Complexione et Similibus_15
ysope	De Indigestione Stomachi vel Dolore et Debelitate et Mala Complexione et Similibus_16
absinthium	De Indigestione Stomachi vel Dolore et Debelitate et Mala Complexione et Similibus_16
calamente	De Indigestione Stomachi vel Dolore et Debelitate et Mala Complexione et Similibus_16
pulegie	De Indigestione Stomachi vel Dolore et Debelitate et Mala Complexione et Similibus_16
serpel	De Indigestione Stomachi vel Dolore et Debelitate et Mala Complexione et Similibus_16
mente	De Indigestione Stomachi vel Dolore et Debelitate et Mala Complexione et Similibus_16
salvie	De Indigestione Stomachi vel Dolore et Debelitate et Mala Complexione et Similibus_16
basilicon	De Indigestione Stomachi vel Dolore et Debelitate et Mala Complexione et Similibus_16
majorane	De Indigestione Stomachi vel Dolore et Debelitate et Mala Complexione et Similibus_16
hony	De Indigestione Stomachi vel Dolore et Debelitate et Mala Complexione et Similibus_16
yerapigra	De Indigestione Stomachi vel Dolore et Debelitate et Mala Complexione et Similibus_17
aloen	De Indigestione Stomachi vel Dolore et Debelitate et Mala Complexione et Similibus_17
turbith	De Indigestione Stomachi vel Dolore et Debelitate et Mala Complexione et Similibus_17
esula	De Indigestione Stomachi vel Dolore et Debelitate et Mala Complexione et Similibus_17
anis	De Indigestione Stomachi vel Dolore et Debelitate et Mala Complexione et Similibus_17

cinamome	De Indigestione Stomachi vel Dolore et Debelitate et Mala Complexione et Similibus_17
mastic	De Indigestione Stomachi vel Dolore et Debelitate et Mala Complexione et Similibus_17
juse of absinthium	De Indigestione Stomachi vel Dolore et Debelitate et Mala Complexione et Similibus_17
diatrion pipereon	De Indigestione Stomachi vel Dolore et Debelitate et Mala Complexione et Similibus_18
zinziberis	De Indigestione Stomachi vel Dolore et Debelitate et Mala Complexione et Similibus_18
cinamome	De Indigestione Stomachi vel Dolore et Debelitate et Mala Complexione et Similibus_18
gariofilum	De Indigestione Stomachi vel Dolore et Debelitate et Mala Complexione et Similibus_18
ciperus	De Indigestione Stomachi vel Dolore et Debelitate et Mala Complexione et Similibus_18
zedoaria	De Indigestione Stomachi vel Dolore et Debelitate et Mala Complexione et Similibus_18
mastic	De Indigestione Stomachi vel Dolore et Debelitate et Mala Complexione et Similibus_18
cardamome	De Indigestione Stomachi vel Dolore et Debelitate et Mala Complexione et Similibus_18
nux muscata	De Indigestione Stomachi vel Dolore et Debelitate et Mala Complexione et Similibus_18
anis	De Indigestione Stomachi vel Dolore et Debelitate et Mala Complexione et Similibus_18
fenel	De Indigestione Stomachi vel Dolore et Debelitate et Mala Complexione et Similibus_18
comin	De Indigestione Stomachi vel Dolore et Debelitate et Mala Complexione et Similibus_18
ameos	De Indigestione Stomachi vel Dolore et Debelitate et Mala Complexione et Similibus_18

siseleos	De Indigestione Stomachi vel Dolore et Debelitate et Mala Complexione et Similibus_18
lignum aloes	De Indigestione Stomachi vel Dolore et Debelitate et Mala Complexione et Similibus_18
carpobalsamum	De Indigestione Stomachi vel Dolore et Debelitate et Mala Complexione et Similibus_18
xilobalsamum	De Indigestione Stomachi vel Dolore et Debelitate et Mala Complexione et Similibus_18
spicenardi	De Indigestione Stomachi vel Dolore et Debelitate et Mala Complexione et Similibus_18
spice celtice	De Indigestione Stomachi vel Dolore et Debelitate et Mala Complexione et Similibus_18
calami aromatici	De Indigestione Stomachi vel Dolore et Debelitate et Mala Complexione et Similibus_18
poudre of mente	De Indigestione Stomachi vel Dolore et Debelitate et Mala Complexione et Similibus_18
crocus	De Indigestione Stomachi vel Dolore et Debelitate et Mala Complexione et Similibus_18
sugre	De Indigestione Stomachi vel Dolore et Debelitate et Mala Complexione et Similibus_18
diamente	De Indigestione Stomachi vel Dolore et Debelitate et Mala Complexione et Similibus_19
diatrion pipereon	De Indigestione Stomachi vel Dolore et Debelitate et Mala Complexione et Similibus_19
pliris cum musco	De Indigestione Stomachi vel Dolore et Debelitate et Mala Complexione et Similibus_20
dianthos cum musco	De Indigestione Stomachi vel Dolore et Debelitate et Mala Complexione et Similibus_20
diambra	De Indigestione Stomachi vel Dolore et Debelitate et Mala Complexione et Similibus_20
leticia galen	De Indigestione Stomachi vel Dolore et Debelitate et Mala Complexione et Similibus_20

diamente	De Indigestione Stomachi vel Dolore et Debelitate et Mala Complexione et Similibus_20
diatrion pipereon	De Indigestione Stomachi vel Dolore et Debelitate et Mala Complexione et Similibus_20
diacitoniten	De Indigestione Stomachi vel Dolore et Debelitate et Mala Complexione et Similibus_21
diatrion pipereon	De Indigestione Stomachi vel Dolore et Debelitate et Mala Complexione et Similibus_21
carnium citoniorum	De Indigestione Stomachi vel Dolore et Debelitate et Mala Complexione et Similibus_21
diamente	De Indigestione Stomachi vel Dolore et Debelitate et Mala Complexione et Similibus_21
dragaganti frigidi	De Indigestione Stomachi vel Dolore et Debelitate et Mala Complexione et Similibus_22
diabuglosse	De Indigestione Stomachi vel Dolore et Debelitate et Mala Complexione et Similibus_22
sugre of rosis	De Indigestione Stomachi vel Dolore et Debelitate et Mala Complexione et Similibus_22
diarodon abbatis	De Indigestione Stomachi vel Dolore et Debelitate et Mala Complexione et Similibus_22
cupula glandium	De Corrupcione Appetitus_1
lignum aloes	De Corrupcione Appetitus_1
anis	De Corrupcione Appetitus_1
fenel	De Corrupcione Appetitus_1
spicenardi	De Corrupcione Appetitus_1
nux muscata	De Corrupcione Appetitus_1
gariofilum	De Corrupcione Appetitus_1
mirtille	De Corrupcione Appetitus_1
sugre	De Corrupcione Appetitus_1
conserve citrangulorum	De Corrupcione Appetitus_1
4 cold sedes	De Siti_1
sedes of endive	De Siti_1
sedes of scariol	De Siti_1

sedes of lactuca	De Siti_1
sedes of portulaca	De Siti_1
sedes of whit papaver	De Siti_1
muscilage of psillie	De Siti_1
dragaganti	De Siti_1
spicenardi	De Singultu_1
anis	De Singultu_1
lignum aloes	De Singultu_1
crocus	De Singultu_1
levys of mente	De Singultu_1
levys of absinthium	De Singultu_1
squinanti	De Singultu_1
calami aromatici	De Singultu_1
fenel	De Singultu_1
cost	De Singultu_1
ambra grisie	De Singultu_1
gariofilum	De Singultu_1
aloen cicotrini	De Singultu_1
coriandre	De Singultu_1
sedes of whit papaver	De Singultu_1
cassialignea	De Singultu_1
conserve citrangulorum	De Singultu_1
sugre	De Singultu_1
sirup of rosis	De Singultu_2
oxisacre	De Singultu_2
sirup of acetose	De Singultu_2
levys of absinthium	De Singultu_2
yerapigra	De Singultu_3
trifera sarasenica	De Singultu_3
reubarbe	De Singultu_3
sugre of rosis	De Abhominacione et Nausea et Subvercione Anie_1
triasandali	De Abhominacione et Nausea et Subvercione Anie_1
diarodon abbatis	De Abhominacione et Nausea et Subvercione Anie_1

oximel diuretik	De Abhominacione et Nausea et Subvercione Anie_2
oximel squillitik	De Abhominacione et Nausea et Subvercione Anie_2
absinthium	De Abhominacione et Nausea et Subvercione Anie_2
water	De Abhominacione et Nausea et Subvercione Anie_2
mastic	De Abhominacione et Nausea et Subvercione Anie_3
cinamome	De Abhominacione et Nausea et Subvercione Anie_3
lignum aloes	De Abhominacione et Nausea et Subvercione Anie_3
zedoaria	De Abhominacione et Nausea et Subvercione Anie_3
ciperus	De Abhominacione et Nausea et Subvercione Anie_3
gariofilum	De Abhominacione et Nausea et Subvercione Anie_3
anis	De Abhominacione et Nausea et Subvercione Anie_3
fenel	De Abhominacione et Nausea et Subvercione Anie_3
levys of absinthium	De Abhominacione et Nausea et Subvercione Anie_3
brede	De Abhominacione et Nausea et Subvercione Anie_3
wyn	De Abhominacione et Nausea et Subvercione Anie_3
mirre	De Vomitu Sanguinis_1
sarcocolla	De Vomitu Sanguinis_1
mumie	De Vomitu Sanguinis_1
mastic	De Vomitu Sanguinis_1
olibanum	De Vomitu Sanguinis_1
wyn	De Vomitu Sanguinis_1
rind of citrus	De Vomitu Sanguinis_2
lignum aloes	De Vomitu Sanguinis_2
mastic	De Vomitu Sanguinis_2
coriandre	De Vomitu Sanguinis_2
sedes of portulaca	De Vomitu Sanguinis_2
sandali	De Vomitu Sanguinis_2
muscatelini	De Vomitu Sanguinis_2
diacodion	De Vomitu Sanguinis_3
diapapaver	De Vomitu Sanguinis_3
triasandali	De Vomitu Sanguinis_3
sugre of rosis	De Vomitu Sanguinis_3
carnium citoniorum cum sugre sine	De Vomitu Sanguinis_3

bole armoniac	De Lienteria_1
terra sigillata	De Lienteria_1
coral	De Lienteria_1
sandali	De Lienteria_1
sumac	De Lienteria_1
berberis	De Lienteria_1
sedes of citonie	De Lienteria_1
psidia	De Lienteria_1
balaustia	De Lienteria_1
galle	De Lienteria_1
ypoquistados	De Lienteria_1
mirtille	De Lienteria_1
rosis drie	De Lienteria_1
sanguis draconis	De Lienteria_1
juse of plantago	De Lienteria_1
juse of absinthium	De Lienteria_1
mylke	De Lienteria_1
anis	De Lienteria_2
comin	De Lienteria_2
nasturcium assi	De Lienteria_2
mastic	De Lienteria_2
olibanum	De Lienteria_2
lapdanum	De Lienteria_2
storace calamite	De Lienteria_2
ambra grisie	De Lienteria_2
mirre	De Lienteria_2
nucis of cipresse	De Lienteria_2
levys of cipresse	De Lienteria_2
juse of mente	De Lienteria_2
juse of absinthium	De Lienteria_2
mylke	De Lienteria_2
mirabolan assi	De Dissinteria_1
bole armoniac	De Dissinteria_1

poudre of cacabre	De Dissinteria_1
muscilage of dragaganti	De Dissinteria_1
gummi arabic	De Dissinteria_1
juse of plantago	De Dissinteria_1
mylke	De Dissinteria_1
juse of portulaca	De Dissinteria_2
juse of plantago	De Dissinteria_2
juse of virge pastoris	De Dissinteria_2
sumac	De Dissinteria_2
mirtille	De Dissinteria_2
cupula glandium	De Dissinteria_2
acacia	De Dissinteria_2
carte combuste	De Dissinteria_2
alum ust	De Dissinteria_2
cepa renum caprarum	De Dissinteria_2
ei yolk	De Dissinteria_2
water	De Dissinteria_2
diaolibanum	De Dissinteria_3
diamente	De Dissinteria_3
diacitoniten	De Dissinteria_3
diacodion	De Dissinteria_3
mirre	De Dissinteria_4
castor	De Dissinteria_4
olibanum	De Dissinteria_4
storace calamite	De Dissinteria_4
opium	De Dissinteria_4
dragaganti	De Dissinteria_4
gummi arabic	De Dissinteria_4
ydromel	De Dissinteria_4
diadragaganti frigidi	De Dissinteria_5
electuari resumptivi	De Dissinteria_5
triasandali	De Dissinteria_5
sugre of rosis	De Dissinteria_5

mirre	De Dissinteria_6
sarcocolla	De Dissinteria_6
cinamome	De Dissinteria_6
olibanum	De Dissinteria_6
juse of plantago	De Dissinteria_6
water of barlich	De Dissinteria_6
whit wyn	De Dissinteria_6
sugre of rosis	Tractatus de Diarria_1
triasandali	Tractatus de Diarria_1
carnium citoniorum conditorum cum sugre	Tractatus de Diarria_1
fenigrec	De Tenasmone_1
sedes of linsed	De Tenasmone_1
malve	De Tenasmone_1
bismalve	De Tenasmone_1
mellilote	De Tenasmone_1
camomille	De Tenasmone_1
caul	De Tenasmone_1
water	De Tenasmone_1
linsed assi	De Fluxu Ventris in Generali_1
comin assi	De Fluxu Ventris in Generali_1
date	De Fluxu Ventris in Generali_1
nux muscata	De Fluxu Ventris in Generali_1
pomes	De Fluxu Ventris in Generali_1
cipresse	De Fluxu Ventris in Generali_1
mastic	De Fluxu Ventris in Generali_1
olibanum	De Fluxu Ventris in Generali_1
lapdanum	De Fluxu Ventris in Generali_1
mente drie	De Fluxu Ventris in Generali_1
ruta	De Fluxu Ventris in Generali_1
asarabacca	De Fluxu Ventris in Generali_1
oil of rosis	De Fluxu Ventris in Generali_1
wex	De Fluxu Ventris in Generali_1
pitch	De Fluxu Ventris in Generali_1

triasandali	De Fluxu Ventris in Generali_2
diacodion	De Fluxu Ventris in Generali_2
diadragaganti frigidi	De Fluxu Ventris in Generali_2
diatrion pipereon	De Fluxu Ventris in Generali_3
diambra	De Fluxu Ventris in Generali_3
diamente	De Fluxu Ventris in Generali_3
anis	De Fluxu Ventris in Generali_4
comin	De Fluxu Ventris in Generali_4
fenel	De Fluxu Ventris in Generali_4
sedes of ruta	De Fluxu Ventris in Generali_4
ameos	De Fluxu Ventris in Generali_4
zinziberis	De Fluxu Ventris in Generali_4
stronge vinegre	De Fluxu Ventris in Generali_4
gariofilum	De Fluxu Ventris in Generali_5
piperis long	De Fluxu Ventris in Generali_5
nux muscata	De Fluxu Ventris in Generali_5
pomes	De Fluxu Ventris in Generali_5
levys of cipresse	De Fluxu Ventris in Generali_5
storace calamite	De Fluxu Ventris in Generali_5
lignum aloes	De Fluxu Ventris in Generali_5
blatta bizancie	De Fluxu Ventris in Generali_5
cardamome	De Fluxu Ventris in Generali_5
calami aromatici	De Fluxu Ventris in Generali_5
ciperus	De Fluxu Ventris in Generali_5
musco	De Fluxu Ventris in Generali_5
ambra grisie	De Fluxu Ventris in Generali_5
sugre	De Fluxu Ventris in Generali_5
scolopendrie	De Fluxu Ventris Propter Opilacionem Epatis et Venarum Misaraicarum_1
rind of capparis	De Fluxu Ventris Propter Opilacionem Epatis et Venarum Misaraicarum_1
spicenardi	De Fluxu Ventris Propter Opilacionem Epatis et Venarum Misaraicarum_1
spice celtice	De Fluxu Ventris Propter Opilacionem Epatis et Venarum Misaraicarum_1
squinanti	De Fluxu Ventris Propter Opilacionem Epatis et Venarum Misaraicarum_1
capilli veneris	De Fluxu Ventris Propter Opilacionem Epatis et Venarum Misaraicarum_1

sedes of endive	De Fluxu Ventris Propter Opilacionem Epatis et Venarum Misaraicarum_1
sedes of scariol	De Fluxu Ventris Propter Opilacionem Epatis et Venarum Misaraicarum_1
sedes of melon	De Fluxu Ventris Propter Opilacionem Epatis et Venarum Misaraicarum_1
poli montani	De Fluxu Ventris Propter Opilacionem Epatis et Venarum Misaraicarum_1
lupine	De Fluxu Ventris Propter Opilacionem Epatis et Venarum Misaraicarum_1
sedes of fenigrec	De Fluxu Ventris Propter Opilacionem Epatis et Venarum Misaraicarum_1
absinthium	De Fluxu Ventris Propter Opilacionem Epatis et Venarum Misaraicarum_1
camedreos	De Fluxu Ventris Propter Opilacionem Epatis et Venarum Misaraicarum_1
lacca	De Fluxu Ventris Propter Opilacionem Epatis et Venarum Misaraicarum_1
armoniac	De Fluxu Ventris Propter Opilacionem Epatis et Venarum Misaraicarum_1
mastic	De Fluxu Ventris Propter Opilacionem Epatis et Venarum Misaraicarum_1
wyn	De Fluxu Ventris Propter Opilacionem Epatis et Venarum Misaraicarum_1
oil of camomille	De Fluxu Ventris Propter Opilacionem Epatis et Venarum Misaraicarum_1
wex	De Fluxu Ventris Propter Opilacionem Epatis et Venarum Misaraicarum_1
oil	De Fluxu Ventris Propter Opilacionem Epatis et Venarum Misaraicarum_1
rote of fenel	De Fluxu Ventris Propter Opilacionem Epatis et Venarum Misaraicarum_2
petrosilie	De Fluxu Ventris Propter Opilacionem Epatis et Venarum Misaraicarum_2
bruscus	De Fluxu Ventris Propter Opilacionem Epatis et Venarum Misaraicarum_2
sparage	De Fluxu Ventris Propter Opilacionem Epatis et Venarum Misaraicarum_2
graminis	De Fluxu Ventris Propter Opilacionem Epatis et Venarum Misaraicarum_2
middel rind of sambucus	De Fluxu Ventris Propter Opilacionem Epatis et Venarum Misaraicarum_2
middel rind of ebulus	De Fluxu Ventris Propter Opilacionem Epatis et Venarum Misaraicarum_2
stronge vinegre	De Fluxu Ventris Propter Opilacionem Epatis et Venarum Misaraicarum_2
capilli veneris	De Fluxu Ventris Propter Opilacionem Epatis et Venarum Misaraicarum_2
lupuli	De Fluxu Ventris Propter Opilacionem Epatis et Venarum Misaraicarum_2
scolopendrie	De Fluxu Ventris Propter Opilacionem Epatis et Venarum Misaraicarum_2
absinthium	De Fluxu Ventris Propter Opilacionem Epatis et Venarum Misaraicarum_2
centorie	De Fluxu Ventris Propter Opilacionem Epatis et Venarum Misaraicarum_2
camepitheos	De Fluxu Ventris Propter Opilacionem Epatis et Venarum Misaraicarum_2
lactuca	De Fluxu Ventris Propter Opilacionem Epatis et Venarum Misaraicarum_2
asarabacca	De Fluxu Ventris Propter Opilacionem Epatis et Venarum Misaraicarum_2
calami aromatici	De Fluxu Ventris Propter Opilacionem Epatis et Venarum Misaraicarum_2
spicenardi	De Fluxu Ventris Propter Opilacionem Epatis et Venarum Misaraicarum_2

rind of capparis	De Fluxu Ventris Propter Opilacionem Epatis et Venarum Misaraicarum_2
hony	De Fluxu Ventris Propter Opilacionem Epatis et Venarum Misaraicarum_2
sugre	De Fluxu Ventris Propter Opilacionem Epatis et Venarum Misaraicarum_2
brothe of cicer	De Fluxu Ventris Propter Opilacionem Epatis et Venarum Misaraicarum_2
cacabre	De Fluxu Ventris Virulentus cum Excoriacione_1
coral red, whit	De Fluxu Ventris Virulentus cum Excoriacione_1
sandali	De Fluxu Ventris Virulentus cum Excoriacione_1
sedes of canabis	De Fluxu Ventris Virulentus cum Excoriacione_1
sedes of acetose	De Fluxu Ventris Virulentus cum Excoriacione_1
alum	De Fluxu Ventris Virulentus cum Excoriacione_1
margarite	De Fluxu Ventris Virulentus cum Excoriacione_1
mel roset	De Fluxu Ventris Virulentus cum Excoriacione_1
juse of plantago	De Fluxu Ventris Virulentus cum Excoriacione_2
juse of barbe yrcine	De Fluxu Ventris Virulentus cum Excoriacione_2
acacia	De Fluxu Ventris Virulentus cum Excoriacione_2
galle	De Fluxu Ventris Virulentus cum Excoriacione_2
bole armoniac	De Fluxu Ventris Virulentus cum Excoriacione_2
balaustia	De Fluxu Ventris Virulentus cum Excoriacione_2
sumac	De Fluxu Ventris Virulentus cum Excoriacione_2
cepa renum caprarum	De Fluxu Ventris Virulentus cum Excoriacione_2
water	De Fluxu Ventris Virulentus cum Excoriacione_2
zedoaria	De Fluxu Ventris Propter Splenem_1
cacabre	De Fluxu Ventris Propter Splenem_1
spodium	De Fluxu Ventris Propter Splenem_1
asarabacca	De Fluxu Ventris Propter Splenem_1
been rubea	De Fluxu Ventris Propter Splenem_1
ruta	De Fluxu Ventris Propter Splenem_1
ris	De Fluxu Ventris Propter Splenem_1
hony	De Fluxu Ventris Propter Splenem_1
malve	De Yliaca Pascione_1_a
bismalve	De Yliaca Pascione_1_a
mercurial	De Yliaca Pascione_1_a
violet	De Yliaca Pascione_1_a

volubilis medie	De Yliaca Pascione_1_a
floures of borage	De Yliaca Pascione_1_a
floures of violet	De Yliaca Pascione_1_a
prune	De Yliaca Pascione_1_a
oil of violet	De Yliaca Pascione_1_a
hony	De Yliaca Pascione_1_a
cassiafistula	De Yliaca Pascione_1_a
oil of almaundes	De Yliaca Pascione_1_b
mastic	De Yliaca Pascione_1_b
diagridium	De Yliaca Pascione_1_b
ruta	De Yliaca Pascione_2
origane	De Yliaca Pascione_2
calamente	De Yliaca Pascione_2
ysope	De Yliaca Pascione_2
rote of lilie	De Yliaca Pascione_2
anis	De Yliaca Pascione_2
fenel	De Yliaca Pascione_2
sedes of ruta	De Yliaca Pascione_2
turbith	De Yliaca Pascione_2
polipodi	De Yliaca Pascione_2
juse of bleta	De Yliaca Pascione_2
sal gemme	De Yliaca Pascione_2
hony	De Yliaca Pascione_2
oil of ruta	De Yliaca Pascione_2
yerapigra	De Yliaca Pascione_2
rote of fenel	De Colica Pascione_1
petrosilie	De Colica Pascione_1
bruscus	De Colica Pascione_1
sparage	De Colica Pascione_1
apium	De Colica Pascione_1
vinegre	De Colica Pascione_1
origane	De Colica Pascione_2
calamente	De Colica Pascione_2

ruta	De Colica Pascione_2
anis	De Colica Pascione_2
fenel	De Colica Pascione_2
sedes of ruta	De Colica Pascione_2
hony	De Colica Pascione_2
floures of borage	De Colica Pascione_3
floures of violet	De Colica Pascione_3
sticados	De Colica Pascione_3
liquirice	De Colica Pascione_3
uva passe	De Colica Pascione_3
polipodi	De Colica Pascione_3
anis	De Colica Pascione_3
turbith	De Colica Pascione_3
acacia	De Pascionibus Ani et Propter de Emoroydis_1
mastic	De Pascionibus Ani et Propter de Emoroydis_1
olibanum	De Pascionibus Ani et Propter de Emoroydis_1
sanguis draconis	De Pascionibus Ani et Propter de Emoroydis_1
tele aranee	De Pascionibus Ani et Propter de Emoroydis_1
pilorum leporis	De Pascionibus Ani et Propter de Emoroydis_1
glutini piscium	De Pascionibus Ani et Propter de Emoroydis_1
glutini carpentariorum	De Pascionibus Ani et Propter de Emoroydis_1
galle	De Pascionibus Ani et Propter de Emoroydis_1
sumac	De Pascionibus Ani et Propter de Emoroydis_1
mirtille	De Pascionibus Ani et Propter de Emoroydis_1
triasandali	De Pascionibus Ani et Propter de Emoroydis_2
diacodion	De Pascionibus Ani et Propter de Emoroydis_2
carnium citoniorum conditorum cum sugre	De Pascionibus Ani et Propter de Emoroydis_2
mirabolan assi	De Pascionibus Ani et Propter de Emoroydis_3
gummi arabic	De Pascionibus Ani et Propter de Emoroydis_3
mastic	De Pascionibus Ani et Propter de Emoroydis_3
rosis	De Pascionibus Ani et Propter de Emoroydis_3
anis	De Pascionibus Ani et Propter de Emoroydis_3
comin assi	De Pascionibus Ani et Propter de Emoroydis_3

lignum aloes	De Pascionibus Ani et Propter de Emoroydis_3
gariofilum	De Pascionibus Ani et Propter de Emoroydis_3
epithimi	De Pascionibus Ani et Propter de Emoroydis_3
sirup of mirtille	De Pascionibus Ani et Propter de Emoroydis_3
water	De Pascionibus Ani et Propter de Emoroydis_3
leke	De Pascionibus Ani et Propter de Emoroydis_4
caul	De Pascionibus Ani et Propter de Emoroydis_4
frumente	De Pascionibus Ani et Propter de Emoroydis_4
alum	De Pascionibus Ani et Propter de Emoroydis_4
calx vive	De Pascionibus Ani et Propter de Emoroydis_4
water	De Pascionibus Ani et Propter de Emoroydis_4
whit papaver	De Apostemate Ani_1
iusquiamus	De Apostemate Ani_1
portulaca	De Apostemate Ani_1
ei yolk	De Apostemate Ani_1
brede	De Apostemate Ani_1
oil of rosis	De Apostemate Ani_1
ei yolk	De Apostemate Ani_2
dragaganti	De Apostemate Ani_2
oil of rosis	De Apostemate Ani_2
sirup of rosis	De Apostemate Ani_2
litarge	De Ragadiis_1
cathimia aure	De Ragadiis_1
cathimia argent	De Ragadiis_1
ceruse lote	De Ragadiis_1
aloen	De Ragadiis_1
mastic	De Ragadiis_1
olibanum	De Ragadiis_1
ostraceorum	De Ragadiis_1
galle	De Ragadiis_1
psidia	De Ragadiis_1
balaustia	De Ragadiis_1
mumie	De Ragadiis_1

bole armoniac	De Ragadiis_1
oil of rosis	De Ragadiis_1
whit wyn	De Ragadiis_1
hony	De Ragadiis_1
medulla cervi	De Ragadiis_2
medulla vitulli	De Ragadiis_2
medulla maxilla porci	De Ragadiis_2
galline grece	De Ragadiis_2
dokys grece	De Ragadiis_2
butere sine sal	De Ragadiis_2
oil of sisamie	De Ragadiis_2
oil of almaundes	De Ragadiis_2
oil of mirtille	De Ragadiis_2
oil of papaver	De Ragadiis_2
aloen	De Ragadiis_2
litarge	De Ragadiis_2
cathimia lote	De Ragadiis_2
dragaganti	De Ragadiis_2
new wex	De Ragadiis_2
volatilis molendini	De Ragadiis_2
mirre	De Exitu Ani_1
mumie	De Exitu Ani_1
nucis of cipresse	De Exitu Ani_1
mastic	De Exitu Ani_1
olibanum	De Exitu Ani_1
galle	De Exitu Ani_1
sumac	De Exitu Ani_1
sugre of rosis	De Calida Discracia Epatis_1
triasandali	De Calida Discracia Epatis_1
dragaganti frigidi	De Calida Discracia Epatis_1
diarodon abbatis	De Calida Discracia Epatis_1
endive	De Calida Discracia Epatis_2
scariol	De Calida Discracia Epatis_2

acetose	De Calida Discracia Epatis_2
lactuca	De Calida Discracia Epatis_2
4 cold sedes	De Calida Discracia Epatis_2
floures of nenifar	De Calida Discracia Epatis_2
epatice	De Calida Discracia Epatis_2
rosis	De Calida Discracia Epatis_2
sandali	De Calida Discracia Epatis_2
muscatelini	De Calida Discracia Epatis_2
berberis	De Calida Discracia Epatis_2
sumac	De Calida Discracia Epatis_2
spodium	De Calida Discracia Epatis_2
camphor	De Calida Discracia Epatis_2
wyn of pomegarnettes	De Calida Discracia Epatis_2
whit vinegre	De Calida Discracia Epatis_2
sugre	De Calida Discracia Epatis_2
juse of cucurbite	De Calida Discracia Epatis_3
sandali	De Calida Discracia Epatis_3
juse of sempervive	De Calida Discracia Epatis_3
juse of lactuca	De Calida Discracia Epatis_3
juse of epatice	De Calida Discracia Epatis_3
farina of barlich	De Calida Discracia Epatis_3
water of rosis	De Calida Discracia Epatis_3
stronge vinegre	De Calida Discracia Epatis_3
rote of aristologie	De Discracia Frigida Epatis_1
rote of fenel	De Discracia Frigida Epatis_1
petrosilie	De Discracia Frigida Epatis_1
bruscus	De Discracia Frigida Epatis_1
sparage	De Discracia Frigida Epatis_1
graminis	De Discracia Frigida Epatis_1
apium	De Discracia Frigida Epatis_1
stronge vinegre	De Discracia Frigida Epatis_1
eupatorie	De Discracia Frigida Epatis_2
scolopendrie	De Discracia Frigida Epatis_2

asarabacca	De Discracia Frigida Epatis_2
cost	De Discracia Frigida Epatis_2
absinthium	De Discracia Frigida Epatis_2
cinamome	De Discracia Frigida Epatis_2
sumac	De Discracia Frigida Epatis_2
berberis	De Discracia Frigida Epatis_2
rosis	De Discracia Frigida Epatis_2
sandali	De Discracia Frigida Epatis_2
muscatelini	De Discracia Frigida Epatis_2
camphor	De Discracia Frigida Epatis_2
mel roset	De Discracia Frigida Epatis_2
sugre	De Discracia Frigida Epatis_2
stronge vinegre	De Discracia Frigida Epatis_2
agarik	De Discracia Frigida Epatis_3
reubarbe	De Discracia Frigida Epatis_3
aloen	De Discracia Frigida Epatis_3
turbith	De Discracia Frigida Epatis_3
lacca	De Discracia Frigida Epatis_3
spicenardi	De Discracia Frigida Epatis_3
levys of endive	De Discracia Frigida Epatis_3
mastic	De Discracia Frigida Epatis_3
levys of absinthium	De Discracia Frigida Epatis_3
levys of mente	De Discracia Frigida Epatis_3
zinziberis	De Discracia Frigida Epatis_3
oximel	De Discracia Frigida Epatis_3
rote of capparis	De Opilacione Epatis_1
fenel	De Opilacione Epatis_1
petrosilie	De Opilacione Epatis_1
yreos	De Opilacione Epatis_1
genciane	De Opilacione Epatis_1
rote of rubee majoris	De Opilacione Epatis_1
bruscus	De Opilacione Epatis_1
sparage	De Opilacione Epatis_1

graminis	De Opilacione Epatis_1
apium	De Opilacione Epatis_1
rote of aristologie	De Opilacione Epatis_1
squille	De Opilacione Epatis_1
stronge vinegre	De Opilacione Epatis_1
asarabacca	De Opilacione Epatis_1
camedreos	De Opilacione Epatis_1
camepitheos	De Opilacione Epatis_1
eupatorie	De Opilacione Epatis_1
centorie minor	De Opilacione Epatis_1
scolopendrie	De Opilacione Epatis_1
spicenardi	De Opilacione Epatis_1
squinanti	De Opilacione Epatis_1
cinamome	De Opilacione Epatis_1
calami aromatici	De Opilacione Epatis_1
cassialignea	De Opilacione Epatis_1
anis	De Opilacione Epatis_1
fenel	De Opilacione Epatis_1
lacca	De Opilacione Epatis_1
cuscute	De Opilacione Epatis_1
juse of fenel	De Opilacione Epatis_1
juse of absinthium	De Opilacione Epatis_1
sedes of red rosis	De Opilacione Epatis_1
sandali	De Opilacione Epatis_1
muscatelini	De Opilacione Epatis_1
spodium	De Opilacione Epatis_1
hony	De Opilacione Epatis_1
sugre	De Opilacione Epatis_1
vinegre	De Opilacione Epatis_1
agarik	De Opilacione Epatis_2
turbith	De Opilacione Epatis_2
polipodi	De Opilacione Epatis_2
spicenardi	De Opilacione Epatis_2

lacca	De Opilacione Epatis_2
zinziberis	De Opilacione Epatis_2
anis	De Opilacione Epatis_2
mastic	De Opilacione Epatis_2
yerapigra	De Opilacione Epatis_2
oximel squillitik	De Opilacione Epatis_2
endive	De Opilacione Epatis_3
scariol	De Opilacione Epatis_3
ortolane recent	De Opilacione Epatis_3
capilli veneris	De Opilacione Epatis_3
lactuca recent	De Opilacione Epatis_3
epatice	De Opilacione Epatis_3
scolopendrie	De Opilacione Epatis_3
4 cold sedes	De Opilacione Epatis_3
sedes of melon	De Opilacione Epatis_3
sedes of endive	De Opilacione Epatis_3
sedes of scariol	De Opilacione Epatis_3
sedes of bruscus	De Opilacione Epatis_3
sedes of sparage	De Opilacione Epatis_3
endive agreste	De Opilacione Epatis_3
scariol agreste	De Opilacione Epatis_3
rosis	De Opilacione Epatis_3
sandali	De Opilacione Epatis_3
muscatelini	De Opilacione Epatis_3
spodium	De Opilacione Epatis_3
rasure eboris	De Opilacione Epatis_3
vinegre	De Opilacione Epatis_3
sugre	De Opilacione Epatis_3
mel roset	De Opilacione Epatis_3
reubarbe	De Opilacione Epatis_4
lacca	De Opilacione Epatis_4
spicenardi	De Opilacione Epatis_4
agarik	De Opilacione Epatis_4

levys of absinthium	De Opilacione Epatis_4
cassiafistula	De Opilacione Epatis_4
tamarinde	De Opilacione Epatis_4
uva passe	De Opilacione Epatis_4
sirup of acetose	De Opilacione Epatis_4
dragaganti frigidi	De Debilitate Epatis_1
sugre of rosis	De Debilitate Epatis_1
triasandali	De Debilitate Epatis_1
diambra	De Debilitate Epatis_2
dianthos cum musco	De Debilitate Epatis_2
diacitoniten	De Debilitate Epatis_2
diarodon abbatis	De Debilitate Epatis_2
juse of portulaca	De Apostemate Epatis_1
juse of plantago	De Apostemate Epatis_1
juse of absinthium	De Apostemate Epatis_1
juse of virge pastoris	De Apostemate Epatis_1
rosis	De Apostemate Epatis_1
coriandre	De Apostemate Epatis_1
spodium	De Apostemate Epatis_1
water of rosis	De Apostemate Epatis_1
vinegre	De Apostemate Epatis_1
scariol	De Apostemate Epatis_2
epatice	De Apostemate Epatis_2
lactuca	De Apostemate Epatis_2
capilli veneris recent	De Apostemate Epatis_2
levys of absinthium	De Apostemate Epatis_2
4 cold sedes major and minor	De Apostemate Epatis_2
sedes of rosis	De Apostemate Epatis_2
spicenardi	De Apostemate Epatis_2
whit vinegre	De Apostemate Epatis_2
sugre	De Apostemate Epatis_2
rote of fenel	De Apostemate Epatis_3
rote of apium	De Apostemate Epatis_3

spicenardi	De Apostemate Epatis_3
squinanti	De Apostemate Epatis_3
juse of buglosse	De Apostemate Epatis_3
juse of absinthium	De Apostemate Epatis_3
sirup of acetose	De Apostemate Epatis_3
reubarbe	De Apostemate Epatis_4
spicenardi	De Apostemate Epatis_4
lacca	De Apostemate Epatis_4
juse of absinthium	De Apostemate Epatis_4
endive	De Apostemate Epatis_5
scariol	De Apostemate Epatis_5
barlich	De Apostemate Epatis_5
liquirice	De Apostemate Epatis_5
floures of borage	De Apostemate Epatis_5
floures of buglosse	De Apostemate Epatis_5
floures of violet	De Apostemate Epatis_5
uva passe	De Apostemate Epatis_5
jujube	De Apostemate Epatis_5
sebesten	De Apostemate Epatis_5
water	De Apostemate Epatis_5
cassiafistula	De Apostemate Epatis_5
tamarinde	De Apostemate Epatis_5
camomille	De Apostemate Epatis_6
mellilote	De Apostemate Epatis_6
asarabacca	De Apostemate Epatis_6
squinanti	De Apostemate Epatis_6
mastic	De Apostemate Epatis_6
citonie	De Apostemate Epatis_6
fenigrec	De Apostemate Epatis_6
sedes of linsed	De Apostemate Epatis_6
calami aromatici	De Apostemate Epatis_6
absinthium	De Apostemate Epatis_6
storace calamite	De Apostemate Epatis_6

rosis	De Apostemate Epatis_6
bdellium	De Apostemate Epatis_6
carpobalsamum	De Apostemate Epatis_6
oil	De Apostemate Epatis_6
rote of fenel	De Apostemate Epatis_7
petrosilie	De Apostemate Epatis_7
lilie	De Apostemate Epatis_7
genciane	De Apostemate Epatis_7
ysope	De Apostemate Epatis_7
asarabacca	De Apostemate Epatis_7
camedreos	De Apostemate Epatis_7
absinthium	De Apostemate Epatis_7
endive	De Apostemate Epatis_7
scariol	De Apostemate Epatis_7
prassium	De Apostemate Epatis_7
ruta	De Apostemate Epatis_7
scolopendrie	De Apostemate Epatis_7
cost	De Apostemate Epatis_7
juse of buglosse	De Apostemate Epatis_7
juse of fenel	De Apostemate Epatis_7
lacca	De Apostemate Epatis_7
spicenardi	De Apostemate Epatis_7
squinanti	De Apostemate Epatis_7
calami aromatici	De Apostemate Epatis_7
cassialignea	De Apostemate Epatis_7
sedes of endive	De Apostemate Epatis_7
sedes of scariol	De Apostemate Epatis_7
rosis	De Apostemate Epatis_7
uva passe	De Apostemate Epatis_7
liquirice	De Apostemate Epatis_7
spodium	De Apostemate Epatis_7
mirtille	De Apostemate Epatis_7
sandali	De Apostemate Epatis_7

muscatelini	De Apostemate Epatis_7
whit vinegre	De Apostemate Epatis_7
sugre	De Apostemate Epatis_7
hony	De Apostemate Epatis_7
pistace	De Apostemate Epatis_8
pine	De Apostemate Epatis_8
sedes of linsed	De Apostemate Epatis_8
fenigrec	De Apostemate Epatis_8
uva passe	De Apostemate Epatis_8
carica	De Apostemate Epatis_8
jujube	De Apostemate Epatis_8
sebesten	De Apostemate Epatis_8
sedes of malve	De Apostemate Epatis_8
storace calamite	De Apostemate Epatis_8
bdellium	De Apostemate Epatis_8
mastic	De Apostemate Epatis_8
olibanum	De Apostemate Epatis_8
galline grece	De Apostemate Epatis_8
agarik	De Apostemate Epatis_9
turbith	De Apostemate Epatis_9
aloen	De Apostemate Epatis_9
spicenardi	De Apostemate Epatis_9
squinanti	De Apostemate Epatis_9
sedes of lactuca	De Apostemate Epatis_9
sedes of endive	De Apostemate Epatis_9
levys of absinthium	De Apostemate Epatis_9
oximel squillitik	De Apostemate Epatis_9
spodium	De Fluxu Sanguinis Ab Apate_1
rasure eboris	De Fluxu Sanguinis Ab Apate_1
alum	De Fluxu Sanguinis Ab Apate_1
sedes of canabis	De Fluxu Sanguinis Ab Apate_1
red rosis	De Fluxu Sanguinis Ab Apate_1
margarite	De Fluxu Sanguinis Ab Apate_1

coral red, whit	De Fluxu Sanguinis Ab Apate_1
sandali	De Fluxu Sanguinis Ab Apate_1
muscatelini	De Fluxu Sanguinis Ab Apate_1
cacabre	De Fluxu Sanguinis Ab Apate_1
sanguis draconis	De Fluxu Sanguinis Ab Apate_1
ambra grisie	De Fluxu Sanguinis Ab Apate_1
sirup of mirtille	De Fluxu Sanguinis Ab Apate_1
mylke	De Fluxu Sanguinis Ab Apate_1
zinziberis	De Ydropisi_1
ciperus	De Ydropisi_1
zedoaria	De Ydropisi_1
anis	De Ydropisi_1
fenel	De Ydropisi_1
comin	De Ydropisi_1
ameos	De Ydropisi_1
siseleos	De Ydropisi_1
spicenardi	De Ydropisi_1
calami aromatici	De Ydropisi_1
coriandre	De Ydropisi_1
sandali	De Ydropisi_1
muscatelini	De Ydropisi_1
asarabacca	De Ydropisi_2
origane	De Ydropisi_2
calamente	De Ydropisi_2
anis	De Ydropisi_2
fenel	De Ydropisi_2
camomille	De Ydropisi_2
mellilote	De Ydropisi_2
anthos	De Ydropisi_2
sticados	De Ydropisi_2
absinthium	De Ydropisi_2
water	De Ydropisi_2
triacle	De Ydropisi_2

rote of aristologie	De Ydropisi_3
yreos	De Ydropisi_3
fenel	De Ydropisi_3
petrosilie	De Ydropisi_3
bruscus	De Ydropisi_3
sparage	De Ydropisi_3
graminis	De Ydropisi_3
apium	De Ydropisi_3
genciane	De Ydropisi_3
vinegre	De Ydropisi_3
asarabacca	De Ydropisi_4
betonice	De Ydropisi_4
germandre	De Ydropisi_4
sticados	De Ydropisi_4
eupatorie	De Ydropisi_4
absinthium	De Ydropisi_4
mente	De Ydropisi_4
origane	De Ydropisi_4
calamente	De Ydropisi_4
spicenardi	De Ydropisi_4
squinanti	De Ydropisi_4
calami aromatici	De Ydropisi_4
lacca	De Ydropisi_4
anis	De Ydropisi_4
fenel	De Ydropisi_4
sedes of endive	De Ydropisi_4
sedes of scariol	De Ydropisi_4
sedes of melon	De Ydropisi_4
spodium	De Ydropisi_4
rasure eboris	De Ydropisi_4
sandali	De Ydropisi_4
muscatelini	De Ydropisi_4
liquirice	De Ydropisi_4

uva passe	De Ydropisi_4
jujube	De Ydropisi_4
vinegre	De Ydropisi_4
mel roset	De Ydropisi_4
sugre	De Ydropisi_4
euforbia	De Ydropisi_5
pulpe coloquintide	De Ydropisi_5
agarik	De Ydropisi_5
lacca	De Ydropisi_5
spicenardi	De Ydropisi_5
bdellium	De Ydropisi_5
sedes of endive	De Ydropisi_5
sedes of scariol	De Ydropisi_5
mastic	De Ydropisi_5
juse of yreos	De Ydropisi_5
juse of absinthium	De Ydropisi_5
juse of cucumeris asinini	De Ydropisi_5
carnium citoniorum	De Ydropisi_5
oximel squillitik	De Ydropisi_5
sal gemme	De Ydropisi_6
levys of ruta	De Ydropisi_6
castor	De Ydropisi_6
euforbia	De Ydropisi_6
sedes of urtica	De Ydropisi_6
hony	De Ydropisi_6
endive	De Ydropisi_7
scariol	De Ydropisi_7
ortolane silvestris	De Ydropisi_7
lactuca	De Ydropisi_7
epatice	De Ydropisi_7
fumiterre	De Ydropisi_7
scolopendrie	De Ydropisi_7
cicoree	De Ydropisi_7

cold sedes major and minor	De Ydropisi_7
spicenardi	De Ydropisi_7
levys of absinthium	De Ydropisi_7
rote of apium	De Ydropisi_7
spodium	De Ydropisi_7
sandali	De Ydropisi_7
muscatelini	De Ydropisi_7
whit vinegre	De Ydropisi_7
sugre	De Ydropisi_7
hony	De Ydropisi_7
floures of borage	De Ydropisi_8
floures of violet	De Ydropisi_8
fumiterre	De Ydropisi_8
liquirice	De Ydropisi_8
jujube	De Ydropisi_8
levys of absinthium	De Ydropisi_8
uva passe	De Ydropisi_8
prune	De Ydropisi_8
spicenardi	De Ydropisi_8
gotys whay	De Ydropisi_8
rind of mirabolan indorum	De Ydropisi_8
cassiafistula	De Ydropisi_8
reubarbe	De Ydropisi_8
juse of cucumeris asinini	De Ydropisi_9
camomille	De Ydropisi_9
anthos	De Ydropisi_9
floures of sticados	De Ydropisi_9
4 cold sedes	De Ulceribus Renum_1
sedes of whit papaver	De Ulceribus Renum_1
sedes of malve	De Ulceribus Renum_1
sedes of bombac	De Ulceribus Renum_1
sedes of portulaca	De Ulceribus Renum_1
sedes of citonie	De Ulceribus Renum_1

mirtille	De Ulceribus Renum_1
dragaganti	De Ulceribus Renum_1
gummi arabic	De Ulceribus Renum_1
pine	De Ulceribus Renum_1
cubebe	De Ulceribus Renum_1
pistace	De Ulceribus Renum_1
candi penidiarum	De Ulceribus Renum_1
liquirice	De Ulceribus Renum_1
barlich	De Ulceribus Renum_1
muscilage of psillie	De Ulceribus Renum_1
almaundes	De Ulceribus Renum_1
bole armoniac	De Ulceribus Renum_1
sanguis draconis	De Ulceribus Renum_1
rosis	De Ulceribus Renum_1
spodium	De Ulceribus Renum_1
mirre	De Ulceribus Renum_1
ydromel	De Ulceribus Renum_1
mylke	De Ulceribus Renum_1
4 cold sedes	De Minctu Sanguinis_1
sedes of whit papaver	De Minctu Sanguinis_1
dragaganti	De Minctu Sanguinis_1
liquirice	De Minctu Sanguinis_1
mastic	De Minctu Sanguinis_1
olibanum	De Minctu Sanguinis_1
mirre	De Minctu Sanguinis_1
ambra grisie	De Minctu Sanguinis_1
cupula glandium	De Minctu Sanguinis_1
spodium	De Minctu Sanguinis_1
cacabre	De Minctu Sanguinis_1
sandali	De Minctu Sanguinis_1
muscatelini	De Minctu Sanguinis_1
sanguis draconis	De Minctu Sanguinis_1
bole armoniac	De Minctu Sanguinis_1

acacia	De Minctu Sanguinis_1
terra sigillata	De Minctu Sanguinis_1
mirtille	De Minctu Sanguinis_1
sumac	De Minctu Sanguinis_1
coriandre	De Minctu Sanguinis_1
spume maris	De Minctu Sanguinis_1
squinanti	De Minctu Sanguinis_1
juse of plantago	De Minctu Sanguinis_1
mylke	De Minctu Sanguinis_1
malve	De Lapide Renum_1
bismalve	De Lapide Renum_1
sedes of bismalve	De Lapide Renum_1
sedes of linsed	De Lapide Renum_1
carica	De Lapide Renum_1
butere recent	De Lapide Renum_1
ciner of scorpiouns	De Lapide Renum_2
cantaride	De Lapide Renum_2
sanguis hirci drie	De Lapide Renum_2
ciner of vitis	De Lapide Renum_2
ciner of caul	De Lapide Renum_2
ciner of leporis	De Lapide Renum_2
cineris aviculae, quae vocatur cauda	De Lapide Renum_2
ciner of ei	De Lapide Renum_2
lapidis judaicus	De Lapide Renum_2
lapidis inventi in felle bovis	De Lapide Renum_2
lapidis spongie	De Lapide Renum_2
piperis	De Lapide Renum_2
dauci	De Lapide Renum_2
carvi	De Lapide Renum_2
sedes of malve	De Lapide Renum_2
gummi arabic	De Lapide Renum_2
sedes of saxifragie	De Lapide Renum_2
millefolii	De Lapide Renum_2

siseleos	De Lapide Renum_2
carpobalsamum	De Lapide Renum_2
xilobalsamum	De Lapide Renum_2
spicenardi	De Lapide Renum_2
capilli veneris	De Lapide Renum_2
cold sedes	De Lapide Renum_2
mel roset	De Lapide Renum_2
cicer	De Lapide Renum_2
tribuli marini	De Lapide Renum_2
aristologie	De Lapide Renum_3
genciane	De Lapide Renum_3
rind of cipresse	De Lapide Renum_3
rote of capparis	De Lapide Renum_3
oil of bitter almaundes	De Lapide Renum_3
water	De Lapide Renum_3
scorpiouns	De Lapide Renum_3
electuarium ducis	De Lapide Renum_4
licontripon	De Lapide Renum_4
diaprunis	De Lapide Renum_4
cupula glandium	De Diabetica Pascione_1
psidia	De Diabetica Pascione_1
balaustia	De Diabetica Pascione_1
acacia	De Diabetica Pascione_1
rosis	De Diabetica Pascione_1
bole armoniac	De Diabetica Pascione_1
ypoquistados	De Diabetica Pascione_1
lapdanum	De Diabetica Pascione_1
sirup of mirtille	De Diabetica Pascione_1
mirabolan assi	De Illis Qui Ignoranter Lectos Mingunt_1
vinegre	De Illis Qui Ignoranter Lectos Mingunt_1
cupula glandium assi	De Illis Qui Ignoranter Lectos Mingunt_1
water of rosis	De Illis Qui Ignoranter Lectos Mingunt_1
nasturcium assi	De Illis Qui Ignoranter Lectos Mingunt_1

juse of agresta	De Illis Qui Ignoranter Lectos Mingunt_1
mirre	De Illis Qui Ignoranter Lectos Mingunt_1
storace calamite	De Illis Qui Ignoranter Lectos Mingunt_1
ambra grisie	De Illis Qui Ignoranter Lectos Mingunt_1
sedes of ruta	De Illis Qui Ignoranter Lectos Mingunt_1
nucis of cipresse	De Illis Qui Ignoranter Lectos Mingunt_1
mastic	De Illis Qui Ignoranter Lectos Mingunt_1
olibanum	De Illis Qui Ignoranter Lectos Mingunt_1
lapdanum	De Illis Qui Ignoranter Lectos Mingunt_1
sirup of mirtille	De Illis Qui Ignoranter Lectos Mingunt_1
levistici	De Dissuria_1
crete marine	De Dissuria_1
saxifragie	De Dissuria_1
millefolii	De Dissuria_1
tribuli marini	De Dissuria_1
campestris	De Dissuria_1
raphane	De Dissuria_1
anis	De Dissuria_1
fenel	De Dissuria_1
ameos	De Dissuria_1
siseleos	De Dissuria_1
cicer nigrum	De Dissuria_1
apium	De Dissuria_1
wyn	De Dissuria_1
asa fetida	De Paucitate Coytus_1
piretre	De Paucitate Coytus_1
stafisagre	De Paucitate Coytus_1
eruca	De Paucitate Coytus_1
oynouns	De Paucitate Coytus_1
piperis	De Paucitate Coytus_1
castor	De Paucitate Coytus_1
mirre	De Paucitate Coytus_1
storace calamite	De Paucitate Coytus_1

leonis grece	De Paucitate Coytus_1
musco	De Paucitate Coytus_1
oil of cost	De Paucitate Coytus_1
wex	De Paucitate Coytus_1
testiculos vulpis	De Paucitate Coytus_2
cerebellum passerum	De Paucitate Coytus_2
water	De Paucitate Coytus_2
floures of date	De Paucitate Coytus_2
cauda stincorum	De Paucitate Coytus_2
priapi tauri	De Paucitate Coytus_3
eruca	De Paucitate Coytus_3
piperis	De Paucitate Coytus_3
gariofilum	De Paucitate Coytus_3
citonie	De Paucitate Coytus_3
testiculos vulpis	De Paucitate Coytus_3
ei yolk	De Paucitate Coytus_3
ei yolk	De Paucitate Coytus_4
butere recent	De Paucitate Coytus_4
priapi tauri	De Paucitate Coytus_4
cicer	De Paucitate Coytus_4
ciperus	De Paucitate Coytus_4
saturion	De Paucitate Coytus_4
zinziberis	De Paucitate Coytus_4
zedoaria	De Paucitate Coytus_4
mente	De Paucitate Coytus_4
testiculos gallorum	De Paucitate Coytus_4
testiculos vulpis	De Paucitate Coytus_4
cerebellum passerum	De Paucitate Coytus_4
cerebellum columbinum	De Paucitate Coytus_4
nux indica	De Paucitate Coytus_4
pine	De Paucitate Coytus_4
pistace	De Paucitate Coytus_4
jujube	De Paucitate Coytus_4

almaundes	De Paucitate Coytus_4
sedes of malve	De Paucitate Coytus_4
sedes of mercurial	De Paucitate Coytus_4
avellane	De Paucitate Coytus_4
date	De Paucitate Coytus_4
sedes of eruca	De Paucitate Coytus_4
gariofilum	De Paucitate Coytus_4
zinziberis	De Paucitate Coytus_4
piperis nigrum	De Paucitate Coytus_4
piperis long	De Paucitate Coytus_4
lingue avis	De Paucitate Coytus_4
sedes of oynouns	De Paucitate Coytus_4
cinamome	De Paucitate Coytus_4
mylke	De Paucitate Coytus_4
cauda stincorum	De Paucitate Coytus_4
hony	De Paucitate Coytus_4
sugre	De Paucitate Coytus_4
rapum	De Paucitate Coytus_5
pastinaca	De Paucitate Coytus_5
faba	De Paucitate Coytus_5
cicer	De Paucitate Coytus_5
ris	De Paucitate Coytus_5
frumente	De Paucitate Coytus_5
ram flesche	De Paucitate Coytus_5
oynouns	De Paucitate Coytus_5
hony	De Paucitate Coytus_5
cerebellum passerum	De Paucitate Coytus_5
cinamome	De Paucitate Coytus_5
gariofilum	De Paucitate Coytus_5
ciperus	De Paucitate Coytus_5
cauda stincorum	De Paucitate Coytus_5
sugre	De Paucitate Coytus_5

Additional Recipes (without Rx)

lactuca	De Herisipula, Antrace, Carbunculo, et Sacri Ignis_unaffiliated1
portulaca	De Herisipula, Antrace, Carbunculo, et Sacri Ignis_unaffiliated1
nenifar	De Herisipula, Antrace, Carbunculo, et Sacri Ignis_unaffiliated1
levys of whit vyne	De Herisipula, Antrace, Carbunculo, et Sacri Ignis_unaffiliated1
vinegre	De Herisipula, Antrace, Carbunculo, et Sacri Ignis_unaffiliated1
plantago	De Herisipula, Antrace, Carbunculo, et Sacri Ignis_unaffiliated1
barlich	De Herisipula, Antrace, Carbunculo, et Sacri Ignis_unaffiliated1
kyne tordys	De Herisipula, Antrace, Carbunculo, et Sacri Ignis_unaffiliated2
vinegre	De Herisipula, Antrace, Carbunculo, et Sacri Ignis_unaffiliated2
hony	De Herisipula, Antrace, Carbunculo, et Sacri Ignis_unaffiliated2
olibanum	De Herisipula, Antrace, Carbunculo, et Sacri Ignis_unaffiliated2
pomegarnette	De Herisipula, Antrace, Carbunculo, et Sacri Ignis_unaffiliated3
vinegre	De Herisipula, Antrace, Carbunculo, et Sacri Ignis_unaffiliated3
bole	De Herisipula, Antrace, Carbunculo, et Sacri Ignis_unaffiliated3
ei yolk	De Herisipula, Antrace, Carbunculo, et Sacri Ignis_unaffiliated4
sal	De Herisipula, Antrace, Carbunculo, et Sacri Ignis_unaffiliated4
lactuca	De Formica Miliari_unaffiliated1
portulaca	De Formica Miliari_unaffiliated1
muscilage of psillie	De Formica Miliari_unaffiliated1
barlich	De Formica Miliari_unaffiliated2
branny brede	De Formica Miliari_unaffiliated2
plantago	De Formica Miliari_unaffiliated2
rind of pomegarnettes	De Formica Miliari_unaffiliated2
sal	De Formica Miliari_unaffiliated3
nitrum	De Formica Miliari_unaffiliated3
ruta	De Formica Miliari_unaffiliated3
cucumeris asinini	De Formica Miliari_unaffiliated3
urine of a child	De Formica Miliari_unaffiliated3
juse of plantago	De Formica Miliari_unaffiliated3
muscilage of psillie	De Vulneribus_unaffiliated1
camphor	De Vulneribus_unaffiliated1
sandali	De Vulneribus_unaffiliated1

rosis	De Vulneribus_unaffiliated1
juse of portulaca	De Vulneribus_unaffiliated1
plantago	De Vulneribus_unaffiliated1
papaver	De Vulneribus_unaffiliated1
coriandre	De Vulneribus_unaffiliated1
muscilage of psillie	De Panartico_unaffiliated1
water of rosis	De Panartico_unaffiliated1
apium	De Panartico_unaffiliated1
camphor	De Panartico_unaffiliated1
farina of barlich	De Panartico_unaffiliated1
muscilage of psillie	De Obtalmia_unaffiliated1
water of rosis	De Obtalmia_unaffiliated1
muscilage sedes of citonie	De Obtalmia_unaffiliated1
muscilage of dragaganti	De Obtalmia_unaffiliated1
ei whit	De Obtalmia_unaffiliated1
womman mylke	De Obtalmia_unaffiliated1
muscilage of dragaganti	De Obtalmia_unaffiliated2
gummi arabic	De Obtalmia_unaffiliated2
fenigrec	De Obtalmia_unaffiliated2
womman mylke	De Obtalmia_unaffiliated2
red rosis	De Obtalmia_unaffiliated3
colde whit wyn	De Obtalmia_unaffiliated3
camomille	De Obtalmia_unaffiliated3
mellilote	De Obtalmia_unaffiliated3
hoot water	De Obtalmia_unaffiliated3
water of rosis	De Obtalmia_unaffiliated4
gummi arabic	De Obtalmia_unaffiliated4
fenigrec	De Obtalmia_unaffiliated4
sedes of lactuca	De Obtalmia_unaffiliated4
malve	De Obtalmia_unaffiliated4
sarcocolla	De Obtalmia_unaffiliated4
womman mylke	De Obtalmia_unaffiliated4
spicenardi	De Obtalmia_unaffiliated4

mirtille	De Obtalmia_unaffiliated4
cinamome	De Obtalmia_unaffiliated4
aloen	De Obtalmia_unaffiliated4
castor	De Obtalmia_unaffiliated4
ei schellys	De Panniculo in Conjunctiva_unaffiliated1
stronge vinegre	De Panniculo in Conjunctiva_unaffiliated1
sarcocolla nutrite	De Panniculo in Conjunctiva_unaffiliated1
aloen	De Panniculo in Conjunctiva_unaffiliated1
zinziberis	De Panniculo in Conjunctiva_unaffiliated1
gummi arabic	De Panniculo in Conjunctiva_unaffiliated1
water of eufrasie	De Panniculo in Conjunctiva_unaffiliated1
swynes grece recent	De Panniculo in Conjunctiva_unaffiliated1
womman mylke	De Panniculo in Conjunctiva_unaffiliated1
juse of celidonie	De Panniculo in Conjunctiva_unaffiliated1
malve	De Duricie in Conjunctiva_unaffiliated1
bismalve	De Duricie in Conjunctiva_unaffiliated1
farina of barlich	De Duricie in Conjunctiva_unaffiliated1
otyn	De Duricie in Conjunctiva_unaffiliated1
water of fenel	De Pruritu in Conjunctiva_unaffiliated1
celidonie	De Pruritu in Conjunctiva_unaffiliated1
verbene	De Pruritu in Conjunctiva_unaffiliated1
eufrasie	De Pruritu in Conjunctiva_unaffiliated1
galle	De Fistula in Lacrimali_unaffiliated1
sumac	De Fistula in Lacrimali_unaffiliated1
hony	De Fistula in Lacrimali_unaffiliated1
water	De Fistula in Lacrimali_unaffiliated1
pomegarnette	De Fistula in Lacrimali_unaffiliated1
levys of ruta	De Fistula in Lacrimali_unaffiliated1
aloen	De Fistula in Lacrimali_unaffiliated2
olibanum	De Fistula in Lacrimali_unaffiliated2
sarcocolla	De Fistula in Lacrimali_unaffiliated2
sumac	De Fistula in Lacrimali_unaffiliated2
rind of pomegarnettes	De Fistula in Lacrimali_unaffiliated2

hoot water	De Ulceribus et Plagis Cornee_unaffiliated1
sugre	De Ulceribus et Plagis Cornee_unaffiliated1
hony	De Ulceribus et Plagis Cornee_unaffiliated1
rosis	De Ruptura Cornee_unaffiliated1
plantago	De Ruptura Cornee_unaffiliated1
bursa pastoris	De Ruptura Cornee_unaffiliated1
water	De Ruptura Cornee_unaffiliated1
ei yolk	De Cancro in Cornea_unaffiliated1
oil of rosis	De Cancro in Cornea_unaffiliated1
womman mylke	De Cancro in Cornea_unaffiliated1
orobus	De Ulceribus Palpebrarum et Sanie_unaffiliated1
rind of pomegarnettes	De Ulceribus Palpebrarum et Sanie_unaffiliated1
whit wyn	De Ulceribus Palpebrarum et Sanie_unaffiliated1
sal water	De Ulceribus Palpebrarum et Sanie_unaffiliated2
aloen	De Tumore Inflacione et Pinguedine Palpebrarum_unaffiliated1
acacia	De Tumore Inflacione et Pinguedine Palpebrarum_unaffiliated1
crocus	De Tumore Inflacione et Pinguedine Palpebrarum_unaffiliated1
vinegre	De Tumore Inflacione et Pinguedine Palpebrarum_unaffiliated1
vinegre	De Tumore Inflacione et Pinguedine Palpebrarum_unaffiliated2
sal water	De Tumore Inflacione et Pinguedine Palpebrarum_unaffiliated2
acacia	De Tumore Inflacione et Pinguedine Palpebrarum_unaffiliated3
bole armoniac	De Tumore Inflacione et Pinguedine Palpebrarum_unaffiliated3
juse of plantago	De Tumore Inflacione et Pinguedine Palpebrarum_unaffiliated3
water	De Tumore Inflacione et Pinguedine Palpebrarum_unaffiliated4
vinegre	De Tumore Inflacione et Pinguedine Palpebrarum_unaffiliated4
oil of camomille	De Tumore Inflacione et Pinguedine Palpebrarum_unaffiliated4
wex	De Tumore Inflacione et Pinguedine Palpebrarum_unaffiliated4
juse of plantago	De Pascionibus Oris_Carbunculus, wilde fuyre_unaffiliated1
coriandre	De Pascionibus Oris_Carbunculus, wilde fuyre_unaffiliated1
morelle	De Pascionibus Oris_Carbunculus, wilde fuyre_unaffiliated1
wyn of pomegarnettes	De Pascionibus Oris_Carbunculus, wilde fuyre_unaffiliated1
sumac	De Pascionibus Oris_unaffiliated2
galle	De Pascionibus Oris_unaffiliated2

psidia	De Pascionibus Oris_unaffiliated2
balaustia	De Pascionibus Oris_unaffiliated2
mastic	De Pascionibus Oris_unaffiliated2
olibanum	De Pascionibus Oris_unaffiliated2
hony	De Pascionibus Oris_unaffiliated2
vinegre	De Pascionibus Oris_unaffiliated2
agrimonie	De Pascionibus Oris_unaffiliated3
wyn	De Pascionibus Oris_unaffiliated3
hony	De Pascionibus Oris_unaffiliated3
malve	De Lentiginibus_unaffiliated1
bismalve	De Lentiginibus_unaffiliated1
farina of barlich	De Lentiginibus_unaffiliated1
otyn	De Lentiginibus_unaffiliated1
diamoron	De Pascionibus Gingivarum_unaffiliated1
crocus	De Pascionibus Gingivarum_unaffiliated1
wyn	De Pascionibus Gingivarum_unaffiliated1
galle	De Pascionibus Gingivarum_unaffiliated1
sumac	De Pascionibus Gingivarum_unaffiliated1
balaustia	De Pascionibus Gingivarum_unaffiliated1
alum	De Pascionibus Gingivarum_unaffiliated1
hony	De Pascionibus Gingivarum_unaffiliated1
sal	De Pascionibus Gingivarum_unaffiliated2
alum	De Pascionibus Gingivarum_unaffiliated2
galle	De Pascionibus Gingivarum_unaffiliated2
vinegre	De Pascionibus Gingivarum_unaffiliated2
plantago	De Pascionibus Gingivarum_unaffiliated3
portulaca	De Pascionibus Gingivarum_unaffiliated3
psidia	De Pascionibus Gingivarum_unaffiliated3
balaustia	De Pascionibus Gingivarum_unaffiliated3
galle	De Pascionibus Gingivarum_unaffiliated3
olibanum	De Pascionibus Gingivarum_unaffiliated4
fenigrec	De Pascionibus Gingivarum_unaffiliated4
juse of apium	De Pascionibus Gingivarum_unaffiliated4

hony	De Pascionibus Gingivarum_unaffiliated4
hoot water	De Apostemate Mamillarum_unaffiliated1
vinegre	De Apostemate Mamillarum_unaffiliated1
oil of rosis	De Apostemate Mamillarum_unaffiliated1
farina of faba	De Apostemate Mamillarum_unaffiliated2
barlich	De Apostemate Mamillarum_unaffiliated2
camomille	De Apostemate Mamillarum_unaffiliated2
sandali	De Apostemate Mamillarum_unaffiliated2
vinegre	De Apostemate Mamillarum_unaffiliated2
whit wyn	De Apostemate Mamillarum_unaffiliated2
