# Supplementary figures and images for: Data Mining a Medieval Medical Text Reveals Patterns in Ingredient Choice That Reflect Biological Activity against Infectious Agents
Source: mBio. 2020 Feb 11;11(1):e03136-19. doi: 10.1128/mBio.03136-19 (PMC7018648; doi:10.1128/mBio.03136-19)

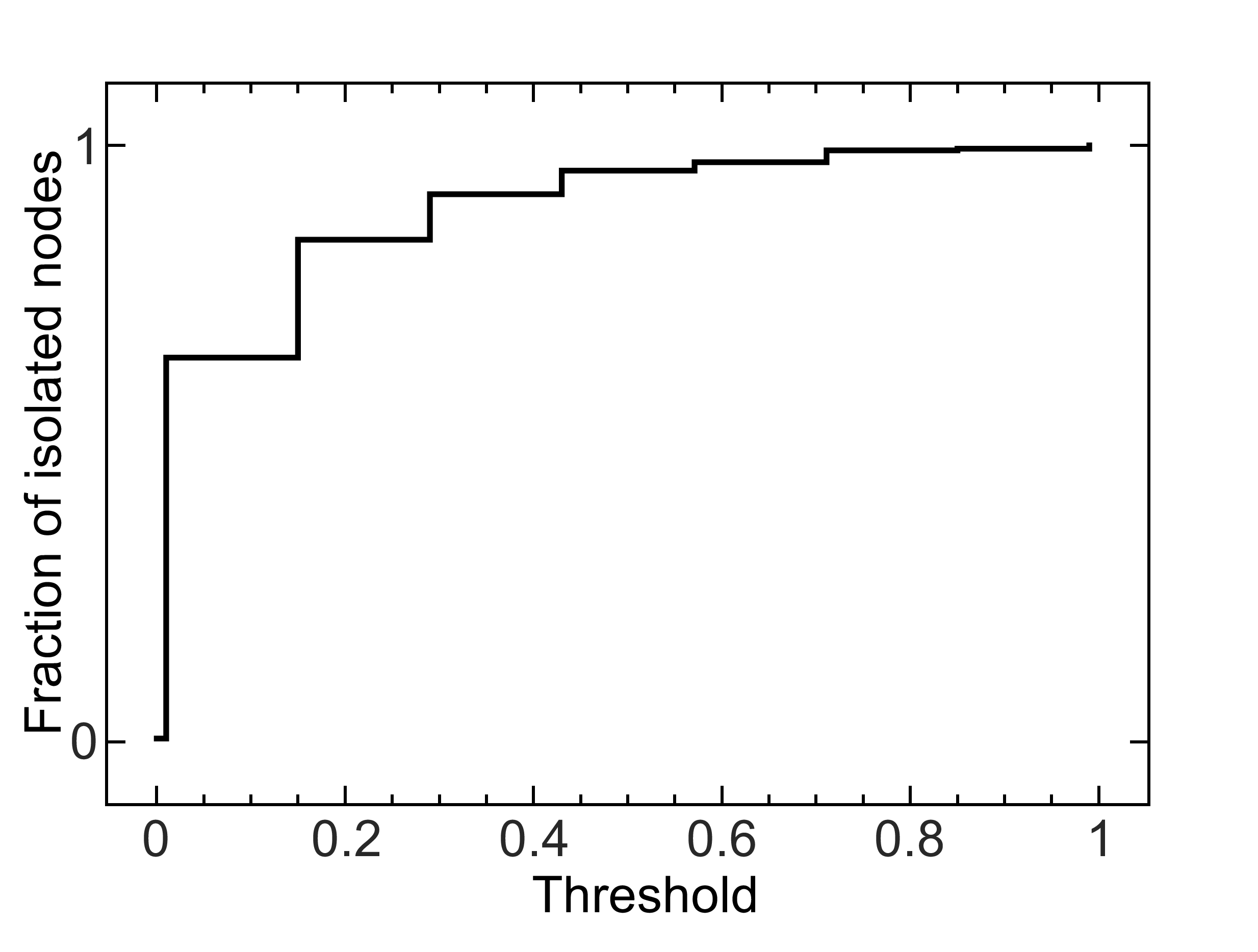

Supplement: FIG S1 [file mBio.03136-19-sf001.tif]
