# Supplementary material for: Data Mining a Medieval Medical Text Reveals Patterns in Ingredient Choice That Reflect Biological Activity against Infectious Agents
Source: mBio. 2020 Feb 11;11(1):e03136-19. doi: 10.1128/mBio.03136-19 (PMC7018648; doi:10.1128/mBio.03136-19)
Supplement: TEXT S1 [file mBio.03136-19-s0001.docx]

GROUP 1: contain two significant combinations of ingredients (*aloen*+*sarcocolla* and *olibanum*+*sumac*)

1) De Fistula in Lacrimali_unaffiliated2
2) De Lacrimis_1
3) De Lacrimis_3

GROUP 2: contain one significant combination of ingredients and some other single ingredients identified as important in the overall network (such as *galle* or *hony*)

4) De Albugine et Macula et Cicatrice in Oculis_4: aloen+sarcocolla, galle

5) De Excrescencia et Diminucione Carnis in Conjunctiva_1: aloen, olibanum+sumac

6) De Panniculo in Conjunctiva_unaffiliated1: aloen+sarcocolla, vinegre

7) De Pascionibus Oris_unaffiliated2: olibanum+sumac, balaustia, galle, hony, vinegre

8) De Ulcere et Sanie in Conjunctiva_1: aloen+sarcocolla, olibanum

9) De Vulneribus_1: aloen+sarcocolla, hony

GROUP 3: contain one significant combination of ingredients

10) De Carnositate in Palpebra Exteriori_1: aloen+sarcocolla

11) De Herpite Estiomens Cancro seu Lupo_5: aloen+sarcocolla

12) De Lacrimis_2: olibanum+sumac

13) De Obtalmia_unaffiliated4: aloen+sarcocolla

14) De Ruptura Cornee_1: aloen+sarcocolla

15) De Scabie Sicca cum Ulceribus_2: aloen+sarcocolla

GROUP 4: contain four single ingredients identified as important in the overall network

16) De Herpite Estiomens Cancro seu Lupo_3: aloen, galle, hony, vinegre

17) De Pascionibus Gingivarum_1: balaustia, galle, hony, vinegre

18) De Pascionibus Gingivarum_unaffiliated1: sumac, balaustia, galle, hony

GROUP 5: contain three single ingredients identified as important in the overall network

19) De Fistula_1: aloen, hony, vinegre

20) De Fistula in Lacrimali_unaffiliated1: sumac, galle, hony

21) De Herisipula, Antrace, Carbunculo, et Sacri Ignis_unaffiliated2: olibanum, hony, vinegre

22) De Herpite Estiomens Cancro seu Lupo_4: sarcocolla, olibanum, balaustia

23) De Inpetigine et Serpigine_1: galle, hony, vinegre

24) De Lepra_11: aloen, hony, vinegre

25) De Pascionibus Gingivarum_2: balaustia, galle, hony

26) De Scabie_10: aloen, olibanum, balaustia

GROUP 6: contain two single ingredients identified as important in the overall network

27) De Herpite Estiomens Cancro seu Lupo_5: olibanum, galle

28) De Pascionibus Gingivarum_3: balaustia, galle

29) De Pascionibus Gingivarum_unaffiliated2: galle, vinegre

30) De Pascionibus Gingivarum_unaffiliated3: balaustia, galle

31) De Pascionibus Gingivarum_unaffiliated4: olibanum, hony

32) De Pruritu Palpebrarum_1: sarcocolla, sumac

33) De Scabie Pruritu, Asperitate et Rubore_1: aloen, hony

34) De Scabie Sicca cum Ulceribus_1: aloen, vinegre

35) De Tumore Inflacione et Pinguedine Palpebrarum_unaffiliated1: aloen, vinegre

36) De Vulneribus_2: aloen, olibanum

37) De Vulneribus_3: galle, hony

GROUP 7: contain one single ingredient identified as important in the overall network

38) De Albugine et Macula et Cicatrice in Oculis_1: hony

39) De Apostemate Mamillarum_2: hony

40) De Apostemate Mamillarum_unaffiliated1: vinegre

41) De Apostemate Mamillarum_unaffiliated2: vinegre

42) De Apostematibus Calidis Generatis Per Uiam Adustionis_2: hony

43) De Casu Pilorum Palpebris_1: hony

44) De Herisipula, Antrace, Carbunculo, et Sacri Ignis_unaffiliated1: vinegre

45) De Herisipula, Antrace, Carbunculo, et Sacri Ignis_unaffiliated3: vinegre

46) De Herpite Estiomens Cancro seu Lupo_2: olibanum

47) De Inpetigine et Serpigine_2: vinegre

48) De Malo Mortuo_1: vinegre

49) De Nodis seu Lippa et Petrificacione in Palpebrarum_1: vinegre

50) De Noli Me Tangere_1: aloen

51) De Ordeolo_1: olibanum

52) De Pascionibus Oris_unaffiliated3: hony

53) De Perforacione, Putredione et Vermibus Dencium_1: vinegre

54) De Pustulis_1: vinegre

55) De Pustulis_2: vinegre

56) De Scabie_2: aloen

57) De Scabie_9: aloen, vinegre

58) De Tumore Inflacione et Pinguedine Palpebrarum_unaffiliated2: vinegre

59) De Tumore Inflacione et Pinguedine Palpebrarum_unaffiliated4: vinegre

60) De Ulceribus et Plagis Cornee_unaffiliated1: hony

61) De Vulneribus_4: vinegre

62) De Vulneribus_5: vinegre
